# Macrophagic Sclerostin Loop2‐ApoER2 Interaction Required by Sclerostin for Cardiovascular Protective Action

**DOI:** 10.1002/advs.202518735

**Published:** 2025-11-23

**Authors:** Luyao Wang, Xiaohui Tao, Ning Zhang, Xin Yang, Hewen Jiang, Xiaofei Li, Shenghang Wang, Shijian Ding, Sifan Yu, Huarui Zhang, Yihao Zhang, Nanxi Li, Haitian Li, Zhanghao Li, Xiaoxin Wen, Meiheng Sun, Chuanxin Zhong, Jin Liu, Yuanyuan Yu, Xianghang Luo, Tao Zhang, Shu Zhang, Péter Ferdinandy, Yu Huang, Daqing Ma, Aiping Lu, Baoting Zhang, Ge Zhang

**Affiliations:** ^1^ Law Sau Fai Institute for Advancing Translational Medicine in Bone and Joint Diseases (TMBJ) School of Chinese Medicine Hong Kong Baptist University Hong Kong SAR 000000 China; ^2^ Guangdong‐Hong Kong‐Macao Greater Bay Area International Research Platform for Aptamer‐based Translational Medicine and Drug Discovery (HKAP) Hong Kong SAR 000000 China; ^3^ Institute of Precision Medicine and Innovative Drug Discovery (PMID) School of Chinese Medicine Hong Kong Baptist University Hong Kong SAR 000000 China; ^4^ Institute of Integrated Bioinformedicine and Translational Science (IBTS) School of Chinese Medicine Hong Kong Baptist University Hong Kong SAR 000000 China; ^5^ School of Chinese Medicine Faculty of Medicine The Chinese University of Hong Kong Hong Kong SAR 000000 China; ^6^ Xiangya Hospital of Central South University Hunan 410008 China; ^7^ Shanghai Institute of Technical Physics Chinese Academy of Sciences Shanghai 200083 China; ^8^ The Key Laboratory of Aerospace Medicine Ministry of Education Air Force Medical University Xi'an Shanxi 710000 China; ^9^ Department of Pharmacology and Pharmacotherapy Semmelweis University Budapest 1007 Hungary; ^10^ Center for Pharmacology and Drug Research and Development Semmelweis University Budapest 1007 Hungary; ^11^ Pharmahungary Group Szeged 6700 Hungary; ^12^ Biomedical Sciences and Vascular Biology City University of Hong Kong Hong Kong SAR 000000 China; ^13^ Perioperative and Systems Medicine Laboratory and Department of Anesthesiology Children's Hospital Zhejiang University School of Medicine National Clinical Research Centre for Child Health Hangzhou 242332 China; ^14^ Division of Anaesthetics Pain Medicine and Intensive Care Department of Surgery and Cancer Faculty of Medicine Imperial College London Chelsea and Westminster Hospital London E1 7AD UK

**Keywords:** ApoER2 (LRP8), cardiovascular events, macrophage, sclerostin

## Abstract

Therapeutic antibody against sclerostin loop2 promoted bone formation in postmenopausal osteoporosis but caused severe cardiovascular events in clinical applications. The studies of atherosclerosis and aortic aneurysm in *SOST^ki^.ApoE^−/−^* mice and *sost^−/−^*.*ApoE^−/−^* mice collectively indicated the cardiovascular protective action of sclerostin. However, how sclerostin exerts cardiovascular protective action remains unclear. In this study, ApoER2 (LRP8) is notably identified as a novel transmembrane receptor for sclerostin in macrophages. Mechanistically, blockade of macrophagic sclerostin loop2‐ApoER2 interaction attenuates the suppressive effects of sclerostin on NF‐κB nuclear translocation, phosphorylation, and mRNA expression in macrophages, reduces the promotive effects of sclerostin on macrophage conversion to anti‐inflammatory phenotypes, and inhibits the preventive effects of sclerostin on atherosclerosis and aortic aneurysm in *ApoE^−/−^* mice. Together, macrophagic sclerostin loop2‐ApoER2 interaction is required by sclerostin to suppress inflammatory responses, atherosclerosis, and aortic aneurysm in *ApoE^−/−^* mice. Sclerostin plays a compensatory protective role in the cardiovascular system when *ApoE* is absent or mutated. Translationally, it provided critical pre‐clinical evidence regarding the prediction of cardiovascular risk populations (e.g.*, APOE* variants) for the marketed antibody against sclerostin loop2. Importantly, targeting sclerostin while preserving macrophagic sclerostin loop2‐ApoER2 interaction would offer the next generation of precise sclerostin inhibition strategy without cardiovascular safety concern, while promoting bone formation.

## Graphical abstract

Sclerostin loop2‐ApoER2 interaction in macrophages is required by sclerostin to suppress NF‐κB nuclear translocation and phosphorylation, to promote macrophage conversion into anti‐inflammatory subtypes in atherosclerotic aortas, as well as to prevent atherosclerosis and aortic aneurysm development in *ApoE^−/−^* mice.

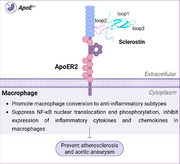

## 1 Introduction

Sclerostin, which negatively regulates bone formation, is a novel target for bone anabolic therapy.^[^ ^1^ ^]^ Structurally, sclerostin contains three central loops (loop1, loop2, and loop3), with loop2 being the primary target of the marketed therapeutic antibody for treatment of postmenopausal osteoporosis.^[^ ^2^ ^]^ However, clinical trials (BRIDGE and ARCH) revealed severe cardiovascular risks, including myocardial infarction and stroke.^[^ ^3^, ^4^, ^5^, ^6^, ^7^, ^8^, ^9^ ^]^ The US Food and Drug Administration (US‐FDA) gives a black‐boxed warning on the risk of heart attack, stroke, and cardiovascular death (FDA Press Announcements). More rigorously, the European Medicines Agency (EMA) restricts its use only in severe postmenopausal osteoporosis patients who have no history of heart attack and stroke (EMA Documents). Meta‐analysis of cardiovascular outcome data from the phase III randomized controlled trials of the marketed sclerostin antibody,^[^ ^10^ ^]^ meta‐analysis of cardiovascular events of BMD‐increasing *SOST* variants in the U.K. Biobank (UKB) ^[^ ^10^ ^]^ and genome‐wide association study (GWAS) meta‐analysis of sclerostin levels in Europeans^[^ ^11^, ^12^ ^]^ indicated that therapeutically and genetically lowered sclerostin consistently led to a higher risk of cardiovascular events.

Preclinical evidence further indicated the cardiovascular protective effect of sclerostin. Sclerostin protein level in the human aortic aneurysm (AA) samples was significantly lower than that in the control samples from normal human abdominal aortas.^[^ ^13^ ^]^ Consistently, the sclerostin protein level in the suprarenal aorta samples from mice that developed AA was significantly lower than that from mice that did not develop AA after four weeks of Angiotensin II (AngII) infusion.^[^ ^13^ ^]^ It was previously found that transgenic introduction of sclerostin inhibited inflammatory responses, atherosclerosis, and AA development in *SOST^ki^.ApoE^−/−^* mice with AngII infusion.^[^ ^13^, ^14^, ^15^ ^]^ In this study, sclerostin knockout dramatically aggravated inflammatory responses, atherosclerosis, and AA in *sost^−/−^*.*ApoE^−/−^* mice with AngII infusion. The above clinical and preclinical data indicated the protective action of sclerostin in cardiovascular system. However, how sclerostin exerts cardiovascular protective action remains unclear.

ApoER2, also known as LRP8, is a transmembrane receptor belonging to the low‐density lipoprotein receptor‐related protein (LRP) family.^[^ ^16^ ^]^ The published clinical data showed that ApoER2‐R952Q variants had a 2‐fold greater susceptibility to cardiovascular diseases^[^ ^17^ ^]^ and were associated with early onset of myocardial infarction.^[^ ^18^, ^19^, ^20^ ^]^ ApoER2 deficiency led to vascular inflammation and lesion progression in atherosclerosis of mice.^[^ ^21^ ^]^ It indicated the critical role of ApoER2 in protecting cardiovascular system.

In this study, we, for the first time, investigated the key structural domain (loop2) within sclerostin for its cardiovascular protective action,^[^ ^2^, ^14^, ^15^ ^]^ identified the cells (macrophages) and the receptor protein (ApoER2) that mediated the inflammation suppressive effects (suppressing NF‐κB nuclear translocation, phosphorylation and mRNA expression, promoting macrophage conversion to anti‐inflammatory phenotypes) and cardiovascular events preventive action (preventing atherosclerosis and aortic aneurysm) of sclerostin in *ApoE^−/−^* mice.^[^ ^22^, ^23^, ^24^, ^25^ ^]^ Translating molecular understanding into therapeutic implications, targeting sclerostin while preserving macrophagic sclerostin loop2‐ApoER2 interaction would offer the next generation of precise sclerostin inhibition strategy with no safety concern in the cardiovascular system, for promoting bone formation.

## 2 Results

### 2.1 ScRNA‐seq Analysis of Aortic Macrophages in *SOST^ki^.ApoE^−/−^* Mice and *ApoE^−/−^* Mice

In our preliminary work, sclerostin knock‐in significantly inhibited inflammatory responses, atherosclerosis, and aortic aneurysm (AA) in *SOST^ki^.ApoE^−/−^* mice with AngII infusion, when compared to *ApoE^−/−^* mice with AngII infusion.^[^ ^11^ ^]^ Here, single‐cell RNA sequencing (scRNA‐seq) analysis was performed on the aortic macrophage niche from *SOST^ki^.ApoE^−/−^* mice and *ApoE^−/−^* mice to investigate the underlying mechanisms behind the cardiovascular protective action of sclerotin.

Quality control metrics confirmed the robustness of the scRNA‐seq data. Most cells exhibited an appropriate number of detected genes (1000–6000) and low mitochondrial RNA content, indicating high cell viability (Figure S1A–C, Supporting Information). Scatter plots of the number of genes detected per cell (n_genes_by_counts) versus total counts (total_counts) confirmed consistent quality across the dataset, with minimal outliers (Figure S1D,F, Supporting Information). Additionally, low mitochondrial RNA proportion indicated negligible mitochondrial stress (Figure S1E,F, Supporting Information). Dispersion plots highlighted the highly variable genes after normalization (Figure S1G,H, Supporting Information). Variance ratio analysis showed that the first 10 principal components captured most of the biologically relevant variation in the dataset (Figure S1I, Supporting Information), forming the basis for dimensionality reduction and clustering.

**Figure 1:**
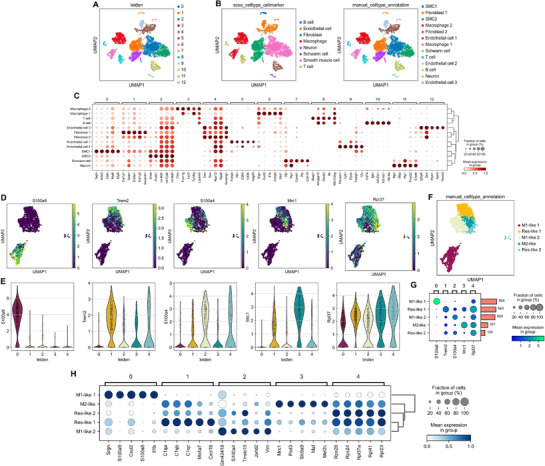
Single‐cell RNA sequencing analysis of aortic immune and stromal cells in *ApoE^−/−^* mice with AngII infusion and *SOST^ki^.ApoE^−/−^* mice with AngII infusion. (A) UMAP plot showing cell clusters identified by the Leiden algorithm, labeled 0 to 12, representing diverse cellular populations in aortic tissue. (B) Cell type annotation based on canonical markers, identifying major populations including macrophages, fibroblasts, endothelial cells, smooth muscle cells, T cells, B cells, and Schwann cells (left), as well as subdividing major populations into specific subtypes such as M1‐like pro‐inflammatory and M2‐like anti‐inflammatory macrophages, multiple endothelial subtypes, and multiple fibroblast subtypes (right). (C) Dot plot showing expression of selected marker gene across identified clusters. Dot size represented the percentage of cells in each cluster expressing the gene, while color intensity indicated the mean expression level. (D) UMAP plots showing the expression of genes including S100a8, Trem2, S100a4, Mrc1, and Rpl37 across macrophage clusters. (E) Violin plots showing expression distribution of marker genes (S100a8, Trem2, S100a4, Mrc1, and Rpl37) across macrophage clusters based on Leiden clustering. (F) UMAP plot of macrophage subtypes M1‐like 1, M1‐like 2, M2‐like, Res‐like 1, and Res‐like 2, based on manual annotation. Note: Clusters 0, 1, 2, 3, and 4 represented the following macrophage sub‐types: Cluster 0: M1‐like 1, Cluster 1: M1‐like 2, Cluster 2: M2‐like, Cluster 3: Res‐like 1, and Cluster 4: Res‐like 2. (G) Dot plot summarizing the expression of key marker genes (S100a8, Trem2, S100a4, Mrc1, and Rpl37) across macrophage subtypes. Dot size represented the fraction of cells expressing each gene, while color intensity indicated mean expression. (H) Dot plot of the TOP 5 highly expressed genes across macrophage subtypes. Dot size indicated the fraction of cells expressing each gene, and color intensity represented mean expression.

Subsequently, we identified 13 distinct cell clusters within the aortic tissue using Leiden clustering (**Figure** **1**A). These clusters represented major cell types, including B cells, endothelial cells, fibroblasts, macrophages, neurons, schwann cells, smooth muscle cells, and T cells (Figure 1B,C). Further, 5 macrophages sub‐clusters were identified based on re‐clustering analysis (Figure 1D–H, Figure S2, Supporting Information). Ridge plots provided additional insights into the distribution of key genes across macrophage subtypes (Figure S2, Supporting Information). Violin plots demonstrated the variability in gene expression across individual Leiden clusters, emphasizing differences among macrophage subtypes (Figure 1E). In detail, M1 pro‐inflammatory‐like macrophages 1 (M1‐like 1) exhibited high gene expression level of S100a8; M1 pro‐inflammatory‐like macrophages 2 (M1‐like 2) exhibited high gene expression level of S100a4; M2 anti‐inflammatory‐like macrophages (M2‐like) exhibited high gene expression level of Mrc1; Trem2‐like resident macrophages (Res‐like 1) exhibited high gene expression level of Trem2; resident‐like macrophages (Res‐like 2) exhibited high gene expression level of Rpl37 (Figure 1E,F). Moreover, the distribution of the above marker gene expression across macrophage subtypes, including the proportion of cells expressing each gene, mean expression levels, and corresponding cell counts, provided a comprehensive view of the heterogeneity within macrophage populations (Figure 1G). The expression levels of the TOP 5 highly expressed genes within each macrophage subtype and the corresponding cellular proportions were also depicted to provide a deeper understanding of the unique transcriptional profiles and functional diversity of the aforementioned subtypes of macrophages (Figure 1H).

**Figure 2:**
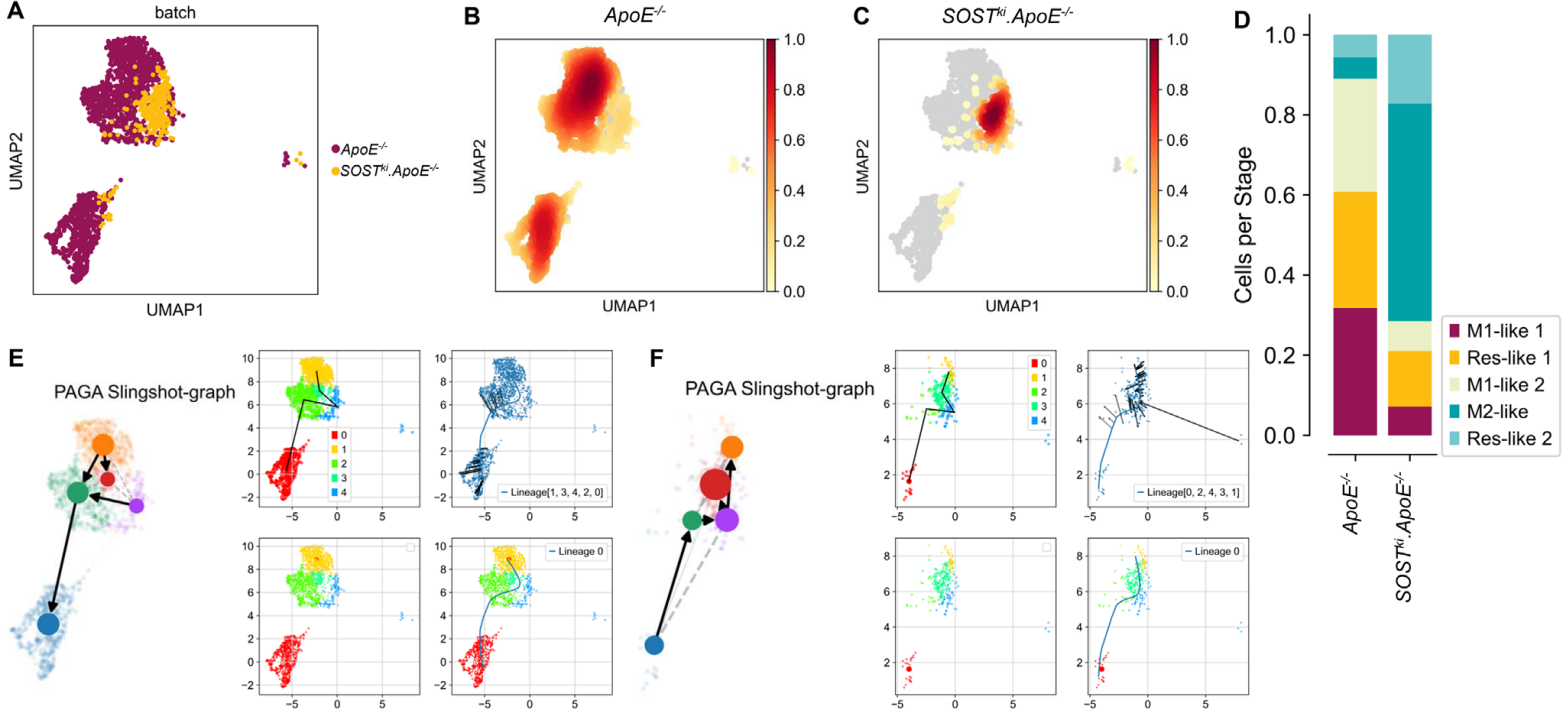
Comparative analysis of distribution patterns, subtype proportions, and differentiation trajectories of macrophages between *ApoE^−/−^* mice with AngII infusion and *SOST^ki^.ApoE^−/−^* mice with AngII infusion. (A) UMAP plot comparing differences in transcriptional profiles and spatial distribution of aortic macrophage subtypes between *ApoE^−/−^* mice (prune) and *SOST^ki^.ApoE^−/−^* mice (yellow). (B) Density map showing the distribution of macrophages from aortas in *ApoE^−/−^* mice. Higher cell density was indicated by red areas. (C) Density map showing the distribution of macrophages from aortas in *SOST^ki^.ApoE^−/−^* mice. Higher cell density was indicated by red areas. (D) Stacked bar plot comparing the difference in proportion of macrophage subtypes between *ApoE^−/−^* mice and *SOST^ki^.ApoE^−/−^* mice. (E) PAGA Slingshot graph for aortic macrophages in *ApoE^−/−^* mice, indicating differentiation trajectories toward pro‐inflammatory macrophage subtypes. (F) PAGA Slingshot graph for aortic macrophages in *SOST^ki^.ApoE^−/−^* mice, indicating differentiation trajectories toward anti‐inflammatory macrophage subtypes. Note: Clusters 0, 1, 2, 3, and 4 represented the following macrophage sub‐types: Cluster 0: M1‐like 1, Cluster 1: M1‐like 2, Cluster 2: M2‐like, Cluster 3: Res‐like 1, and Cluster 4: Res‐like 2.

Using uniform manifold approximation and projection (UMAP)‐based clustering and density mapping, we analyzed and compared the proportion of macrophages subtypes between *ApoE^−/−^* mice with AngII infusion and *SOST^ki^.ApoE^−/−^* mice with AngII infusion. Macrophages from aortas in *ApoE^−/−^* mice mainly exhibited pro‐inflammatory subtypes (M1‐like 1 and M1‐like 2) and resident subtypes (Res‐like 1) (**Figure** **2**A,B), whereas macrophages from aortas in *SOST^ki^.ApoE^−/−^* mice mainly exhibited anti‐inflammatory subtypes (M2‐like) and resident subtypes (Res‐like 2) (Figure 2A,C). *SOST^ki^.ApoE^−/−^* mice had dramatically higher proportions of M2‐like anti‐inflammatory macrophages than *ApoE^−/−^* mice (Figure 2D). Moreover, in pseudotime analysis with partition‐based graph abstraction (PAGA) Slingshot, multiple macrophage differentiation trajectories were revealed (Figure 2E,F, Figure S3A,B, Supporting Information). In *ApoE^−/−^* mice, differentiation trajectories favored M1‐like pro‐inflammatory macrophage subtype (Figure 2E, Figure S3A, Supporting Information). In *SOST^ki^.ApoE^−/−^* mice, differentiation trajectories favored M2‐like anti‐inflammatory macrophage subtype (Figure 2F, Figure S3B, Supporting Information). It indicated that sclerostin promoted the conversion of macrophages to M2‐like anti‐inflammatory subtype.

**Figure 3:**
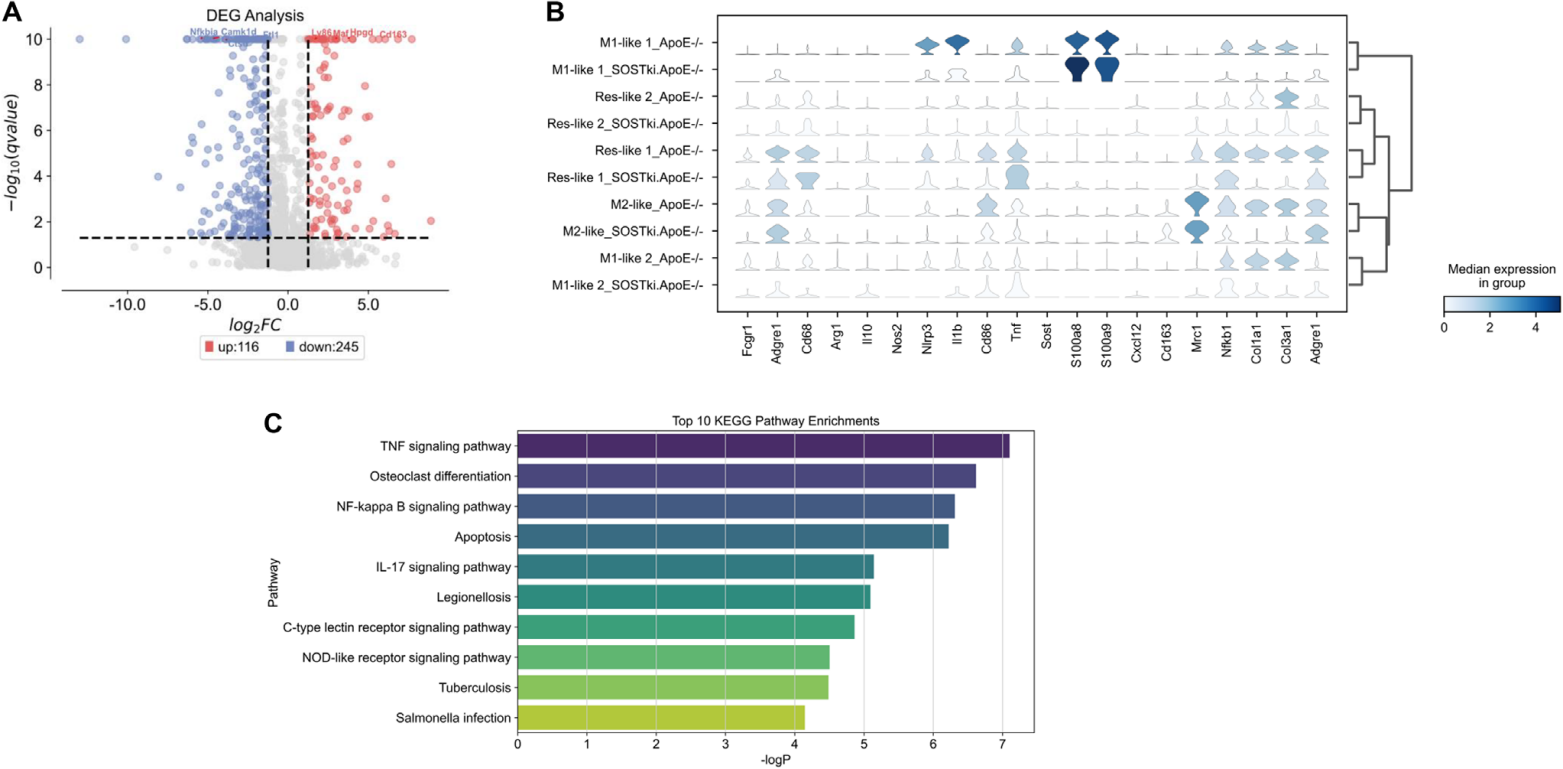
Analysis of differential gene expression and functional pathway enrichment in macrophages between *ApoE^−/−^* mice with AngII infusion and *SOST^ki^.ApoE^−/−^* mice with AngII infusion. (A) Volcano plot showing differentially expressed genes (DEGs) in macrophages from *SOST^ki^.ApoE^−/−^* mice, compared to *ApoE^−/−^* mice. Red points represented significantly upregulated genes (116 genes) in *SOST^ki^.ApoE^−/−^* mice, blue points represented significantly downregulated genes (245 genes) in *SOST^ki^.ApoE^−/−^* mice. (B) Stacking violin plot showing expression distribution of marker genes across macrophage subtypes in *ApoE^−/−^* mice and *SOST^ki^.ApoE^−/−^* mice. (C) TOP 10 KEGG pathways enriched for differentially expressed genes across all macrophages between *ApoE^−/−^* mice and *SOST^ki^.ApoE^−/−^* mice.

Furthermore, in differential gene expression (DGE) analysis of macrophages from aortas between *SOST^ki^.ApoE^−/−^* mice and *ApoE^−/−^* mice, 116 genes were upregulated, and 245 genes were downregulated, after sclerostin knock‐in (**Figure** **3**A). Thereinto, the anti‐inflammatory biomarker *Cd163* was one of the Top 5 upregulated genes after sclerostin knock‐in, while the inflammation‐driven factor *Nfκb1* ^[^ ^22^, ^26^ ^]^ was one of the Top 5 downregulated genes (Figure 3A). Consistently in stacking violin plots, the gene expression level of *Nfκb1* was downregulated in all subtypes of macrophages from aortas after sclerostin knock‐in (Figure 3B). Moreover, in Kyoto Encyclopedia of Genes and Genomes (KEGG) pathway enrichment analysis based on the above differential genes of macrophages from aortas between *SOST^ki^. ApoE^−/−^* mice and *ApoE^−/−^* mice, NF‐κB signaling pathway was one of the Top 10 enriched pathways (Figure 3C). Together, the sclerostin knock‐in likely promotes conversion of macrophages to M2‐like anti‐inflammatory subtype, inhibits macrophagic NF‐κB signaling pathway‐driven inflammatory responses in *ApoE^−/−^* mice with AngII infusion.

### 2.2 Suppressive Effects of Sclerostin on Inflammatory Responses Were Dependent on ApoER2

**Figure 4:**
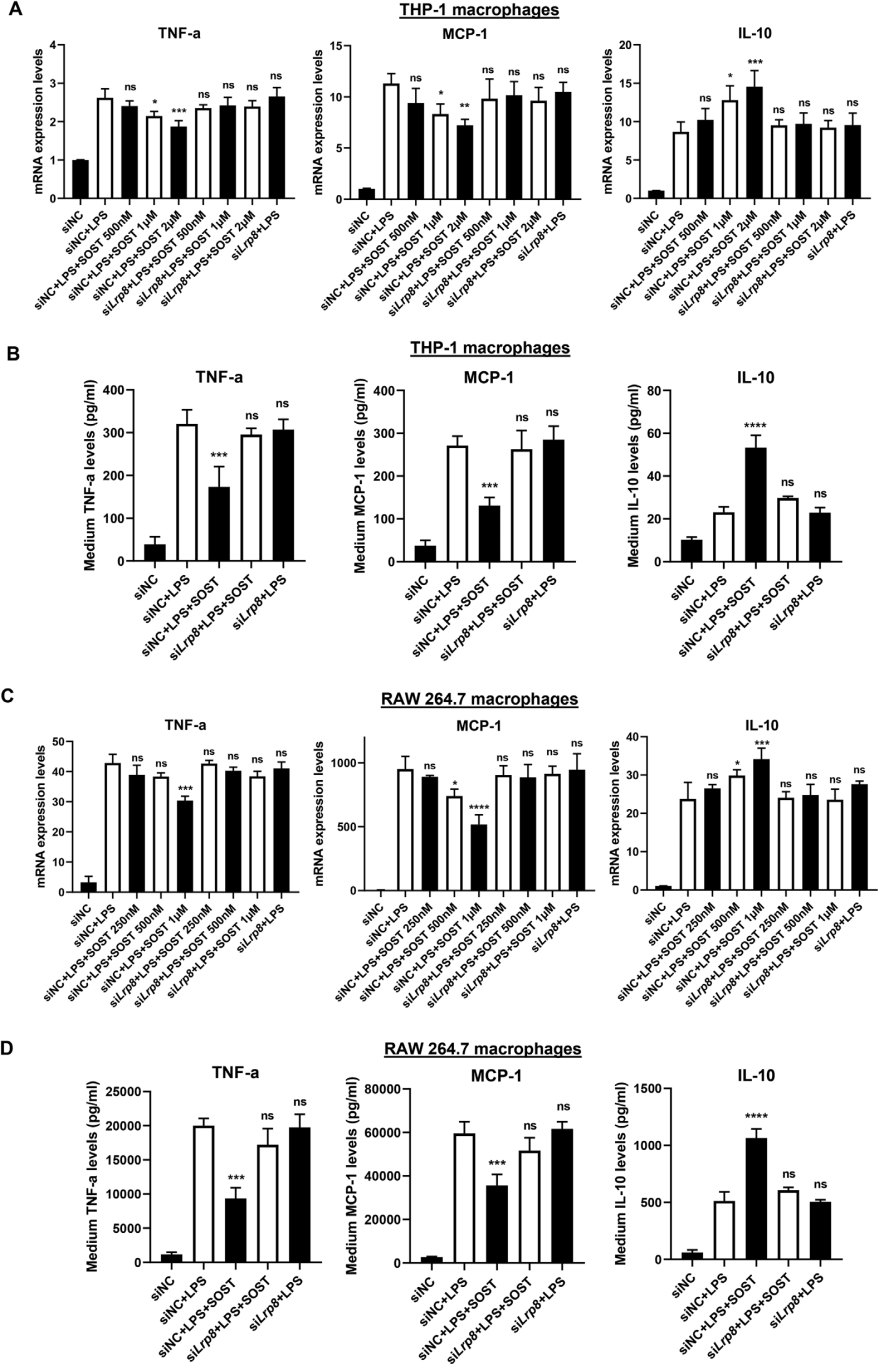
The suppressive effects of sclerostin on inflammatory responses were dependent on ApoER2 in both human macrophages and mouse macrophages in vitro. (A) The mRNA expression of TNF‐α (left), MCP‐1 (middle), and IL‐10 (right) in human differentiated macrophages (THP‐1) with/without LPS (100 ng/mL) induction (n = 3 per group). (B) The medium protein levels of TNF‐α (left), MCP‐1 (middle), and IL‐10 (right) in human differentiated macrophages (THP‐1) with/without LPS (100 ng/mL) induction (n = 3 per group). (C) The mRNA expression of TNF‐α (left), MCP‐1 (middle), and IL‐10 (right) in mouse macrophages (RAW 264.7) with/without LPS (1 µg/mL) induction (n = 3 per group). (D) The medium protein levels of TNF‐α (left), MCP‐1 (middle), and IL‐10 (right) in mouse macrophages (RAW 264.7) with/without LPS (1 µg/mL) induction (n = 3 per group). Data were expressed as mean ± standard deviation. One‐way ANOVA with Tukey's post‐hoc test versus siNC+LPS group was used to determine the intergroup differences. All tests were two‐sided. ^ns^ P > 0.05, * P < 0.05, ** P < 0.01, *** P < 0.001, **** P < 0.0001. Note: SOST: sclerostin; si*Lrp8*: small interfering RNA targeting ApoER2 (encoded by *Lrp8*); siNC: negative control small interfering RNA; LPS: lipopolysaccharide; TNF‐α: tumor necrosis factor alpha; MCP‐1: monocyte chemoattractant protein‐1; IL‐10: interleukin‐10.

ApoE was reported to promote macrophage conversion from pro‐inflammatory phenotype to anti‐inflammatory phenotype, suppress NF‐κB‐driven inflammation in macrophages, and inhibit atherosclerosis via ApoER2 (LRP8).^[^ ^22^, ^23^, ^24^, ^25^ ^]^ We found that recombinant sclerostin inhibited both mRNA expression and protein expression/secretion of pro‐inflammatory cytokine (TNF‐α) and pro‐inflammatory chemokine (MCP‐1), promoted mRNA expression and protein expression/secretion of anti‐inflammatory cytokine (IL‐10) in both human differentiated macrophages (THP‐1) and mouse macrophages (RAW 264.7) with lipopolysaccharide (LPS) induction in vitro (**Figure** **4**). To determine whether the anti‐inflammatory effects of sclerostin were dependent on ApoER2 in vitro, we silenced the expression of ApoER2 in both human differentiated macrophages (THP‐1) (Figure S4A, Supporting Information) and mouse macrophages (RAW 264.7) (Figure S4B, Supporting Information) using human/mouse *Lrp8* siRNAs (si*Lrp8*), then treated cells with recombinant human/mouse sclerostin and induced inflammatory responses with lipopolysaccharide (LPS). The mRNA expression levels of pro‐inflammatory cytokine (TNF‐α), pro‐inflammatory chemokine (MCP‐1), and anti‐inflammatory cytokine (IL‐10) were determined by RT‐PCR. The medium protein levels of pro‐inflammatory cytokine (TNF‐α), pro‐inflammatory chemokine (MCP‐1), and anti‐inflammatory cytokine (IL‐10) were determined by ELISA. The data showed that the suppressive effects of sclerostin on pro‐inflammatory cytokine and chemokine expression/secretion, as well as the promotive effect of sclerostin on anti‐inflammatory cytokine expression/secretion in human macrophages (THP‐1) and mouse macrophages (RAW 264.7) were not detected upon *Lrp8* silencing (Figure 4A–D). It indicated that the inhibitory effects of sclerostin on inflammatory responses in both human macrophages and mouse macrophages in vitro were dependent on the presence of ApoER2.

Further, we tracked the dynamic changes of inflammatory responses in mouse RAW264.7 macrophages (with *Lrp8* silencing for shielding the influence of endogenous ApoER2, Figure S4B, Supporting Information). The RAW264.7 macrophages (si*Lrp8*) were transfected with plasmids encoding ApoER2 (*Lrp8*) or vector, followed by treatment with recombinant mouse sclerostin and induction with LPS for varying durations.

**Figure 5:**
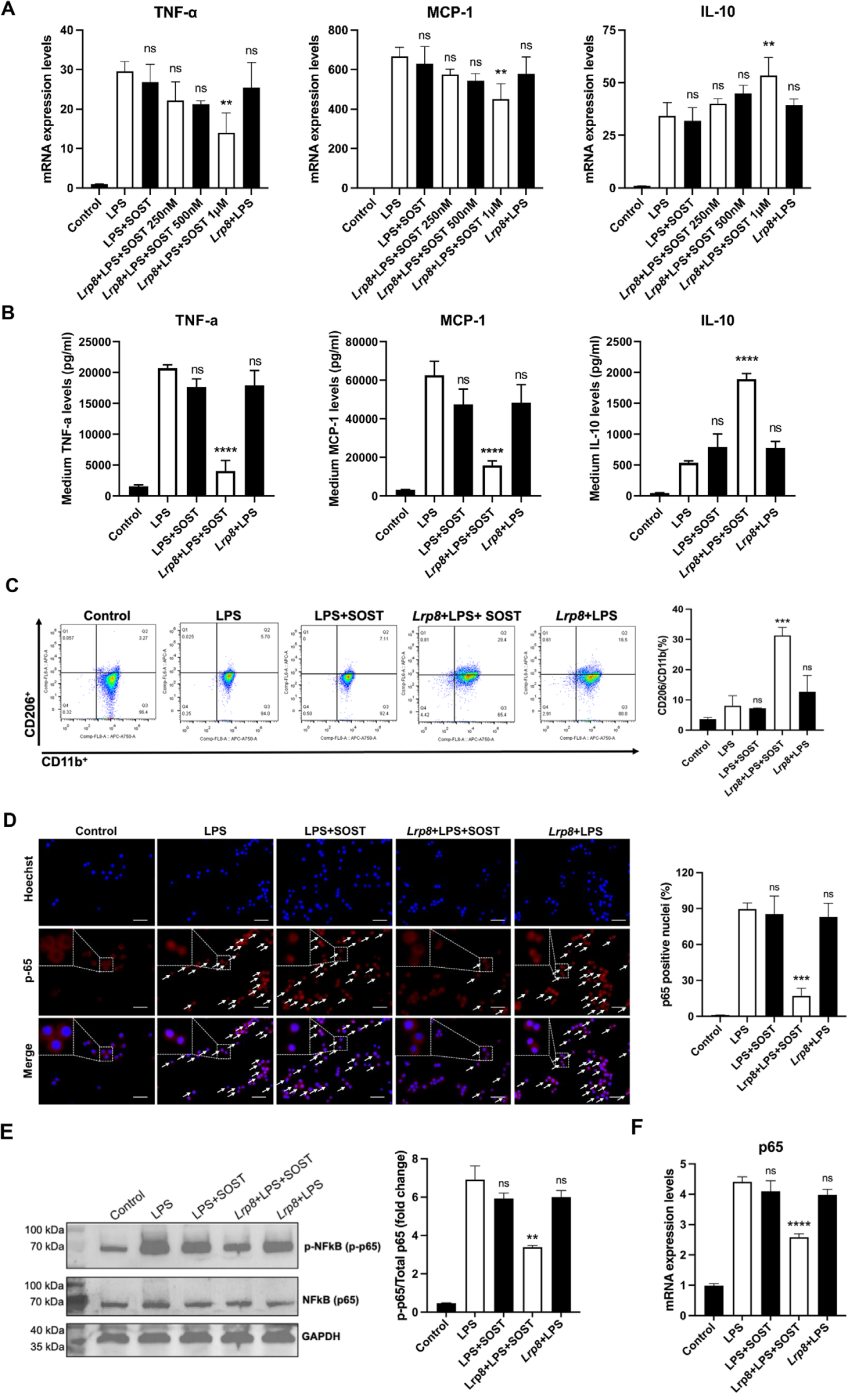
Determination of the inflammatory cytokines/chemokines expression, macrophage phenotypes conversion, NF‐κB nuclear translocation/phosphorylation/expression in macrophages in vitro. (A) The mRNA expression levels of TNF‐α (left), MCP‐1 (middle), and IL‐10 (right) in mouse macrophages (RAW264.7) with LPS (1 µg/mL) induction for 18 h (n = 3 per group). (B) The medium protein levels of TNF‐α (left), MCP‐1 (middle), and IL‐10 (right) in mouse macrophages (RAW264.7) with LPS (1 µg/mL) induction for 18 h (n = 3 per group). (C) Surface markers examined by flow cytometry depict expression of CD206 and CD11b in mouse macrophages (RAW264.7) with LPS (1 µg/mL) induction for 18 h (left). Quantification of the proportion of anti‐inflammatory phenotypes (CD206^+^) (right) (n = 2 per group). (D) Representative images (left) and quantitative analysis of immunofluorescence (right) of NF‐κB (p65) nuclear translocation in mouse macrophages (RAW264.7) with LPS (1 µg/mL) induction for 15 min. Scale bar: 50 µm. The white arrows indicated the p65‐positive nuclei (n = 3 per group). (E) The protein level of phospho‐NF‐κB (p‐p65) in mouse macrophages (RAW264.7) with LPS (1 µg/mL) induction for 6 h (left). Quantification of the density of detected bands (right) (n = 2 per group). (F) The mRNA expression of NF‐κB (p65) in mouse macrophages (RAW264.7) with LPS (1 µg/mL) induction for 6 h (n = 3 per group). Data were expressed as mean ± standard deviation. One‐way ANOVA with Tukey's post‐hoc test versus LPS group was used to determine the intergroup differences. All tests were two‐sided. ^ns^ P > 0.05, * P < 0.05, ** P < 0.01, *** P < 0.001, **** P < 0.0001. Note: SOST: sclerostin; *Lrp8*: low‐density lipoprotein receptor‐related protein 8 (encoding ApoER2); LPS: lipopolysaccharide; TNF‐α: tumor necrosis factor alpha; MCP‐1: monocyte chemoatractant protein‐1; IL‐10: interleukin‐10. CD11b: alpha chain of the macrophage‐1 receptor (macrophage marker); CD206: cluster of differentiation 206, known as mannose receptor C‐type 1 (anti‐inflammatory macrophage biomarker).

After LPS induction for varying durations, there were no significant differences in mRNA expression levels/medium protein levels of pro‐inflammatory cytokine (TNF‐α), pro‐inflammatory chemokine (MCP‐1), and anti‐inflammatory cytokine (IL‐10), macrophage phenotype proportions, NF‐κB nuclear translocation/phosphorylation/mRNA expression levels among macrophages treated with sclerostin (LPS+SOST), ApoER2‐overexpressing macrophages treated with vehicle (*Lrp8*+LPS), and untreated macrophages (LPS**) (**Figure **5**; Figure S4, Supporting Information).

After LPS induction for 18 h, compared to macrophages treated with sclerostin (LPS+SOST) or ApoER2‐overexpressing macrophages treated with vehicle (*Lrp8*+LPS), the mRNA expression levels of pro‐inflammatory cytokine (TNF‐α) and chemokine (MCP‐1) were dose‐dependently lower in ApoER2‐overexpressing macrophages treated with sclerostin (*Lrp8*+LPS+SOST), while the mRNA expression level of anti‐inflammatory cytokine (IL‐10) was dose‐dependently higher (Figure 5A). Compared to the LPS group, treatment with 1 µM recombinant sclerostin significantly suppressed mRNA expression of TNF‐α (** P < 0.01) and MCP‐1 (** P < 0.01), significantly promoted mRNA expression of IL‐10 (* P < 0.05) (Figure 5A). Based on this significant efficacy of recombinant sclerostin at 1 µM, this concentration was selected for subsequent in vitro investigations. Moreover, after LPS induction for 18 h, compared to macrophages treated with sclerostin or ApoER2‐overexpressing macrophages treated with vehicle, the medium protein levels of pro‐inflammatory cytokine (TNF‐α) and chemokine (MCP‐1) were significantly lower in ApoER2‐overexpressing macrophages treated with sclerostin, while the medium protein level of anti‐inflammatory cytokine (IL‐10) was significantly higher (Figure 5B).

In terms of macrophage conversion, after LPS induction for 12 and 18 h, the proportion of anti‐inflammatory phenotype (CD206^+^) was significantly higher in ApoER2‐overexpressing macrophages treated with sclerostin (Figure 5C, Figure S4D, Supporting Information). In terms of NF‐κB, after LPS induction for 15 min, significantly lower NF‐κB nuclear translocation was determined in ApoER2‐overexpressing macrophages treated with sclerostin (Figure 5D). After LPS induction for 30 min, an elevation in p65 protein levels was detected in the cytoplasmic fraction of ApoER2‐overexpressing macrophages treated with sclerostin, concomitant with a reduction in the nuclear fraction (Figure S4E, Supporting Information). Subsequently, after LPS induction for 6 h, significantly lower protein level of phospho‐NF‐κB (p‐p65) was determined in ApoER2‐overexpressing macrophages treated with sclerostin (Figure 5E). At the same time, significantly lower mRNA expression level of NF‐κB (p65) was determined in ApoER2‐overexpressing macrophages treated with sclerostin (Figure 5F).

Together, it suggested that the suppressive effects of sclerostin on inflammatory responses in macrophages were dependent on ApoER2 in vitro, including inhibiting NF‐κB nuclear translocation and phosphorylation, suppressing mRNA expression of NF‐κB (p65), promoting macrophage conversion to anti‐inflammatory phenotypes, inhibiting both mRNA expression and protein expression/secretion of pro‐inflammatory cytokine and chemokine, as well as promoting both mRNA expression and protein expression/secretion of anti‐inflammatory cytokine in macrophages.

### 2.3 Sclerostin Loop2 Participated in the Cardiovascular Protective Action of Sclerostin

The marketed sclerostin antibody mainly targets sclerostin loop2.^[^ ^2^ ^]^ Our previously published data indicated that specific blockade of sclerostin antibody‐sclerostin loop2 interaction attenuated the sclerostin antibody‐induced aggravation of inflammatory responses, atherosclerosis, and AA in *ApoE^−/−^* mice with AngII infusion, whereas the protective effect of sclerostin on cardiovascular system was independent of sclerostin loop3.^[^ ^14^ ^]^ Here in large‐scale GWAS analysis of clinical data from the U.K. Biobank (UKB), we identified three conditionally independent genetic variants in *SOST* loop2 locus which were associated with cardiac abnormalities (Figure S5, Supporting Information). The minor allele of rs879666342 (chr17: 43755588, G > C; G allele frequency in UKB, 2.98e‐5) was associated with higher cardiac dysrhythmias [*P = 0.013*, per C allele]. The minor allele of rs886052981 (chr17: 43755656, C > T; C allele frequency in UKB, 1.20e‐5) was associated with higher precordial pain [*P = 0.005*, per C allele]. The minor allele of rs765435662 (chr17: 43755638, G > A; G allele frequency in UKB, 2.63e‐5) was associated with higher peripheral vascular disease [*P = 0.002*, per G allele]. It indicated the association between sclerostin loop2‐specific mutations and cardiovascular abnormalities. Together, sclerostin loop2 participated in the cardiovascular protective action of sclerostin.

### 2.4 Sclerostin Loop2 Was Identified to Bind to ApoER2 in Macrophages

**Figure 6:**
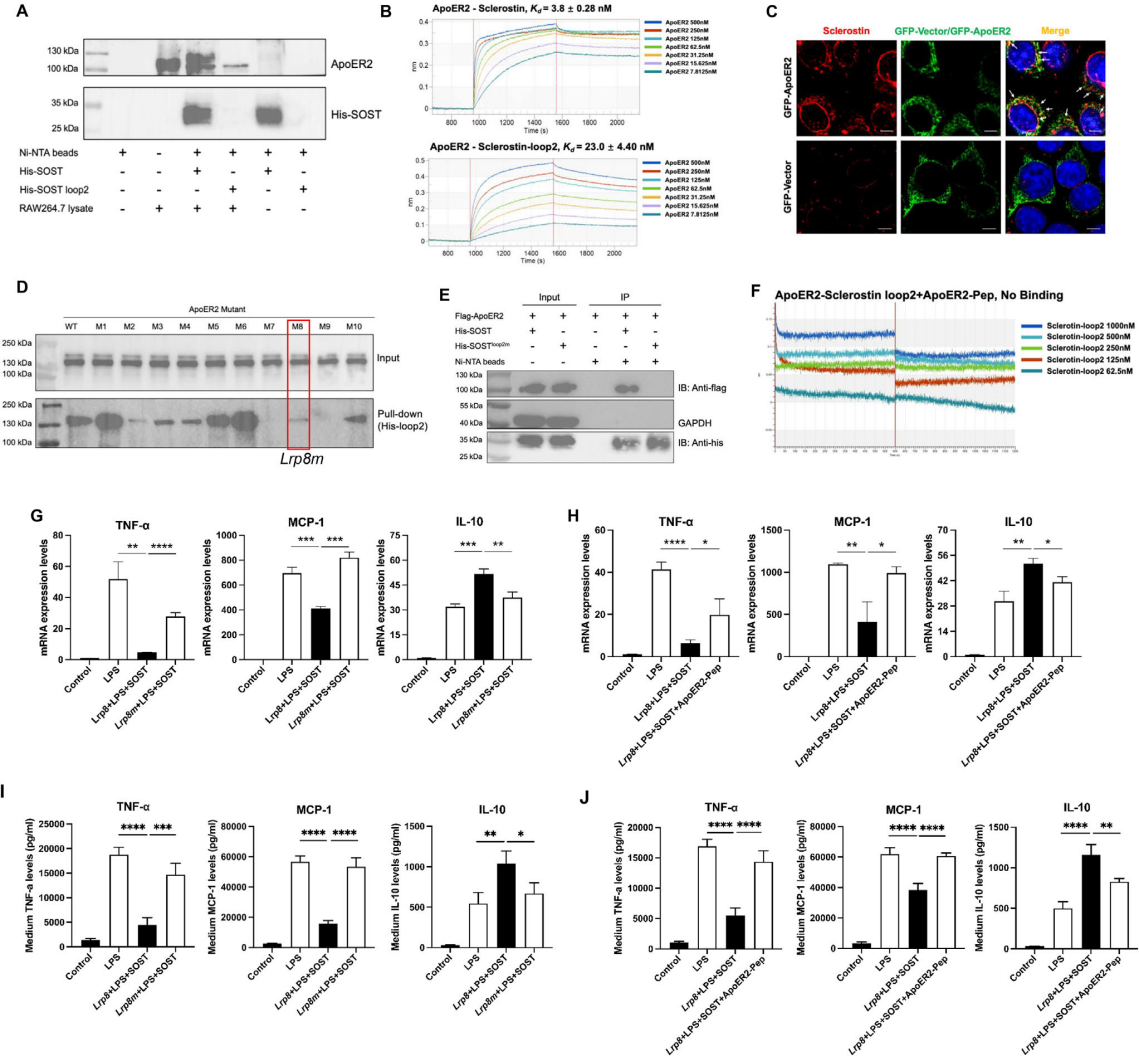
Blockade of sclerostin loop2‐ApoER2 interaction by both *Lrp8m* and ApoER2‐Pep attenuated the suppressive effects of sclerostin on inflammatory responses in macrophages in vitro. (A) Binding analysis for the interaction of ApoER2 to full‐length sclerostin and sclerostin loop2 in macrophages (RAW264.7) by pull‐down assay. (B) The binding affinity of ApoER2 to sclerostin (upper) and sclerostin loop2 (lower), determined by biolayer interferometry (BLI) analysis. (C) Confocal microscopy examination of cell surface binding of sclerostin to ApoER2 in macrophages (RAW264.7), scale bar: 5 µm. (D) Binding analysis for the interaction of ApoER2 muteins to sclerostin loop2 by pull‐down assay. (E) Binding analysis for the interaction of ApoER2 to wild‐type SOST (SOST) and SOST^loop2m^ in macrophages (RAW264.7) by co‐immunoprecipitation assay. (F) The binding affinity of ApoER2 to sclerostin with the pretreatment of ApoER2‐Pep peptide tool by BLI analysis. (G) The influence of *Lrp8m* in the effects of sclerostin on mRNA expression of TNF‐α (left), MCP‐1 (middle), and IL‐10 (right) in RAW264.7 with LPS (1 µg/mL) induction for 18 h (n = 3 per group). (H) The influence of ApoER2‐Pep in the effects of sclerostin on mRNA expression of TNF‐α (left), MCP‐1 (middle), and IL‐10 (right) in RAW264.7 with LPS (1 µg/mL) induction for 18 h (n = 3 per group). (I) The influence of *Lrp8m* in the effects of sclerostin on the protein levels of TNF‐α (left), MCP‐1 (middle), and IL‐10 (right) in RAW264.7 with LPS (1 µg/mL) induction for 18 h (n = 3 per group). (J) The influence of ApoER2‐Pep in the effects of sclerostin on the protein levels of TNF‐α (left), MCP‐1 (middle), and IL‐10 (right) in RAW264.7 with LPS (1 µg/mL) induction for 18 h (n = 3 per group). Data were expressed as mean ± standard deviation. The unpaired t‐test was used to determine the intergroup differences. All tests were two‐sided. ^ns^ P > 0.05, * P < 0.05, ** P < 0.01, *** P < 0.001, **** P < 0.0001. Note: LPS: lipopolysaccharide; TNF‐α: tumor necrosis factor alpha; MCP‐1: monocyte chemoatractant protein‐1; IL‐10: interleukin‐10.

To determine the interaction between sclerostin and ApoER2 in macrophages (RAW 264.7), we conducted pull‐down assay, co‐immunoprecipitation (co‐IP) assay, BLI analysis, and confocal microscopy examination. In pull‐down assay of RAW264.7 macrophages (RAW 264.7), FLAG‐ApoER2 was pulled by both full‐length His‐sclerostin (His‐SOST) and His‐sclerostin loop2 (His‐SOST loop2) (**Figure** **6**A), rather than His‐sclerostin loop1 (His‐SOST loop1) or His‐sclerostin loop3 (His‐SOST loop3) (Figure S6A, Supporting Information). It suggested that sclerostin loop2 bound to ApoER2. In co‐IP assay, the interaction between sclerostin and ApoER2 was determined in macrophages (RAW 264.7) (Figure 6E). Additionally, BLI analysis validated the binding of ApoER2 to sclerostin (*K_d_* = 3.8 nM) and sclerostin loop2 (*K_d_* = 23.0 nM), respectively (Figure 6B), as well as the binding of sclerostin to ApoER2 (*K_d_* = 2.1 nM) (Figure S6B, Supporting Information). In confocal microscopy examination, cell surface binding of sclerostin to ApoER2 was observed in RAW264.7 macrophages (Figure 6C).

To determine whether the interaction between sclerostin loop2 and ApoER2 was required by sclerostin to protect the cardiovascular system, we identified the interaction residues between ApoER2 and sclerostin, subsequently developed *Lrp8* mutation tool (*Lrp8m*), ApoER2‐Pep peptide tool, and *sost* mutation tool (*sost^loop2m^*) to block sclerostin loop2‐ApoER2 interaction for the following structure‐function studies.

**Identification of the interaction residues within ApoER2 to sclerostin loop2**. The interaction of full‐length/truncated sclerostin to full‐length/truncated ApoER2 (Table S1, Supporting Information) was analyzed by pull‐down assay. The data showed that the LDLR type A repeats 6 (LA6) and the LDLR type A repeats 7 (LA7) within ApoER2, loop2 within sclerostin were the key domains for sclerostin‐ApoER2 interaction (Figure 6A, Figure S6A‐D, Supporting Information). Subsequently, the interaction of sclerostin loop2 to ApoER2 mutein (Table S2, Supporting Information) with a series of mutations in ApoER2‐LA6 residues and ApoER2‐LA7 residues was analyzed by pull‐down assay. The data showed that the binding ability of sclerostin loop2 to ApoER2‐M2 (P253A, T254A, G268A, W269A, R270A), ApoER2‐M7 (P253A, T254A, L305A, I307A, N311A, Q312A, E313A), ApoER2‐M8 (P253A, T254A, G268A, W269A, R270A, N311A, Q312A, E313A) and ApoER2‐M9 (P253A, T254A, G268A, W269A, R270A, L305A, I307A) were dramatically weaker than that of sclerostin loop2 to wild‐type ApoER2 (Figure 6D). Together, P253, T254, G268, W269, R270, L305, I307, N311, Q312, E313 were the key residues within ApoER2 for interaction between sclerostin loop2 and ApoER2, which was validated in molecular docking by HDOCK (Figure S6F, Supporting Information).

***Lrp8* mutation tool for genetic blockade of sclerostin loop2‐ApoER2 interaction**. Further, we evaluated the effect of the *Lrp8* mutations encoding ApoER2 muteins, which blocked sclerostin loop2‐ApoER2 interaction, on mRNA expression levels of inflammatory cytokines and chemokines in macrophages (RAW264.7) in vitro, in the absence of sclerostin. The data showed that there were no significant differences in the mRNA expression levels of TNF‐α, MCP‐1, and IL‐10 between sclerostin‐knockout macrophages transfected with wild‐type *Lrp8* plasmid and sclerostin‐knockout macrophages transfected with *Lrp8‐m8* plasmid in vitro, after LPS induction (Figure S7A, Supporting Information). Thus, *Lrp8‐m8* mutation (encoding ApoER2‐M8 with mutations at residues: P253A, T254A, G268A, W269A, R270A, N311A, Q312A, E313A) was named as *Lrp8m* for genetically blocking sclerostin loop2‐ApoER2 interaction in the following structure‐function studies.

**ApoER2 peptide tool for pharmacologic blockade of sclerostin loop2‐ApoER2 interaction**. BLI analysis revealed that ApoER2‐LA7 (P290‐L326, PCRENEFQCGDGTCVLAIKRCNQERDCPDGSDEAGCL) bound to sclerostin loop2 (*K_d_* = 1.9 µM) (Figure S7B, Supporting Information). The interaction between ApoER2 and sclerostin loop2 was blocked by pretreatment with ApoER2‐LA7 (Figure 6F). After LPS induction, there were no significant differences in the mRNA expression levels of TNF‐α, MCP‐1, and IL‐10 in sclerostin‐knockout macrophages (RAW264.7) between with and without treatment of ApoER2‐LA7 (Figure S7C, Supporting Information). Accordingly, ApoER2‐LA7 was named ApoER2‐Pep (peptide tool) for pharmacologically blocking sclerostin loop2‐ApoER2 interaction in the following structure‐function studies.

***Sost* mutation tool for genetic blockade of sclerostin loop2‐ApoER2 interaction**. The interaction of ApoER2 to sclerostin mutein with a series of mutations in sclerostin loop2 residues (Table S3, Supporting Information) were analyzed by pull‐down assay. The data showed that the binding ability of ApoER2 to SOST‐S4 (I94A, G95A, R96A) and SOST‐S5 (V97A, K98A, W99A) was significantly weaker than that of ApoER2 to wild‐type sclerostin (Figure S6E, Supporting Information). I94, G95, R96, K98, W99 were the key residues within sclerostin for the interaction between sclerostin and ApoER2, which was validated in molecular docking by HDOCK (Figure S6F, Supporting Information). Accordingly, we developed *sost^loop2m^* (encoding SOST^loop2m^ with mutations at residues I94A, G95A, R96A, K98A, W99A). The interaction of wild‐type/mutated sclerostin (SOST/SOST^loop2m^) to ApoER2 was analyzed by co‐IP assay. No interaction between SOST^loop2m^ and ApoER2 was determined (Figure 6E). Hence, *sost^loop2m^* (encoding SOST^loop2m^) was used as *sost* mutation tool for genetically blocking sclerostin loop2‐ApoER2 interaction in the subsequent structure‐function studies.

### 2.5 Blockade of Sclerostin Loop2‐ApoER2 Interaction Attenuated Suppressive Effects of Sclerostin on Inflammatory Responses in Macrophages In Vitro

In *Lrp8m*‐mediated structure‐function studies, the macrophages (RAW 264.7) were transfected with *Lrp8* plasmids and *Lrp8m* plasmids, respectively. After validating the overexpression of ApoER2(m) (Figure S7D, Supporting Information), the mRNA expression level of TNF‐α, MCP‐1, and IL‐10 in macrophages (RAW264.7) was determined by RT‐PCR. The data showed that the suppressive effects of exogenous sclerostin on mRNA expression of TNF‐α and MCP‐1 in LPS‐induced macrophages were significantly attenuated upon *Lrp8m* mutation, as well as the promoting effect of sclerostin on mRNA expression of IL‐10 (Figure 6G). Consistently, the suppressive effects of exogenous sclerostin on protein expression/secretion of TNF‐α and MCP‐1 in LPS‐induced macrophages were significantly attenuated upon *Lrp8m* mutation, as well as the promoting effect of sclerostin on protein expression/secretion of IL‐10 (Figure 6I). It indicated that *Lrp8m*‐induced genetic blockade of sclerostin loop2‐ApoER2 interaction attenuated the suppressive effects of sclerostin on inflammatory responses in macrophages in vitro.

In ApoER2‐Pep‐mediated structure‐function studies, ApoER2 overexpressing macrophages were induced with LPS, and treated with either exogenous sclerostin (SOST) or SOST + ApoER2‐Pep, followed by determination of the mRNA expression level of TNF‐α, MCP‐1, and IL‐10. The data showed that the suppressive effects of exogenous sclerostin on mRNA expression of TNF‐α and MCP‐1 in LPS‐induced macrophages were significantly attenuated by pretreatment with ApoER2‐Pep in vitro, as well as the promoting effect of sclerostin on mRNA expression of IL‐10 (Figure 6H). Consistently, the suppressive effects of exogenous sclerostin on protein expression/secretion of TNF‐α and MCP‐1 in LPS‐induced macrophages were significantly attenuated by pretreatment with ApoER2‐Pep in vitro, as well as the promoting effect of sclerostin on protein expression/secretion of IL‐10 (Figure 6J). It suggested that ApoER2‐Pep‐induced pharmacologic blockade of sclerostin loop2‐ApoER2 interaction attenuated the suppressive effects of sclerostin on inflammatory responses in macrophages in vitro.

### 2.6 *Lrp8m* Aggravated Inflammatory Responses and Cardiovascular Events, While It Was Dramatically Attenuated Upon *Lrp8m/Mac‐Lrp8*

To determine the effects of macrophagic *Lrp8m* on cardiovascular events in vivo, we initially aimed to generate a macrophage‐conditional *Lrp8m* mouse model. However, technical limitations hindered its development. As an alternative approach, the *ApoE^−/−^.Lrp8m* mouse model was constructed by crossbreeding *ApoE^−/−^* mice with *Lrp8m* mice (systematic *Lrp8m* encoding ApoER2m‐P253A, T254A, G268A, W269A, R270A, N311A, Q312A, E313A) (Figures S8 and S9, Supporting Information). We also developed *sost^−/−^*.*ApoE^−/−^* mice by crossbreeding *ApoE^−/−^* mice with *sost^−/−^* mice to further validate the preventive effects of sclerostin on cardiovascular events (Figure S9, Supporting Information). There was no significant difference in the serum sclerostin level between *ApoE^−/−^.Lrp8m* mice and *ApoE^−/−^* mice, while no serum sclerostin was determined in *sost^−/−^*.*ApoE^−/−^* mice (Figure S10A). We detected protein expression of ApoER2 in the aorta from wild‐type mice (Figure S10B, Supporting Information). The protein levels of ApoER2 in aortas from *ApoE^−/−^* mouse models were slightly lower than those from wild‐type mice. The protein levels of ApoER2(m) in aortas from *sost^−/−^.ApoE^−/−^* mice and *ApoE^−/−^.Lrp8m* mice were similar (Figure S10C, Supporting Information). In pull‐down assay of primary macrophages from aortas in *ApoE^−/−^.Lrp8m* mice, no ApoER2(m) band was detected in His‐sclerostin loop2 (His‐loop2) group. It suggested that the interaction between sclerostin loop2 and ApoER2 was blocked in *ApoE^−/−^.Lrp8m* mice (Figure S10D, Supporting Information).

**Figure 7:**
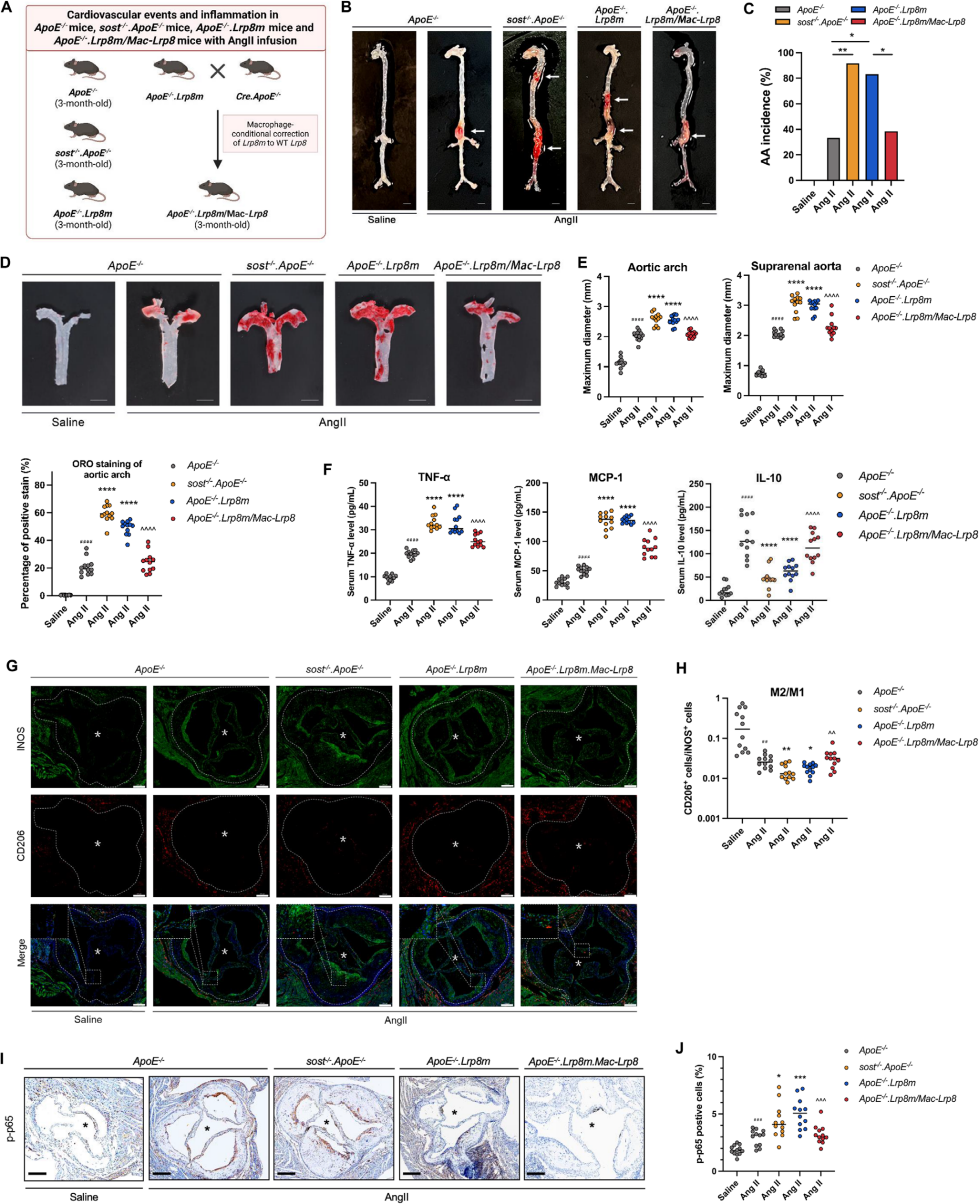
The aortic aneurysm, atherosclerosis, and inflammatory responses in *ApoE^−/−^* mice, *sost^−/−^.ApoE^−/−^* mice, *ApoE^−/−^.Lrp8m* and *ApoE^−/−^*.*Lrp8m/Mac‐Lrp8* mice, with AngII infusion. (A) The diagram of experimental design. (B) Representative images of aortas in *ApoE^−/−^* mice, *sost^−/−^.ApoE^−/−^* mice, *ApoE^−/−^.Lrp8m* and *ApoE^−/−^*.*Lrp8m/Mac‐Lrp8* mice with AngII infusion (scale bars, 4 mm). (C) Bar charts of the aortic aneurysm (AA) incidence. * *P < 0.05*, ** *P < 0. 01* for intergroup comparison by Fisher's exact test. (D) Representative images for *en face* Oil Red O staining of aortic arches (upper). Quantification of atherosclerotic plaque (lower). (E) *Ex vivo* measurement of the maximum diameters of aortic arches and suprarenal aortas. (F) The serum levels of inflammatory cytokine (TNF‐α), chemokine (MCP‐1), and anti‐inflammatory cytokine (IL‐10). (G) Representative immunofluorescence (IF) images for expression of iNOS (green) and CD206 (red) in paraffin sections of aortic roots (white dotted line, cell nucleus: blue). Scale bar: 200 µm (*lumen). (H) Quantification of the ratio of M2 anti‐inflammatory macrophage phenotype to M1 pro‐inflammatory macrophage phenotype in paraffin sections of aortic roots. (I) Representative Immunohistochemistry (IHC) images for phospho‐NF‐κB (p‐p65) in paraffin sections of aortic roots (black dotted line). Scale bar: 200 µm (*lumen). (J) Quantification of the ratio of phospho‐NF‐κB (p‐p65)‐positive cells to total cells in paraffin sections of aortic roots. Data were expressed as mean ± standard deviation. n = 12 per group. ^#^ *P < 0.05*, ^##^ *P < 0.01*, ^###^ *P < 0.001* and ^####^ *P < 0.0001* for a comparison versus *ApoE^−/−^* + saline group by unpaired t‐test. * *P < 0.05*, ** *P < 0. 01*, *** *P < 0.001* and **** P < 0.0001 for a comparison versus *ApoE^−/−^* + AngII by one‐way ANOVA with Tukey's post‐hoc test. ^*P < 0.05*, ^^*P < 0.01*, ^^^*P < 0.001* and ^^^^*P < 0.0001* for a comparison between *ApoE^−/−^*.*Lrp8m/Mac‐Lrp8* + AngII group and *ApoE^−/−^.Lrp8m* + AngII group by unpaired t‐test. All tests were two‐sided. Note: AngII: Angiotensin II; TNF‐α: tumor necrosis factor alpha; MCP‐1: monocyte chemoattractant protein‐1; IL‐10: interleukin‐10; iNOS: inducible nitric oxide synthase (pro‐inflammatory macrophages biomarker); CD206: cluster of differentiation 206, known as mannose receptor C‐type 1 (anti‐inflammatory macrophages biomarker).

Compared to *ApoE^−/−^* mice with AngII infusion, the aortic aneurysm (AA) incidence, the maximum *ex vivo* diameters of aortic arches and suprarenal aortas, the ratio of atherosclerotic plaque in aortic arches were significantly higher in *ApoE^−/−^.Lrp8m* mice and *sost^−/−^*.*ApoE^−/−^* mice, with AngII infusion. Moreover, higher serum levels of pro‐inflammatory cytokine (TNF‐α) and pro‐inflammatory chemokine (MCP‐1), lower serum levels of anti‐inflammatory cytokine (IL‐10), higher proportion of pro‐inflammatory macrophages (iNOS^+^), and higher ratio of phospho‐NF‐κB (p‐p65)‐positive cells in aortic roots were found in *ApoE^−/−^.Lrp8m* mice and *sost^−/−^*.*ApoE^−/−^* mice with AngII infusion, in comparison with *ApoE^−/−^* mice with AngII infusion (**Figure** **7**). It indicated that *Lrp8m* notably promoted NF‐κB phosphorylation and macrophage‐mediated inflammatory responses, and aggravated cardiovascular events in *ApoE^−/−^.Lrp8m* mice, which mimicked the phenotype of *sost^−/−^*.*ApoE^−/−^* mice.

Then, *ApoE^−/−^.Lrp8m* mice were further crossed with *Cre.ApoE^−/−^* mice to develop the *ApoE^−/−^.Lrp8m/Mac‐Lrp8* mice, in which *Lrp8* mutation was corrected to wild‐type *Lrp8* in macrophages (Figure 7A, Figures S8 and S9, Supporting Information). There was no significant difference in the serum sclerostin level among *ApoE^−/−^.Lrp8m/Mac‐Lrp8* mice, *ApoE^−/−^.Lrp8m* mice and *ApoE^−/−^* mice (Figure S10A, Supporting Information). The protein levels of ApoER2(m) in aorta from *ApoE^−/−^.Lrp8m/Mac‐Lrp8* mice and *ApoE^−/−^.Lrp8m* mice were similar (Figure S10C, Supporting Information). In pull‐down assay of primary macrophages from aortas in *ApoE^−/−^.Lrp8m/Mac‐Lrp8* mice, ApoER2(m) band was detected in His‐sclerostin loop2 (His‐loop2) group. It suggested that the interaction between sclerostin loop2 and ApoER2 in aortic macrophages was not blocked in *ApoE^−/−^.Lrp8m/Mac‐Lrp8* mice (Figure S10D, Supporting Information).

Compared to *ApoE^−/−^.Lrp8m* mice with AngII infusion, the AA incidence, the maximum *ex vivo* diameters of aortic arches and suprarenal aortas, the ratio of atherosclerotic plaque in aortic arches were significantly lower in *ApoE^−/−^.Lrp8m/Mac‐Lrp8* mice with AngII infusion. Moreover, significantly lower serum levels of pro‐inflammatory cytokine (TNF‐α) and pro‐inflammatory chemokine (MCP‐1), higher serum levels of anti‐inflammatory cytokine (IL‐10), significantly higher proportion of anti‐inflammatory macrophages (CD206^+^), and lower ratio of phospho‐NF‐κB (p‐p65)‐positive cells in aortic roots were determined in *ApoE^−/−^.Lrp8m/Mac‐Lrp8* mice with AngII infusion (Figure 7). We also determined the proportion of collagen fiber in atherosclerotic plaques at aortic roots of the aforementioned mice by Masson's trichrome staining. At the end of the experiments (AngII infusion for four weeks), we could not observe a difference in fibrotic repair of atherosclerotic lesions between *ApoE^−/−^*.*Lrp8m* mice and *ApoE^−/−^*.*Lrp8m/Mac‐Lrp8* mice (Figure S10E, Supporting Information). It suggested that macrophagic sclerostin loop2‐ApoER2 interaction could not be involved in fibrotic repair of atherosclerotic lesions.

Collectively, *Lrp8m* promoted NF‐κB phosphorylation and macrophage‐mediated inflammatory responses, aggravated cardiovascular events in *ApoE^−/−^*.*Lrp8m* mice, whilst these effects were notably attenuated upon macrophagic correction of *Lrp8m* to wild‐type *Lrp8* in *ApoE^−/−^*.*Lrp8m/Mac‐Lrp8* mice, indicating the critical role of macrophagic ApoER2 in suppressing inflammatory responses and preventing atherosclerosis and aortic aneurysm in *ApoE^−/−^* mice in vivo.

### 2.7 Genetically, Both *Lrp8m*‐Induced and *sost^loop2m^*‐Induced Blockade of Sclerostin Loop2‐ApoER2 Interaction Attenuated the Suppressive Effects of Sclerostin on AA, Atherosclerosis, and Inflammatory Responses In Vivo

**Figure 8:**
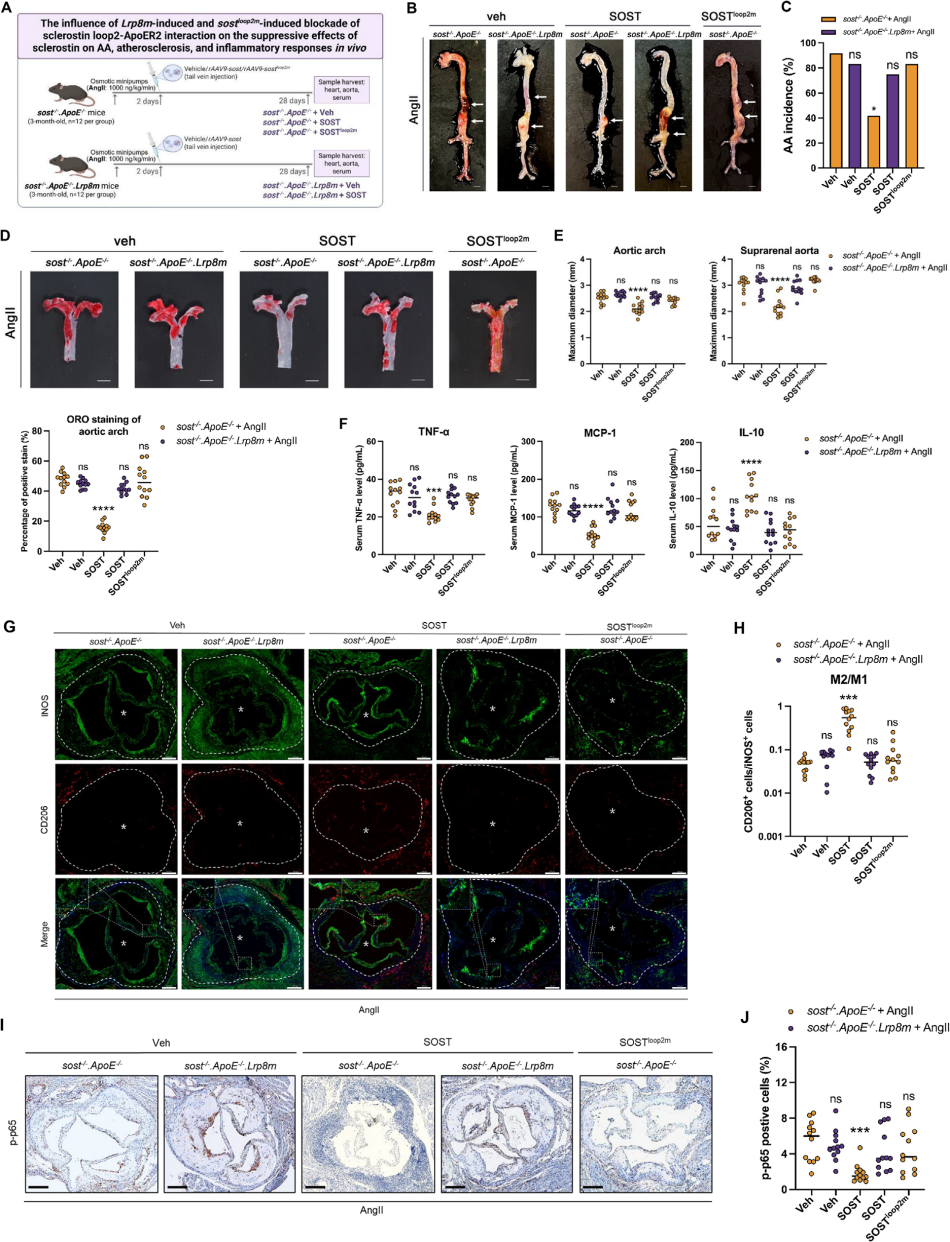
The aortic aneurysm, atherosclerosis, and inflammatory responses in *sost^−/−^*.*ApoE^−/−^* mice and *sost^−/−^*.*ApoE^−/−^.Lrp8m* mice, with and without *rAAV8‐*mediated re‐expression of SOST or SOST^loop2m^. (A) The diagram of experimental design. (B) Representative images of aortas in *sost^−/−^.ApoE^−/−^.Lrp8m* mice and *sost^−/−^.ApoE^−/−^* mice, with AngII infusion (scale bars, 2 mm). (C) Bar charts of the aortic aneurysm (AA) incidence. ^ns^ *P > 0.05*, * *P < 0.05* for a comparison versus Fisher's exact test. (D) Representative *en face* Oil Red O staining images of aortic arches (upper) and quantification of atherosclerotic plaque (lower). (E) *Ex vivo* measurement of the maximum diameters of aortic arches and suprarenal aortas. (F) The serum levels of pro‐inflammatory cytokine (TNF‐α), pro‐inflammatory chemokine (MCP‐1) and anti‐inflammatory cytokine (IL‐10). (G) Representative immunofluorescence (IF) images for expression of iNOS (green) and CD206 (red) in paraffin sections of aortic roots (white dotted line, cell nucleus: blue). Scale bar: 200 µm (*lumen). (H) Quantification of the ratio of M2 anti‐inflammatory macrophage phenotype to M1 pro‐inflammatory macrophage phenotype in paraffin sections of aortic roots. (I) Representative Immunohistochemistry (IHC) images for phospho‐NF‐κB (p‐p65) in paraffin sections of aortic roots (black dotted line). Scale bar: 200 µm (*lumen). (J) Quantification of the ratio of phospho‐NF‐κB (p‐p65)‐positive cells to total cells in paraffin sections of aortic roots. Data were expressed as mean ± standard deviation. n=12 per group. *^ns^P* > 0.05, * *P < 0.05*, ** *P < 0. 01*, *** *P < 0.001*, and **** P < 0.0001 for the comparison versus Veh controls by two‐way ANOVA with Tukey's post‐hoc test. All tests were two‐sided. Note: AngII: Angiotensin II; TNF‐α: tumor necrosis factor alpha; MCP‐1: monocyte chemoattractant protein‐1; IL‐10: interleukin‐10; iNOS: inducible nitric oxide synthase (pro‐inflammatory macrophages biomarker); CD206: cluster of differentiation 206, known as mannose receptor C‐type 1 (anti‐inflammatory macrophages biomarker).

To determine whether the aggravative effects of *Lrp8m* on cardiovascular events and inflammatory responses were caused by genetic blockade of sclerostin loop2‐ApoER2 interaction in vivo, the *sost^−/−^.ApoE^−/−^.Lrp8m* mouse model was constructed by crossbreeding *ApoE^−/−^.Lrp8m* mice with *sost^−/−^.ApoE^−/−^* mice for shielding the effects of endogenous sclerostin (Figures S8 and S9, Supporting Information). Then, the *rAAV8‐sost* (encoding SOST) was intravenously injected in *sost^−/−^.ApoE^−/−^* mice and *sost^−/−^.ApoE^−/−^.Lrp8m* mice, respectively, for re‐expression of SOST. The *rAAV8‐sost^loop2m^* (encoding SOST^loop2m^) was intravenously injected in *sost^−/−^.ApoE^−/−^* mice for re‐expression of SOST^loop2m^ (**Figures** **8**A, S11A, Supporting Information). Similar protein levels of ApoER2 were determined in aortas from both *sost^−/−^.ApoE^−/−^* mice and *sost^−/−^.ApoE^−/−^.Lrp8m* mice (Figure S11B, Supporting Information). After AngII infusion, both *sost^−/−^.ApoE^−/−^* mice and *sost^−/−^.ApoE^−/−^.Lrp8m* mice exhibited severe AA, atherosclerosis, and inflammatory responses (Figure 8). With re‐expression of SOST, *sost^−/−^.ApoE^−/−^* mice showed significantly lower AA incidence, lower maximum *ex vivo* diameters of aortic arches and suprarenal aortas, lower ratio of atherosclerotic plaque in aortic arches, lower serum levels of pro‐inflammatory cytokine (TNF‐α) and pro‐inflammatory chemokine (MCP‐1), higher serum levels of anti‐inflammatory cytokine (IL‐10), higher proportion of anti‐inflammatory macrophages (CD206^+^), lower ratio of phospho‐NF‐κB (p‐p65)‐positive cells in aortic roots. In contrast, there were no significant differences in the above parameters regarding inflammatory responses, atherosclerosis, and AA progression in *sost^−/−^.ApoE^−/−^* mice between with and without re‐expression of SOST^loop2m^ (Figure 8). Moreover, there were no significant differences in the above parameters in *sost^−/−^.ApoE^−/−^.Lrp8m* mice with and without re‐expression of SOST (Figure 8). Accordingly, both *sost^loop2m^* and *Lrp8m*‐induced blockade of sclerostin loop2‐ApoER2 interaction dramatically attenuated the suppressive effects of sclerostin on NF‐κB phosphorylation and inflammatory responses, reduced the promotive effects of sclerostin on aortic macrophage conversion to anti‐inflammatory phenotypes, inhibited the preventive effects of sclerostin on atherosclerosis and aortic aneurysm development in *ApoE^−/−^* mice in vivo.

### 2.8 Pharmacologically, ApoER2‐Pep‐Induced Blockade of Sclerostin Loop2‐ApoER2 Interaction Attenuated the Suppressive Effects of Sclerostin on AA, Atherosclerosis, and Inflammatory Responses In Vivo

To pharmacologically determine the role of sclerostin loop2‐ApoER2 interaction in the suppressive effects of sclerostin on cardiovascular events and inflammatory responses, ApoER2‐Pep was end‐protected by changing the front and back three amino acids from L‐configuration amino acids to D‐configuration amino acids to improve hydrolytic stability in vivo.^[^ ^27^, ^28^ ^]^ The in vivo administration dosage, interval, and duration of the modified ApoER2‐Pep were defined as 10 mg/kg/day for four weeks, based on the elimination half‐life (T_1/2_ = 9.3h) and small‐scale preliminary studies in vivo. Moreover, the ApoER2‐Pep was determined to have no effect on inflammatory responses in *sost^−/−^.ApoE^−/−^* mice with AngII infusion in vivo, in the absence of sclerostin (Figure S12, Supporting Information).

**Figure 9:**
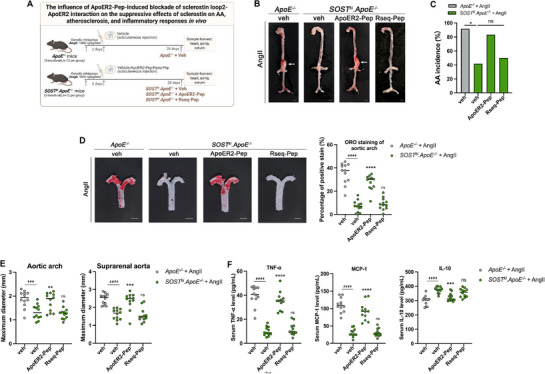
The effects of exogenous ApoER2‐Pep peptide tool on aortic aneurysm, atherosclerosis, and inflammatory responses in *SOST^ki^.ApoE^−/−^* mice with AngII infusion. (A) The diagram of experimental design. (B) Representative images of aortas in *SOST^ki^.ApoE^−/−^* mice, with AngII infusion (scale bars, 2 mm). (C) Bar charts of aortic aneurysm (AA) incidence. ^ns^ *P > 0.05*, * *P < 0.05* for intergroup comparison by Fisher's exact test. (D) Representative *en face* Oil Red O staining images of aortic arches (left) and quantification of atherosclerotic plaque (right). (E) *Ex vivo* measurement of maximum diameters of aortic arches and suprarenal aortas. (F) The serum levels of pro‐inflammatory cytokine (TNF‐α), pro‐inflammatory chemokine (MCP‐1), and anti‐inflammatory cytokine (IL‐10). Data were expressed as mean ± standard deviation. n = 12 per group. ^#^ *P < 0.05*, ^##^ *P < 0.01*, ^###^ *P < 0.001* and ^####^ *P < 0.0001* for a comparison between *ApoE^−/−^* + AngII + veh group and *SOST^ki^.ApoE^−/−^* + AngII + veh group by unpaired t‐test. ^ns^ *P > 0.05*, * *P < 0.05*, ** *P < 0. 01*, *** *P < 0.001* and **** P < 0.0001 for a comparison versus *SOST^ki^.ApoE^−/−^* + AngII + veh by one‐way ANOVA with Tukey's post‐hoc test. All tests were two‐sided. Note: AngII: Angiotensin II; TNF‐α: tumor necrosis factor alpha; MCP‐1: monocyte chemoattractant protein‐1; IL‐10: interleukin‐10; Rseq‐Pep: peptide with random sequence.

After subcutaneous administration of exogenous ApoER2‐Pep (10 mg/kg/day) or vehicle (veh) for four weeks, the parameters regarding AA, atherosclerosis, and inflammatory responses were determined in *SOST^ki^.ApoE^−/−^* mice with AngII infusion (**Figure** **9** **A)**. The data showed that the AA incidence, the maximum *ex vivo* diameters of aortic arches and suprarenal aortas, the ratio of atherosclerotic plaque in aortic arches, the serum levels of pro‐inflammatory cytokine (TNF‐α) and pro‐inflammatory chemokine (MCP‐1) were significantly higher, the serum levels of anti‐inflammatory cytokine (IL‐10) were significantly lower in the *SOST^ki^.ApoE^−/−^ +* ApoER2‐Pep group, when compared to those in *SOST^ki^.ApoE^−/−^ +* veh group and *SOST^ki^.ApoE^−/−^ +* Rseq‐Pep group (Rseq‐Pep: peptide with random sequence) (**Figure** **9**). It indicated that pharmacologic blockade of sclerostin loop2‐ApoER2 interaction attenuated the suppressive effects of endogenous sclerostin on inflammatory responses, diminished the preventive effects of endogenous sclerostin on atherosclerosis and aortic aneurysm in *SOST^ki^.ApoE^−/−^* mice in vivo.

## 3 Discussion

This study showed, for the first time, that the interaction between sclerostin loop2 and macrophagic ApoER2 was required by sclerostin to suppress inflammatory responses, atherosclerosis, and aortic aneurysm in *ApoE^−/−^* mice with AngII infusion.

**Regarding the role of sclerostin in the cardiovascular system**, our studies on inflammatory responses, atherosclerosis, and aortic aneurysm in *SOST^ki^.ApoE^−/−^* mice^[^ ^14^ ^]^ and *sost^−/−^*.*ApoE^−/−^* mice collectively indicated the protective action of sclerostin in cardiovascular system in vivo. Our published studies of antibody against sclerostin loop2 in *ApoE^−/−^* mice and GWAS analysis of the *SOST* variants in U.K. Biobank in this study further suggested the critical role of sclerostin loop2 in the cardiovascular protective action of sclerostin.

Combining our scRNA‐Seq analysis of aortas from *ApoE^−/−^* mice and *SOST^ki^.ApoE^−/−^* mice with our in vitro studies, it was notably found that the suppressive effects of sclerostin on atherosclerotic inflammatory responses were dependent on ApoER2 in macrophages, including the suppressive effects on NF‐κB nuclear translocation and phosphorylation, mRNA expression level of NF‐κB, as well as the promotive effects on macrophage conversion to anti‐inflammatory phenotypes. **Importantly, sclerostin loop2 was identified to physically bind to ApoER2 LA7 in macrophages**.

**Figure 10:**
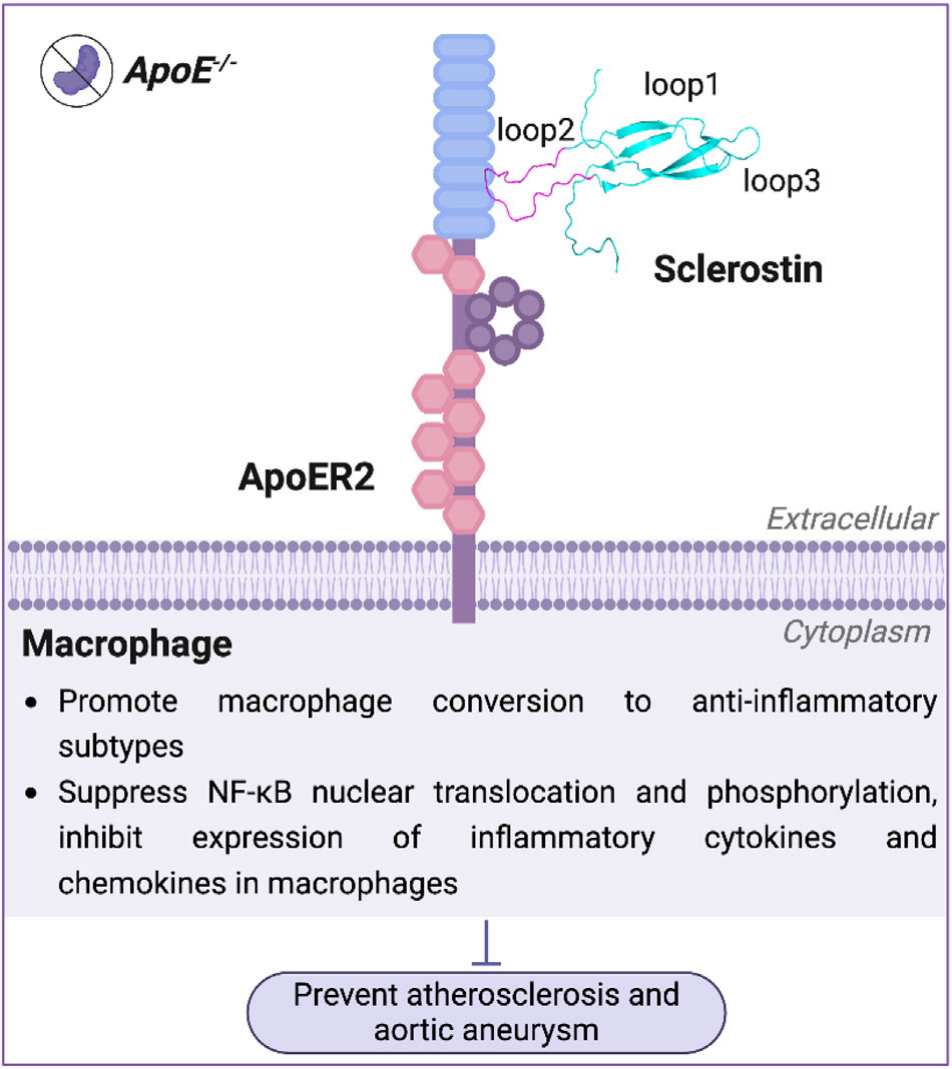
Schematic diagram showing our findings that sclerostin loop2‐ApoER2 interaction in macrophages was required by sclerostin to suppress NF‐κB nuclear translocation and phosphorylation, to promote macrophage conversion into anti‐inflammatory phenotypes in atherosclerotic aortas, as well as to prevent atherosclerosis and aortic aneurysm development in *ApoE^−/−^* mice.

**Regarding the role of sclerostin loop2‐ApoER2 interaction in the cardiovascular protective action of sclerostin**, AngII‐induced atherosclerosis/aortic aneurysm studies using either genetic or pharmacological approaches consistently indicated that macrophagic sclerostin loop2‐ApoER2 interaction was required by sclerostin to suppress NF‐κB nuclear translocation and phosphorylation, to promote macrophage conversion into anti‐inflammatory phenotypes in atherosclerotic lesions, as well as to prevent inflammatory responses, atherosclerosis and aortic aneurysm development in *ApoE^−/−^* mice (**Figure** **10**).

Severe cardiovascular events were found in both clinical trials (BRIDGE^[^ ^6^ ^]^ and ARCH^[^ ^5^ ^]^) and post‐marketing clinical applications (US‐FEAR^[^ ^8^ ^]^ and JADER^[^ ^7^, ^9^ ^]^) of the marketed therapeutic antibody against sclerostin loop2. The published data from the other groups and our group indicated that transgenic introduction of sclerostin in *ApoE^−/−^* mice inhibited AngII‐induced inflammatory responses, atherosclerosis, and aortic aneurysm.^[^ ^13^, ^14^ ^]^ Our data in this study further showed that *sost^−/−^*.*ApoE^−/−^* mice exhibited dramatically more severe inflammatory responses, atherosclerosis, and aortic aneurysm than *ApoE^−/−^* mice, with AngII infusion. It strongly indicated the protective role of sclerostin in the cardiovascular system of *ApoE^−/−^* mice.

Moreover, the key question of how sclerostin participates in protecting cardiovascular system was addressed in this study. Apolipoprotein E receptor 2 (ApoER2) was notably identified as a novel receptor of sclerostin in macrophages and mediated the suppressive effects of sclerostin on NF‐κB‐driven inflammatory responses, as well as the preventive effects of sclerostin on atherosclerosis and aortic aneurysm in *ApoE^−/−^* mice. ApoER2 was reported to mediate the effects of Apolipoprotein E (ApoE) on promoting macrophages conversion into anti‐inflammatory phenotypes and on inhibition of NF‐κB‐derived inflammatory responses including atherosclerotic plaque inflammation in vivo.^[^ ^16^, ^23^, ^24^, ^25^ ^]^ Here, in addition to ApoE, sclerostin emerged as a novel ligand of ApoER2 in macrophages for inhibition of inflammation, atherosclerosis, and aortic aneurysm in vivo. Sclerostin, interacting with ApoER2, played a compensatory protective role in cardiovascular system when ApoE is absent or mutated. It provided critical pre‐clinical evidence regarding the prediction of the cardiovascular risk populations (e.g.*, APOE* variants) for the marketed antibody against sclerostin loop2 during clinical application. It could facilitate developing a clinical guideline for precision medicine with the marketed sclerostin antibody, e.g., which patients might benefit from it, and which patients, such as *APOE* variants, might be at a high risk of cardiovascular events.

While the above findings provide valuable insights into the critical role of macrophagic sclerostin loop2‐ApoER2 interaction in suppressing NF‐κB‐driven inflammatory responses in aortic macrophages, the downstream molecular mechanisms of sclerostin loop2‐ApoER2 interaction remain incompletely elucidated. In subsequent studies, we will investigate how sclerostin loop2‐ApoER2 interaction inhibits NF‐κB nuclear translocation and phosphorylation in macrophages, as well as its regulatory effect on NF‐κB mRNA expression in macrophages. In addition, *Lrp8m* mutation and ApoER2‐Pep treatment significantly attenuated the suppressive effects of sclerostin on mRNA expression of TNF‐α (partial reversal) and MCP‐1 (complete reversal), as well as the promotive effects of sclerostin on mRNA expression of IL‐10 (complete reversal) in LPS‐induced macrophages in vitro. The partial reversal of TNF‐α mRNA expression level suggests other unclear compensatory mechanisms in the anti‐inflammatory effects of sclerostin/ApoER2. Discovering the other inflammatory pathways and potential alternative receptors for sclerostin (beyond ApoER2) that might contribute to atherosclerotic progression would significantly extend our understanding of the complex inflammatory network in atherosclerosis, which we plan to pursue in subsequent studies.

In terms of structural biology, sclerostin loop2 was reported to bind to YWTD repeats (β‐propeller) within low‐density lipoprotein receptor (LDLR)‐related protein 5 and 6 (LRP5/6) of osteoblasts, thereby participating in the antagonistic effect of sclerostin on osteoblastic Wnt/β‐catenin signal pathway and bone formation.^[^ ^2^ ^]^ In this study, we notably identified ApoER2 (also known as LRP8), another transmembrane receptor belonging to the LDLR family,^[^ ^16^ ^]^ as a novel receptor of sclerostin in macrophages. Mechanistically, LDLR type A 7 (LA7) in the known ligand‐binding LA repeats of ApoER2^[^ ^29^ ^]^ was found to mediate the interaction between sclerostin loop2 and macrophagic ApoER2, which was required by sclerostin to inhibit inflammation, atherosclerosis, and aortic aneurysm. ApoER2 was known as a neuronal receptor for reelin during brain development.^[^ ^30^ ^]^ However, reelin‐DAB1 cascade^[^ ^31^ ^]^ was not found in our aortic macrophages from scRNA‐Seq (KEGG pathways enrichment) analysis in this study. It suggested that ApoER2 could mediate tissue/cell‐specific functions with distinct ligand: ApoER2 mediated neuroinflammation promotive function via reelin‐ApoER2 LA1 interaction in endothelial cells^[^ ^30^, ^32^ ^]^ of the central nervous system (CNS), whereas ApoER2 mediated cardiovascular protective function^[^ ^17^, ^21^, ^33^ ^]^ via macrophagic sclerostin loop2‐ApoER2 LA7 interaction in the cardiovascular system.

Translationally, targeting sclerostin while preserving macrophagic sclerostin loop2‐ApoER2 interaction would offer the next generation of precise sclerostin inhibition strategy with no safety concern in the cardiovascular system, for promoting bone formation.

## 4 Experimental Section

### Experimental Design

**Study 1: ScRNA‐seq analysis of aortic macrophages in *SOST^ki^.ApoE^−^^/^^−^* mice and *ApoE^−^^/^^−^* mice**

Three‐month‐old *ApoE^−/−^* mice and 3‐month‐old *SOST^ki^.ApoE^−/−^* mice were infused with AngII for four weeks (n = 6).^[^ ^14^ ^]^ Aortic tissues were enzymatically digested into single‐cell suspensions for scRNA‐seq, followed by rigorous quality control to exclude low‐viability cells based on metrics such as mitochondrial RNA content, gene counts, and total RNA abundance. After normalization and variance stabilization, dimensionality reduction (PCA) and Leiden clustering identified 13 major cell populations within the aortic niche, including macrophages, endothelial cells, and smooth muscle cells. Macrophages were further sub‐clustered into five subtypes (M1‐like 1, M1‐like 2, M2‐like, Res‐like 1, Res‐like 2) using marker genes (*S100a8, S100a4, Mrc1, Trem2, Rpl37*), with subtype‐specific transcriptional profiles visualized via ridge plots, violin plots, and heatmaps. Comparative analysis of macrophage subtype proportions between genotypes employed UMAP‐based density mapping, while trajectory inference (PAGA Slingshot) mapped differentiation dynamics to infer lineage plasticity. Differential gene expression (DGE) analysis between *SOST^ki^. ApoE^−/−^* and *ApoE^−/−^* macrophages identified sclerostin‐dependent transcriptional changes, followed by KEGG pathway enrichment to link these genes to inflammatory signaling cascades. This integrative approach‐spanning cell clustering, lineage trajectory reconstruction, and pathway enrichment analysis, systematically dissected how sclerostin regulated macrophage polarization and inflammatory responses, providing a mechanistic framework for its preventive role in atherosclerosis and aortic aneurysm.

**Study 2: Determination of whether the suppressive effects of sclerostin on inflammatory responses in macrophages were dependent on ApoER2 in vitro**

To determine whether the suppressive effects of sclerostin on inflammatory responses in macrophages were dependent on ApoER2 in vitro, PMA (100 ng/mL, 48 h)‐induced human differentiated macrophages (THP‐1 cells, Procell Life Science & Technology, CL‐0233) and mouse macrophages (RAW 264.7 cells, ATCC, TIB‐71) were utilized. Human differentiated macrophages or mouse macrophages were divided into 9 groups (n = 3 per group), respectively, including (1) siNC, (2) siNC + LPS, (3) siNC + LPS + SOST 500 nM or 250 nM, (4) siNC + LPS + SOST 1 µM or 500 nM, (5) siNC + LPS + SOST 2 µM or 1 µM, (6) si*Lrp8* + LPS + SOST 500 nM or 250 nM, (7) si*Lrp8* + LPS + SOST 1µM or 500 nM, (8) si*Lrp8* + LPS + SOST 2 µM or 1 µM, (9) si*Lrp8* + LPS. Cells in groups (6‐9) were transfected with siRNA targeting human/mouse *Lrp8* (human si*Lrp8*: Santa Cruz Biotechnology sc‐40097, mouse si*Lrp8*: Santa Cruz Biotechnology sc‐40098), while cells in groups (1‐5) were transfected with empty vector as control (siNC). 24 h later, cells in group (3)/(6), (4)/(7), and (5)/(8) were treated with recombinant human or mouse sclerostin for 3 h at concentration of 500 nM or 250 nM, 1 µM or 500 nM, as well as 2 µM or 1 µM, respectively, while cells in groups (1), (2), and (9) were treated with vehicle. Then, cells in groups (2‐9) were treated with LPS (100 ng/mL or 1 µg/mL) for 18 h. All groups of cell lysates were collected for determining the mRNA expression levels of TNF‐α, MCP‐1, and IL‐10 (human or mouse, respectively) by RT‐PCR.^[^ ^34^ ^]^ Cell mediums were collected for determining the protein expression/secretion levels of TNF‐α, MCP‐1, and IL‐10 (human or mouse, respectively) by ELISA.^[^ ^34^ ^]^

Further, we tracked the dynamic changes of inflammatory responses in mouse RAW264.7 macrophages (with *Lrp8* silencing for shielding the influence of endogenous ApoER2). The RAW264.7 macrophages (si*Lrp8*) were transfected with plasmids encoding ApoER2 (*Lrp8*) or vector, followed by treatment with recombinant mouse sclerostin and induction with LPS for varying durations. In detail, RAW 264.7 macrophages (si*Lrp8*) were divided into 7 groups (n = 3 per group), including (1) Control, (2) LPS, (3) LPS + SOST, (4) *Lrp8* + LPS + SOST 250 nM, (5) *Lrp8* + LPS + SOST 500 nM, (6) *Lrp8* + LPS + SOST 1 µM, and (7) *Lrp8* + LPS. Cells in group (4–7) were transfected with plasmids encoding mouse ApoER2 (*Lrp8*), while cells in group (1–3) were transfected with vector. Twenty four hours later, cells in groups (3), (4), (5), and (6) were treated with recombinant mouse sclerostin at concentration of 1 µM, 250 nM, 500 nM, and 1 µM for 3 h, respectively, while cells in groups (1), (2), and (7) were treated with vehicle.^[^ ^35^ ^]^ Then, cells in group (2‐7) were treated with LPS (1 µg/mL) for 18 h. All groups of cell lysates were collected for determining the mRNA expression levels of mouse TNF‐α, mouse MCP‐1, and mouse IL‐10 by RT‐PCR. Given the significant efficacy of recombinant mouse sclerostin at a concentration of 1 µM in mouse macrophages, 1 µM was selected for the subsequent studies.

Then, the protein expression/secretion levels of pro‐inflammatory/anti‐inflammatory cytokines and chemokines, macrophage conversion, NF‐κB subcellular localization, phosphorylation, and expression were determined. In detail, RAW 264.7 cells (si*Lrp8*) were divided into 5 groups (n = 3 per group), including (1) Control, (2) LPS, (3) LPS + SOST, (4) *Lrp8* + LPS + SOST, and (5) *Lrp8* + LPS. RAW 264.7 cells in groups (4) and (5) were transfected with plasmids encoding mouse ApoER2 (*Lrp8*), while cells in group (1‐3) were transfected with vector. Twenty four hours later, cells in groups (3) and (4) were treated with recombinant mouse sclerostin (SOST, 1 µM) for 3 h, while cells in groups (1), (2), and (5) were treated with vehicle.^[^ ^35^ ^]^ Then, cells in groups (2), (3), (4), and (5) were treated with LPS (1 µg/mL) for 12 and 18 h. Cell mediums were collected for determining the protein expression/secretion levels of mouse TNF‐α, mouse MCP‐1, and mouse IL‐10 by ELISA.^[^ ^34^ ^]^ The CD206^+^ macrophages and CD11b^+^ macrophages were determined by flow cytometry.^[^ ^36^ ^]^

Additionally, for the determination of NF‐κB (p65) nuclear translocation, macrophages in groups (3) and (4) were treated with recombinant mouse sclerostin (SOST, 1 µM) overnight, then macrophages in groups (2), (3), (4), and (5) were treated with LPS (1 µg/mL) for 15 and 30 min. The NF‐κB (p65) nuclear translocation in macrophages was examined under microscope, after LPS induction for 15 min.^[^ ^37^ ^]^ The protein level of NF‐κB (p65) in nuclear and cytoplasm was determined by western blot assay, after LPS induction for 30 min.^[^ ^38^ ^]^

Subsequently, for the determination of NF‐κB phosphorylation, macrophages in groups (3) and (4) were treated with recombinant mouse sclerostin (SOST, 1 µM) overnight, then macrophages in groups (2), (3), (4), and (5) were treated with LPS (1 µg/mL) for 6 h. The protein level of phospho‐NF‐κB (p‐p65) was determined by western blot assay, followed by calculating the proportion of p‐p65/total p65 in cells.^[^ ^39^ ^]^ At the same time, the mRNA expression level of NF‐κB (p65) was determined by RT‐PCR.^[^ ^40^ ^]^

**Study 3: GWAS analysis of sclerostin variants in UK Biobank**

Genome‐wide association study (GWAS) analysis was performed to investigate the association between sclerostin variants and cardiovascular abnormalities.^[^ ^41^ ^]^ Genetic and phenotypic data from the UK Biobank (UKB) were utilized for this analysis. PLINK (v1.90b6.21) was utilized for quality control, filtering, and association analysis.^[^ ^42^ ^]^ For sample QC, individuals with high missing genotype rates (>5%), sex discrepancies, abnormal heterozygosity, or close relatedness (kinship coefficient >0.125) were excluded. Variant QC filtered out SNPs with a missing call rate >5%, minor allele frequency (MAF) <1%, or deviation from Hardy‐Weinberg equilibrium (HWE *p*‐value <1e‐6). Association analyses employed logistic regression for binary traits (e.g., cardiac dysrhythmias, precordial pain, peripheral vascular disease) and linear regression for continuous traits, adjusted for age, sex, and the first 10 genetic principal components (PCs) to control for population stratification. A genome‐wide significance threshold of p <5e‐8 was applied. Manhattan plots highlighted genome‐wide p‐value distributions, emphasizing the SOST locus on chromosome 17, while phenograms visualized relationships between loop2‐specific SOST variants and cardiovascular abnormalities.^[^ ^43^ ^]^

**Study 4: Interaction analysis between sclerostin and ApoER2**

The interaction of full‐length (FL)/truncated ApoER2 with full‐length (FL) /truncated sclerostin in macrophages (RAW264.7) were determined by pull‐down assay and Co‐IP assay. The binding affinity of ApoER2 to FL sclerostin and sclerostin loop2 were determined by biolayer interferometry (BLI) analysis. Moreover, the binding of sclerostin to ApoER2 on macrophages (RAW264.7) was examined by confocal microscopy.

The interaction residues within ApoER2 to sclerostin loop2 were determined in combination with pull‐down assay and BLI analysis. Based on the interaction residues within ApoER2 to sclerostin loop2, a series of *Lrp8* mutations were developed. Based on the interaction residues within sclerostin loop2 to ApoER2, a series of *sost* mutations were developed. After identifying the mutation that blocked sclerostin loop2‐ApoER2 interaction, but did not inherently alter inflammatory pathways, *Lrp8* mutation tool (*Lrp8m*), ApoER2 peptide tool (ApoER2‐Pep), and *sost* mutation tool (*sost^loop2m^*) were designed to block sclerostin loop2‐ApoER2 interaction.

**Study 5: Role of sclerostin loop2‐ApoER2 interaction in suppressive effects of sclerostin on inflammatory responses in macrophages in vitro**

In *Lrp8m*‐mediated structure‐function studies in vitro, RAW 264.7 cells were divided into 4 groups (n = 3 per group), including (1) Control, (2) LPS, (3) *Lrp8* + LPS + SOST, and (4) *Lrp8m* + LPS + SOST. RAW 264.7 macrophages in groups (3) and (4) were transfected with *Lrp8* plasmids and *Lrp8m* plasmids, respectively, while cells in groups (1) and (2) were kept untreated. Twenty four hours later, cells in groups (3) & (4) were treated with recombinant sclerostin (SOST, 1µM) for 3 h. Then, cells in groups (2), (3), and (4) were treated with LPS (1µg/mL) for 18 h. All groups of cells were collected for determining the mRNA expression levels of TNF‐α, MCP‐1, and IL‐10 by RT‐PCR. Cell mediums were collected for determining the protein expression/secretion levels of mouse TNF‐α, mouse MCP‐1, and mouse IL‐10 by ELISA.

In ApoER2‐Pep‐mediated structure‐function studies in vitro, RAW 264.7 cells were divided into 4 groups (n = 3 per group), including (1) Control, (2) LPS, (3) *Lrp8* + LPS + SOST, and (4) *Lrp8* + LPS + SOST + ApoER2‐Pep. Cells in groups (3) and (4) were transfected with *Lrp8* plasmids encoding ApoER2, while cells in groups (1) and (2) were kept untreated. 24 h later, cells in group (3) were treated with recombinant sclerostin (SOST, 1 µM), while cells in group (4) were treated with recombinant sclerostin (1 µM) and ApoER2‐Pep (2 µM) for 3 h. Then, cells in groups (2), (3), and (4) were treated with LPS (1 µg/mL) for 18 h. All groups of cells were collected for determining the mRNA expression levels of TNF‐α, MCP‐1, and IL‐10 by RT‐PCR. Cell mediums were collected for determining the protein expression/secretion levels of mouse TNF‐α, mouse MCP‐1, and mouse IL‐10 by ELISA.

**Study 6: Role of macrophagic *Lrp8m* in cardiovascular events in vivo**

To further determine the role of macrophagic sclerostin loop2‐ApoER2 interaction in the suppressive effects of sclerostin on cardiovascular events in vivo, we initially aimed to generate macrophage‐conditional *Lrp8m* mouse model. However, technical limitations hindered its development. As an alternative approach, we established a systemic *Lrp8m* mouse model and crossed it with *ApoE^−/−^* mice to generate the *ApoE^−/−^.Lrp8m* mice. Then, *ApoE^−/−^.Lrp8m* mice were further crossed with *Cre.ApoE^−/−^* mice, yielding the *ApoE^−/−^.Lrp8m/Mac‐Lrp8* mouse model, in which *Lrp8* mutation in macrophages were corrected to wild‐type *Lrp8*. Moreover, *sost^−/−^*.*ApoE^−/−^* mice were constructed by crossbreeding *ApoE^−/−^* mice with *sost^−/−^* mice. Serum sclerostin levels in the above mouse models were determined by ELISA. The protein level of ApoER2(m) in aorta from *sost^−/−^.ApoE^−/−^* mice, *ApoE^−/−^.Lrp8m* mice and *ApoE^−/−^*.*Lrp8m/Mac‐Lrp8* mice were determined by Western blot. The primary macrophages in aorta were extracted from *ApoE^−/−^* mice, *ApoE^−/−^.Lrp8m* mice and *ApoE^−/−^.Lrp8m/Mac‐Lrp8* mice, followed by determining the interaction of sclerostin loop2 to ApoER2m/ApoER2 in the primary macrophage lysates by pull‐down assay.

The animals were grouped randomly and blindly. The animals in poor body condition were excluded. Considering the potential inhibitory effect of estrogen on atherosclerosis in female mice,^[^ ^44^ ^]^ 3‐month‐old male *ApoE^−/−^* mice were randomly divided into two groups and infused with AngII or saline, respectively, for four weeks (n = 12 per group). The *sost ^−/−^*.*ApoE^−/−^* mice*, ApoE^−/−^.Lrp8m* mice and *ApoE^−/−^.Lrp8m/Mac‐Lrp8* mice were infused with AngII for four weeks (n = 12 per group). Then, all mice were euthanized. Serum was collected for the determination of the levels of pro‐inflammatory cytokines and chemokines, as well as anti‐inflammatory cytokines.^[^ ^15^, ^45^ ^]^ The aorta with or without aneurysm formation was defined, and the incidence of AA was calculated.^[^ ^45^ ^]^ Aortas were collected for the determination of the maximum diameters of aortic arch and suprarenal aorta, and the ratio of atherosclerotic plaque in aortic arch. Hearts were collected. Cross cryo‐sections from aortic roots were obtained for analysis of collagen fiber area (% area),^[^ ^46^ ^]^ followed by immunofluorescence (IF) analysis of the proportion of the CD206^+^ anti‐inflammatory M2 macrophages to the iNOS^+^ pro‐inflammatory M1 macrophages (M2/M1) in atherosclerotic lesions.^[^ ^47^ ^]^ Paraffin sections of aortic roots were obtained for immunohistochemistry (IHC) analysis of the proportion of phospho‐NF‐κB (p‐p65) in atherosclerotic lesions.^[^ ^15^, ^48^ ^]^

**Study 7: Determination of whether *Lrp8m*‐induced and *sost^loop2m^*‐induced blockade of sclerostin loop2‐ApoER2 interaction aggravated cardiovascular events in vivo**

The *sost^−/−^.ApoE^−/−^.Lrp8m* mouse model was constructed by crossbreeding *ApoE^−/−^.Lrp8m* mice with *sost^−/−^.ApoE^−/−^* mice for shielding the effects of endogenous sclerostin. The animals were grouped randomly and blindly. The animals in poor body condition were excluded. Then, the *rAAV8‐sost* (encoding SOST) was intravenously injected in *sost^−/−^.ApoE^−/−^* mice and *sost^−/−^.ApoE^−/−^.Lrp8m* mice, respectively, for re‐expression of SOST (n = 12 per group). The *rAAV8‐sost^loop2m^* (encoding SOST^loop2m^) was intravenously injected in *sost^−/−^.ApoE^−/−^* mice for re‐expression of SOST^loop2m[^ ^13^ ^]^ (n = 12 per group). The parameters indicating inflammatory responses, atherosclerosis, and AA development were determined and compared between *sost ^−/−^*.*ApoE^−/−^.Lrp8m* mice and *sost ^−/−^*.*ApoE^−/−^* mice, after AngII infusion. Serum was collected, aortas and hearts were harvested for analysis as described in study 6.^[^ ^15^, ^45^, ^47^ ^]^

**Study 8: Pharmacologically validating the role of sclerostin loop2‐ApoER2 interaction in the preventive effects of sclerostin on cardiovascular events in vivo**.

To pharmacologically determine the role of sclerostin loop2‐ApoER2 interaction in the preventive effects of sclerostin on cardiovascular events, the first and last three amino acids of our developed ApoER2‐Pep (P290‐L326, PCRENEFQCGDGTCVLAIKRCNQERDCPDGSDEAGCL) were changed from L‐type to D‐type amino acids (end‐protected) to improve the hydrolytic stability in vivo.^[^ ^27^, ^28^ ^]^ The administration dosage, interval, and duration of the modified ApoER2‐Pep were defined as 10 mg/kg/day for four weeks, based on the elimination half‐life (T1/2 = 9.3 h) and small‐scale preliminary studies in vivo. It was determined that ApoER2‐Pep had no effect on inflammatory responses in *ApoE^−/−^.sost^−/−^* mice, in the absence of sclerostin. Then, the parameters regarding inflammatory responses, atherosclerosis, and AA development were determined and compared between *SOST^ki^.ApoE^−/−^* mice with and without subcutaneous administration of exogenous ApoER2‐Pep, after AngII infusion (n = 12 per group). Peptide with random sequence (Rseq‐Pep, 36 amino acids) was used as control (n = 12 per group). Serum was collected, aortas and hearts were harvested for analysis as described in study 6.^[^ ^15^, ^45^, ^47^ ^]^

### Evaluation protocols—Single‐cell RNA sequencing

Single‐cell RNA sequencing was performed using the 10x Genomics Chromium Single Cell 3′ v3 platform.^[^ ^49^ ^]^ Briefly, single‐cell suspensions were loaded onto a Chromium controller. Single cells were encapsulated into nanoliter‐scale Gel Beads in Emulsion (GEMs). cDNA libraries were generated from GEMs according to the manufacturer's protocol and subsequently sequenced on an Illumina NovaSeq 6000 platform.

### Quality Control and Preprocessing

Sequencing data were processed using the Cell Ranger pipeline (v4.0.0) from 10x Genomics for initial read alignment and feature counting. STAR (v2.7.3a) was used for read alignment.^[^ ^50^ ^]^ FeatureCounts (v2.0.1) was employed for quantification of gene expression.^[^ ^51^ ^]^ The data were subsequently analyzed using FASTQC (v0.11.9) for quality assessment and Trimmomatic (v0.39) for adapter trimming, ensuring high‐quality reads for downstream analysis.^[^ ^52^, ^53^ ^]^ Additionally, MultiQC (v1.9) was used for consolidated quality control reporting.^[^ ^54^ ^]^ Raw sequencing reads were aligned to the mm10 mouse reference genome using STAR (v2.7.3a). Cell barcodes and unique molecular identifiers (UMIs) were used to generate gene expression matrices with FeatureCounts. Cells with detected genes, which were fewer than 1000, more than 6000, or high mitochondrial RNA content (≥20%), were excluded from downstream analysis to ensure high data quality and avoid doublets or stressed cells. The resulting gene expression matrices were analyzed using Python's Scanpy library (v1.8.1) for filtering, normalization, and clustering.^[^ ^55^ ^]^

### Identification of Highly Variable Genes and Dimensionality Reduction

Highly variable genes were identified using Scanpy's highly_variable_genes function with default parameters. These genes were used for principal component analysis (PCA), and the top 10 principal components (PCs) were selected based on the variance ratio analysis, capturing most of the biologically relevant variation in the dataset.^[^ ^56^ ^]^ UMAP was used for dimensionality reduction, and Leiden clustering was performed using Scanpy to identify distinct cell clusters.^[^ ^57^ ^]^ The clusters were manually annotated based on canonical marker genes.

### Cell Type Annotation and Analysis of Macrophage Subtypes

Cell types were annotated by cross‐referencing cluster‐specific gene expression profiles with known canonical markers. Macrophage subtypes were further classified based on their transcriptional profiles, with particular attention to pro‐inflammatory (M1‐like) and anti‐inflammatory (M2‐like) subtypes. Violin and dot plots were generated using Scanpy to visualize the expression of key marker genes across clusters and subtypes, providing insights into the functional diversity of macrophage populations.^[^ ^55^ ^]^

### Pseudotime Analysis

Pseudotime analysis was conducted using partition‐based graph abstraction (PAGA) in Scanpy to infer lineage trajectories.^[^ ^58^ ^]^ The combined dataset of *ApoE^−/−^* and *SOST^ki^.ApoE^−/−^* macrophages were used to determine differentiation pathways, with a focus on distinguishing pro‐inflammatory and anti‐inflammatory subtypes. PAGA graphs were visualized to highlight key differentiation paths across different conditions.

### Differential Gene Expression And Pathway Enrichment Analysis

Differential gene expression (DGE) analysis was performed using Scanpy's rank_genes_groups function, comparing macrophage subtypes between *ApoE^−/−^* and *SOST^ki^.ApoE^−/−^* mice.^[^ ^55^ ^]^ Genes with a q‐value < 0.05 were considered significantly differentially expressed. KEGG pathway enrichment analysis was conducted on the differentially expressed genes using the gseapy library (v0.10.4), identifying pathways enriched in pro‐inflammatory or anti‐inflammatory macrophage populations.^[^ ^59^, ^60^ ^]^

### Biolayer Interferometry Analysis

Biolayer interferometry (BLI) analysis was performed on the Biomolecular Interaction Analyzer (ForteBio, Octet RED96e). To determine the binding affinity between ApoER2 and sclerostin, the ApoER2 protein (R&D, 3520‐AR) was biotinylated using 10 mM EZ‐Link Sulfo‐NHS‐LC‐LC‐Biotin (Thermo Scientific, 21 343) in a molar ratio of 1:20 for 2 h at 4 °C. The Slide‐A‐Lyzer Dialysis Cassettes (Thermo Scientific, 66 382) were used for excess biotin removal.^[^ ^61^ ^]^ Anti‐streptavidin (SA) tray biosensors (Sartorius, 18‐5019) were used to load biotin‐ApoER2 proteins. Biosensors were pre‐wet for 10 min using 200 µL PBS containing 0.02% Tween 20 (v/v) in a 96‐well plate. To determine the binding affinity between sclerostin loop2 and ApoER2‐LA7, anti‐penta‐HIS (HIS1K) biosensors (Sartorius, 18‐5102) were used to load His‐sclerostin loop2, the sclerostin loop2‐loaded biosensors were immersed in the ApoER2‐LA7 with different concentrations. To determine the binding affinity between ApoER2 and sclerostin loop2 with and without ApoER2‐LA7 (named as ApoER2‐Pep), anti‐HIS1K biosensors were used to load His‐ApoER2, the ApoER2‐loaded biosensors were immersed in the sclerostin loop2 samples with and without pretreatment of ApoER2‐Pep with different concentrations. Raw data were analyzed using Data Analysis Software (ForteBio, v8.1). All K_d_ values were calculated after deducting the non‐specific binding between the biosensors and the samples.^[^ ^62^, ^63^ ^]^

### Molecular Docking

Molecular docking analysis was conducted to predict the interaction between sclerostin and ApoER2. HDOCK was used to perform the docking simulations.^[^ ^64^ ^]^ The protein structures of human sclerostin and human ApoER2 were obtained from the Protein Data Bank (PDB).^[^ ^65^ ^]^ HDOCK was used for both protein‐protein and protein‐peptide docking to identify potential interaction residues and binding affinities. The resulting docking models were evaluated based on binding energy scores and visual inspection to identify the key residues involved in the interaction. Visualization of the docking results was performed using PyMOL (v2.5), allowing detailed examination of binding interfaces and critical residues contributing to the sclerostin‐ApoER2 interaction.^[^ ^66^ ^]^

### Pull‐Down Assay

Cell lysates were collected using RIPA Lysis and Extraction Buffer (Thermo Scientific, 89 901) supplemented with Protease Inhibitor Cocktail (CST, 5871) and centrifuged at 4000 rpm for 30 min at 4 °C, and replaced with binding buffer (PBS buffer, 20 mM imidazole, pH 8.0). After pretreatment with binding buffer three times, 20 µL Ni‐NTA Magnetic Agarose Beads were incubated with 6 µg His‐SOST and 6 µg His‐loop3 peptide, respectively, in binding buffer for 2 h at 4 °C with gentle rotation. The beads carrying His‐SOST and His‐loop3 peptide were then incubated with 500 µL cell lysate supernatant at 4 °C overnight with gentle rotation. To eliminate the influence of non‐specific binding between beads and cell lysate, 20 µL beads and 500 µL cell lysate were incubated under the same conditions. After incubation, the beads were washed 3 times with 500 µL wash buffer (PBS buffer, 20 mM imidazole, 0.005% Tween 20, pH 8.0), and finally eluted with elution buffer (PBS buffer, 250 mM imidazole, 0.005% Tween 20, pH 8.0).^[^ ^45^ ^]^

### Western Blot

The eluted liquid from pull‐down assay was then mixed with SDS‐PAGE loading buffer and boiled for 10 min at 95 °C to make samples. The denatured samples were electrophoresed on 10% Bis‐Tris gels. Separated proteins were transferred to PVDF membranes (Thermo Scientific, 88 518) with a constant 300 mA for 2 h, and the membranes were washed in Tris‐buffered saline with 0.1% Tween 20 (TBST) for three times. Subsequently, the membranes were blocked with 5% dry skimmed milk or 3% BSA (for phosphorylated p65) at room temperature for 1 h. Blots were then probed with anti‐FLAG antibody (Abcam, ab125243, 1:2000), anti‐His antibody (Abcam, ab245114, 1:1000), phospho‐NF‐κB p65 antibody (CST, 3033, 1:1000) and NF‐κB p65 antibody (CST, 8242, 1:1000), respectively, overnight at 4 °C. After washing with TBST (3 times, 5 min), the blots were incubated with corresponding HRP‐linked anti‐mouse IgG (CST, #7076, 1:2000) or HRP‐linked anti‐rabbit IgG (Bio‐Rad, STAR124P, 1:5000) for 1 h at room temperature. The ECL solution (BIO‐RAD, 1 705 061) was used to visualize detected proteins. Each Western blot assay was repeated three times.^[^ ^39^ ^]^

### Quantitative RT‐PCR

Total RNA was extracted from cells following the protocols of Trizol reagent (Invitrogen, 15 596 026). Reverse‐transcription was performed to obtain cDNA using HiScript III RT SuperMix kit (Vazyme, R323), and RT‐PCR was accomplished using a HiScript III RT SuperMix kit (Vazyme, R323) in a QuantStudio 7 PRO System (Applied Biosystems). The relative gene expression levels were calculated using the 2^−ΔΔCt^ method with GAPDH as the reference gene.^[^ ^40^ ^]^ The primers were as follows: mouse GAPDH (F: AGGTCGGTGTGAACGGATTTG, R: GGGGTCGTTGATGGCAAC), mouse NF‐κB (p65, F: AGGCTTCTGGGCCTTATGTG, R: TGCTTCTCTCGCCAGGAATAC), mouse TNF‐α (F: ATGTCTCAGCCTCTTCTCATTC, R: GCTTGTCACTCGAATTTTGAGA), mouse MCP‐1 (F: TTAAAAACCTGGATCGGAACCAA, R: GCATTAGCTTCAGATTTACGGGT), mouse IL‐10 (F: GCTCTTACTGACTGGCATGAG, R: CGCAGCTCTAGGAGCATGTG), human GAPDH (F: GTCTCCTCTGACTTCAACAGCG, R: ACCACCCTGTTGCTGTAGCCAA), human TNF‐α (F: ATGGCCTCCCTCTCATCAGT, R: TTTGCTACGACGTGGGCTAC), human MCP‐1 (F: AGAATCACCAGCAGCAAGTGTCC, R: TCCTGAACCCACTTCTGCTTGG) and human IL‐10 (F: TCTCCGAGATGCCTTCAGCAGA, R: TCAGACAAGGCTTGGCAACCCA).

### Flow Cytometry

The analysis of M2 RAW 264.7 polarization was detected by flow cytometry. Cells (1x10^6^/well) were collected and centrifuged at 500 g for 5 min at 37 °C and then washed with PBS twice. The pellets were resuspended in 200 µL PBS in Eppendorf tubes and were blocked using the MouseBD Fc Block (BD Biosciences) for 1530 min at 4 °C. After blocking, 2 µL APC‐CD206 (1:100, Biolegend) and 2 µL APC750‐CD11b (1:100, Biolegend) antibody was added to each Eppendorf tube and incubated at 4 °C in the dark for 30 min. Subsequently, the cells were washed with PBS twice. Analysis of CD206 and CD11b expression levels was performed using flow cytometry (FACSort; BD Biosciences) with BD CellQuest Pro software (version 2.0, system OS2; Becton, Dickinson and Company). To ensure the accuracy of the experimental results, all samples were analysed within 3 h to avoid fluorescence changes, which could affect the experimental results. The number of detected cells per tube was 20000.^[^ ^36^ ^]^

### Colocalization Analysis by Confocal Microscopy

GFP‐ApoER2 overexpressed RAW 264.7 cells and GFP‐vector overexpressed RAW 264.7 cells were plated in laser confocal dishes (80 000 cells per well) 12 h before the experiment. The recombinant sclerostin was added to make its final concentration 1 µM for 12 h. Cells were washed three times with PBS and then fixed with 4% paraformaldehyde (PFA) in PBS for 15 min, washed three times, blocked in 10% goat serum for 1 h at room temperature and incubated with anti‐sclerostin primary antibody (Thermo, PA5‐113315, 1:100) overnight at 4 °C. Thereafter, cells were washed with PBS three times, then incubated with goat anti‐rabbit IgG H&L (Alexa Fluor 594, 1:200) for 2 h at 37 °C. Hoechst was used to stain the cell nuclei. The fluorescent images were obtained by a confocal microscope (Leica, STELLARIS STED) equipped with a 405 nm laser, a 488 nm laser, and a 594 nm laser.^[^ ^67^ ^]^

### Nuclear‐Cytoplasmic Fractionation

Nuclear and cytoplasmic extracts were isolated from cells using the Thermo Scientific NE‐PER Kit (Cat. No. 78 833). Briefly, fresh cells (1–10 × 10⁶) were resuspended in ice‐cold CER I, vortexed (15 s), and incubated on ice (10 min). After adding ice‐cold CER II, the samples were first vortexed (5 s) to ensure thorough mixing. Following incubation on ice for 1 min, the samples were vortexed (5 s) again. After centrifugation at 16 000 g at 4 °C for 5 min, the supernatant (cytoplasmic extract) was collected. The nuclear pellet was resuspended in ice‐cold NER to initiate nuclear lysis. To ensure full nuclear membrane disruption, the samples were vortexed for 15 s every 10 min over a 40 min ice incubation. After lysis, the mixtures were centrifuged at 16 000 g (4 °C) for 10 min. The supernatant (soluble nuclear extract) was collected and stored at ‐80 °C. The protein levels were determined by Western blot assay.^[^ ^38^ ^]^

### Mouse Models and Genotyping

The animal protocols were reviewed and approved by the Department of Health (DOH) of the Government of the Hong Kong Special Administrative Region and the Research Ethics Committee of Hong Kong Baptist University [Ref. No.: (24‐127) in DH/HT&A/8/2/6 Pt.10]. The mice were kept in a condition of 12‐h light/dark cycle at room temperature and were given standard chow and water ad *libitum* throughout the experiment period. The animals in poor body condition were excluded. The *ApoE^−/−^* mice and *SOST^ki^*.*ApoE^−/−^* mice were generated as previously described.^[^ ^14^ ^]^ The *ApoE^−/−^* mice, *sost^−/−^.ApoE^−/−^* mice, *ApoE^−/−^.Lrp8m*, *ApoE^−/−^*.*Lrp8m/Mac‐Lrp8* and *sost^−/−^.ApoE^−/−^.Lrp8m* mice (3‐month‐old, male) were used to genetically determine the role of macrophagic sclerostin loop2‐ApoER2 interaction in the suppressive effect of sclerostin on AA, atherosclerosis, and inflammatory responses. Additionally, *ApoE^−/−^* and *SOST^ki^.ApoE^−/−^* mice were used to pharmacologically determine the role of sclerostin loop2‐ApoER2 interaction in the suppressive effect of sclerostin on AA, atherosclerosis, and inflammatory responses (3‐month‐old, male).

Mouse genotypes were determined by PCR on tail genomic DNA. The *Lrp8m* mice was genotyped using DNA and amplified using FP 5ʹ‐ CAGGCCCAACCTTATAGTCACA ‐3ʹ and RP 5ʹ‐ CCTGCACATACCCATAACTTCG ‐3ʹ to generate 0 bp (wild‐type) or 1271 bp (targeted) amplicons, FP 5ʹ‐ TCTGAGGCGGAAAGAACCAG 3ʹ and RP 5′‐ AAGTCCTGGAAGGGACCCAAC ‐3ʹ to generate 0 bp (wild‐type) or 1211 bp (targeted) amplicons, FP 5ʹ‐ TGGTCGCTAAGGCCTTTGCTG ‐3ʹ and RP 5′‐ ACTCCTGCACATACCCCGGCTA ‐3ʹ to generate 372 bp (wild‐type) or 0 bp (targeted) amplicons. The *sost^−/−^* allele was genotyped using DNA and amplified using FP 5ʹ‐AGTGATATGGTGAGGCTGGATGC‐3ʹ and RP 5ʹ‐ GAACCTCAGTGATGGCTTAGTGG‐3ʹ to generate 6760 bp (wild‐type) or 555 bp (homozygous) amplicons, FP 5ʹ‐ACACACAATGTCTCGCCACTGT‐3ʹ and RP 5′‐CAGCTAACTGAAGAGACAGGGATAG‐3ʹ to generate 387 bp (wild‐type) or 0 bp (homozygous) amplicons. The Cx3cr1‐iCre mice were genotyped using DNA and amplified using FP 5ʹ‐ CGTGATCTGGTTTGCTGCATACAG ‐3ʹ and RP 5ʹ‐ CAGCAGGGAACCATTTCCTGTTGTT ‐3ʹ to generate 0 bp (wild‐type) or 368 bp (targeted) amplicons, FP 5ʹ‐ CGTGATCTGGTTTGCTGCATACAG ‐3ʹ and RP 5′‐ AAGACGGACAGGAAGATGGTTCCA ‐3ʹ to generate 258 bp (wild‐type) or 0 bp (targeted) amplicons.

### Mouse Model of Aortic Aneurysm and Atherosclerosis

Aortic aneurysm (AA), atherosclerosis, and inflammatory responses were induced in *ApoE^−/−^* mice with AngII infusion as previously described.^[^ ^15^ ^]^ In brief, osmotic minipumps (Model 1004, ALZET, Durect Corporation) were implanted into the subcutaneous space along the dorsal midline under anaesthesia on the right flank via an incision in the scapular region to deliver 1000 ng/kg/min of AngII (A9525, Sigma‐Aldrich) or vehicle in 3‐month‐old mice for four weeks.^[^ ^68^ ^]^

### High Performance Liquid Chromatography (HPLC)

The HPLC system was equipped with a KR100‐5‐C18‐4.6X250 column (Kromasil, K08670357) to quantify the modified ApoER2‐Pep peptide in plasma samples. The mobile phase elution gradient of phase A (0.1% Trifluoroacetic acid in 100% Acetonitrile) and phase B (0.1% Trifluoroacetic acid in 100% Water) was used. The flow rate, wavelength, and volume were 1.0 mL/min, 220 nm, and 10 µL, respectively. Standards were prepared in blank mouse plasma containing sodium heparin with different concentrations of the modified ApoER2‐Pep peptide.

### Assessment of AA and Atherosclerosis

For AA evaluation, the aortas were perfused via the left ventricle with ice‐cold EDTA buffer (1 mM) and saline immediately after sacrifice. The aortas were then isolated from the fat and connective tissues under a Zeiss Stemi 305 stereomicroscope and then fixed in 4% PFA. The aorta with or without aneurysm formation was defined according to Daugherty's modified classification.^[^ ^45^ ^]^ The incidence of AA was determined as follows: mouse number with aortic aneurysm/group mice*100%. The maximum *ex vivo* diameters of the aortic arch and suprarenal aorta were determined by ImageJ software. For atherosclerosis assessment, atherosclerotic plaque was quantified by measuring the surface area of the Oil Red O‐positive lesions on *en face* preparation of aortic arches.^[^ ^14^ ^]^ Briefly, aortic arches were cut longitudinally and immersed in Oil Red O dye solution, followed by being rinsed with 75% ethanol. The ratio of atherosclerotic plaque area to total aortic arch area was quantified by colorimetric analysis using Image J software.^[^ ^13^ ^]^

### Serum Sclerostin, Pro‐inflammatory Cytokine and Chemokine, Anti‐Inflammatory Cytokine Evaluation

The serum levels of sclerostin were detected using the sclerostin ELISA kits (R&D, DSST00; Cloud‐Clone, SEC864Mu) in triplicate following the manufacturer's instructions.^[^ ^14^ ^]^ The serum levels of pro‐inflammatory cytokine and chemokine were measured using TNF‐α ELISA kit (Thermo Fisher BMS607‐3) and MCP‐1 ELISA kit (Thermo Fisher, BMS6005) in triplicate following manufacturer's instructions.^[^ ^15^, ^45^ ^]^ The serum levels of anti‐inflammatory cytokine were measured using IL‐10 ELISA kit (Jonlnbio JL20242‐96T) in triplicate following manufacturer's instructions.^[^ ^15^, ^45^ ^]^

### Immunofluorescence Analysis (IF)

For *the* in vitro study, NF‐κB p65 immunofluorescence was performed to measure the nuclear translocation of p65.^[^ ^37^ ^]^ Briefly, RAW 264.7 cells were seeded in laser confocal dishes and were stimulated with 1 µg/mL LPS for 15 min. Cells were fixed with 4% PFA in PBS for 15 min at room temperature. Coverslips were incubated sequentially with 0.1% Triton‐X100 (Thermo Scientific, 9002‐93‐1) (10 min, room temperature), blocking buffer, NF‐κB p65 antibody (CST, 8242, 1:400, overnight, 4 °C), and goat anti‐rabbit IgG H&L (Alexa Fluor 594, 1:200, 2 h at 37 °C). Nuclei were counterstained with Hoechst. For the in vivo study, the paraffin cross‐sections from the aortic root (10 µm) were obtained for IF. The double‐labeled immunofluorescence staining was performed using TSA (Tyramide signal amplification) technology.^[^ ^69^ ^]^ Briefly, deparaffinized sections were rehydrated, boiled to retrieve antigens (10 mM citrate buffer, pH 6), and blocked with 3% BSA. The first primary antibody was added and incubated overnight at 4 °C. After adding the corresponding HRP‐labeled secondary antibody, TSA was added dropwise and incubated for 10 min at room temperature away from light. Further, microwave processing was performed to remove the primary and secondary antibodies. The above steps were repeated for the next antibody. Finally, we counterstained nuclei using DAPI and mounted and applied coverslips on the slides. The antibodies and TSA dyes used were listed below: anti‐iNOS antibody (Servicebio, GB11119, 1:2000), anti‐CD206 antibody (Servicebio, GB113497, 1:2000), goat anti‐rabbit IgG (Servicebio, GB23303, 1:500), Cy3‐Tyramide (Servicebio, G1223, 1:500), and iF488‐Tyramide (Servicebio, G1231, 1:500). The ratio of M2 to M1 (M2/M1) was determined as follows: number of CD206^+^ cells/iNOS^+^ cells. The number of CD206^+^ or iNOS^+^ cells was quantified using QuPath, an open‐source software for digital pathology image analysis. Regions of interest (ROIs) were the whole aortic root, which was annotated by white dotted line in the figures. Positive cells were identified based on specific staining intensity thresholds, followed by automated cell counting to ensure accuracy and reproducibility. For each mouse, 6 randomly selected tissue sections were analyzed in whole microscope fields.^[^ ^47^ ^]^

### Immunohistochemistry (IHC)

Formalin‐fixed and paraffin‐embedded aortic roots originated from mice of each group were deparaffinized in xylene and rehydrated in alcohol. Antigen retrieval was carried out using a heat‐mediated method in Tris/EDTA buffer (pH 9.0). The adjacent slices were incubated in the 3% H_2_O, solution to block endogenous peroxidase. After incubation in 3% BSA for 30 min, the sections were incubated overnight at 4 °C with phospho‐NF‐κB p65 antibody (CST, 3033) diluted at 1:1000. Following washing in PBS they were labeled with HRP‐linked anti‐rabbit IgG (Bio‐Rad, STAR124P, 1:5000) for 50 min at room temperature in a humidified chamber. After washing in PBS (pH 7.4), the label was visualized with DAB (DA1010, Solarbio, Beijing).^[^ ^15^, ^48^ ^]^ QuPath (v0.5.1) was used for p‐p65 positive cell quantification. Total and p‐p65 positive cells were calculated using the Cell Detection function (Analyze → Cell Detection → Positive Cell Detection) in the specified ROIs, with mean values used for statistical analysis. Positive cell percentage was calculated as (p‐p65 positive cells / total cells) × 100(%).^[^ ^70^ ^]^

### Masson's Trichrome Staining

Formalin‐fixed aortic roots were dehydrated in graded ethanol (50% to 100%), cleared in xylene, and embedded in paraffin. Cryosections (7 µm) at the level of the aortic roots were deparaffinized in xylene and rehydrated via graded ethanol, with a final rinse in distilled water. Nuclear staining was performed by incubating sections in Weigert's iron hematoxylin, differentiated in 1% hydrochloric acid‐ethanol, and blued in Masson's blueing solution. Subsequent cytoplasmic and collagen staining involved incubating sections in Biebrich scarlet‐acid fuchsin, treating with phosphomolybdic‐phosphotungstic acid, followed by staining with aniline blue. After brief differentiation in 1% acetic acid, sections were dehydrated, cleared, and mounted. Collagen (blue), smooth muscle (red), and nuclei (black‐blue) were visualized for histological analysis. The percentage of collagen fiber area (% area) was calculated as the ratio of the stained area (blue) to the total area of atherosclerotic plaque. When no arterial plaques are present, the collagen fiber (% area) was defined as 0.^[^ ^46^ ^]^

### Statistical Analysis

All variables were expressed as mean ± standard deviation. Unpaired t‐test, One‐way ANOVA with Tukey's post‐hoc test, and Two‐way ANOVA with Tukey's post‐hoc test were performed to determine the intergroup differences in the in vitro study variables (n = 3 per group), including mRNA expression levels of inflammatory cytokines and chemokines, medium protein levels of inflammatory cytokines and chemokines, proportion of anti‐inflammatory macrophage phenotypes, parameters regarding NF‐κB nuclear translocation, as well as the intergroup differences in the in vivo study variables (n = 12 per group), including serum levels of inflammatory cytokines and chemokines, serum levels of sclerostin, and parameters regarding AA and atherosclerosis progression. For the AA incidence in mice, Fisher's exact test was performed to determine the intergroup differences. All statistical tests were two‐sided and performed using GraphPad Prism (version 8; GraphPad Software, Inc., San Diego, CA, USA), and *P < 0.05* was considered to be statistically significant. For the in vivo experiments, the sample size was pre‐determined by a power calculation according to our previously published protocol.^[^ ^71^ ^]^ The animals were grouped randomly and blindly. The animals in poor body condition were excluded.

## Conflict of Interest

The authors declare no conflict of interest.

## Author Contributions

L.W., X.T., N.Z., X.Y., and H.J. contributed equally to this work. G.Z., L.W., B.Z., and A.L. supervised the project. L.W., X.T., N.Z., X.Y., and H.J. performed the major research, analyzed and interpreted data, and wrote the manuscript in equal contributions. S.D., X.Y. and Y.Z. helped with complex structure modeling and molecular dynamics simulation. X.L., S.W., S.Y., N.L., H.L., Z.L., H.Z., X.W., M.S., C.Z., and X.S. contributed to crossbreeding and genotyping of mice, micro‐CT analysis, as well as animal experiments. D.M., Y.H., P.F., S.Z., T.Z., X.L., J.L., and Y.Y. provided their professional expertise. All authors revised the content and approved the submitted version.

## Supporting information



Supporting Information

## Data Availability

The data that support the findings of this study are available from the corresponding author upon reasonable request.
