# Supplementary material for: Macrophagic Sclerostin Loop2‐ApoER2 Interaction Required by Sclerostin for Cardiovascular Protective Action
Source: Adv Sci (Weinh). 2025 Nov 23;13(8):e18735. doi: 10.1002/advs.202518735 (PMC12884772; doi:10.1002/advs.202518735)

**Supplementary Materials for**

**Macrophagic Sclerostin Loop2-ApoER2 Interaction Required by Sclerostin for Cardiovascular Protective Action**

Luyao Wang^1,2,3,4#^*, Xiaohui Tao^1,2,3,4#^, Ning Zhang^5#^, Xin Yang^1,2,3,4#^, Hewen Jiang^5#^, Xiaofei Li^1,2,3,4^, Shenghang Wang^1,2,3,4^, Shijian Ding^1,2,3,4^, Sifan Yu^1,2,3,4^, Huarui Zhang^5^, Yihao Zhang^5^, Nanxi Li^1,2,3,4^, Haitian Li^1,2,3,4^, Zhanghao Li^1,2,3,4^, Xiaoxin Wen^1,2,3,4^, Meiheng Sun^1,2,3,4^, Chuanxin Zhong^1,2,3,4^, Jin Liu^1,2,3,4^, Yuanyuan Yu^1,2,3,4^, Xianghang Luo^6^, Tao Zhang^7^, Shu Zhang^8^, Péter Ferdinandy^9,10,11^, Yu Huang^12^, Daqing Ma^13,14^, Aiping Lu^1,2,3,4^*, Baoting Zhang^5^*, Ge Zhang^1,2,3,4^*

^#^ Luyao Wang, Xiaohui Tao, Ning Zhang, Xin Yang and Hewen Jiang contributed equally to this work.

*Correspondence and requests for materials could be addressed to Ge Zhang ([zhangge@hkbu.edu.hk](mailto:zhangge@hkbu.edu.hk)), Luyao Wang ([luyaowang@hkbu.edu.hk)](mailto:luyaowang@hkbu.edu.hk)), Baoting Zhang ([zhangbaoting@cuhk.edu.hk](mailto:zhangbaoting@cuhk.edu.hk)) and Aiping Lu ([aipinglu@hkbu.edu.hk](mailto:aipinglu@hkbu.edu.hk)).

The file includes:

**Figure S1**. Quality control and feature selection metrics for single-cell RNA sequencing data.

**Figure S2**. Ridge plot of gene expression across macrophage clusters identified by Leiden clustering (clusters 0, 1, 2, 3 and 4).

**Figure S3**. Analysis of lineage differentiation trajectories for macrophage populations in aorta tissues from *ApoE^-/-^* mice and *SOST^ki^.ApoE^-/-^* mice.

**Figure S4**. The suppressive effects of sclerostin on inflammatory responses were dependent on ApoER2 in macrophages *in vitro*.

**Figure S5**. GWAS analysis indicated the association between sclerostin loop2-specific mutations and cardiovascular abnormalities.

**Figure S6**. Binding analysis for the interaction between sclerostin and ApoER2 *in vitro*.

**Figure S7**. Design and characterization of *Lrp8* mutation tool (*Lrp8m*) and ApoER2-Pep peptide tool for genetic and pharmacologic blockade of sclerostin loop2-ApoER2 interaction.

**Figure S8**. Construction and sequencing of *Lrp8m* mouse model.

**Figure S9**. Genotyping of *ApoE^-/-^* mouse model, *sost^-/-^.ApoE^-/-^* mouse model, *ApoE^-/-^.Lrp8m* mouse model, *ApoE^-/-^.Lrp8m/Mac-Lrp8* mouse model, *sost^-/-^. ApoE^-/-^.Lrp8m* mouse model and *SOST^ki^.ApoE^-/-^* mouse model.

**Figure S10**. Characterization and determination of arterial fibrosis in *ApoE^-/-^* mice, *sost^-/-^.ApoE^-/-^* mice, *ApoE^-/-^.Lrp8m* and *ApoE^-/-^.Lrp8m/Mac-Lrp8* mice.

**Figure S11**. Characterization of *sost^-/-^*.*ApoE^-/-^* mice and *sost^-/-^*.*ApoE^-/-^.Lrp8m* mice, with and without re-expression of sclerostin.

**Figure S12**. Determination of administration dosage, interval and duration of the modified ApoER2-Pep for *in vivo* studies.

**Table S1**. Full-length and truncated ApoER2.

**Table S2**. Amino acid sequences of LA6LA7 domains within WT and mutated ApoER2.

**Table S3.** Amino acid sequences of loop2 domain within WT and mutated sclerostin.


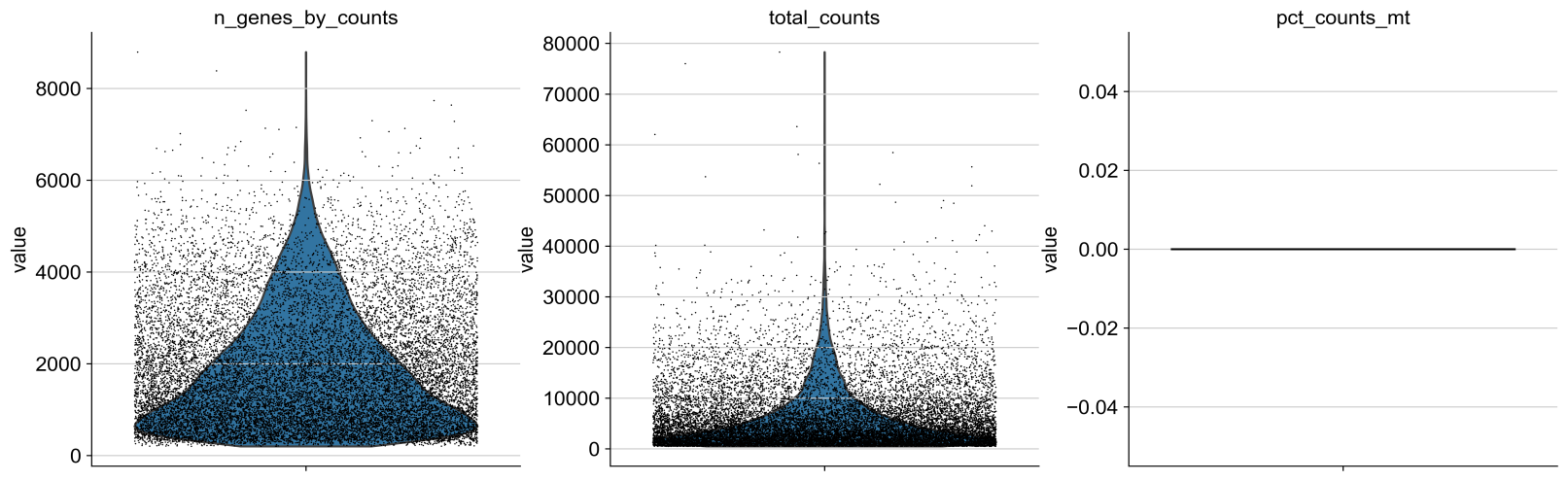


**A**

**C**

**B**


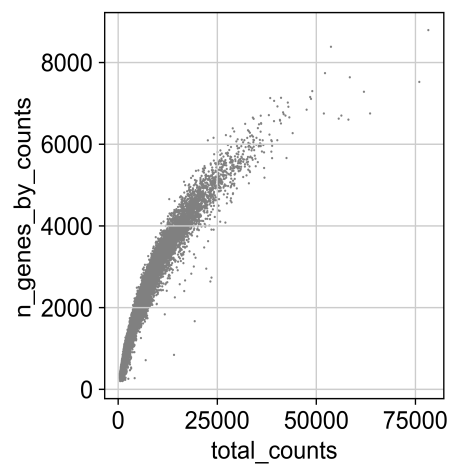


**D**


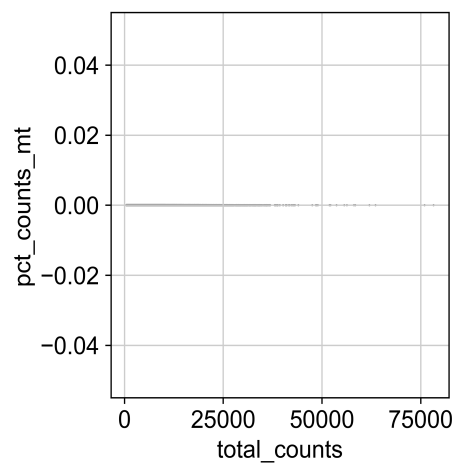


**E**


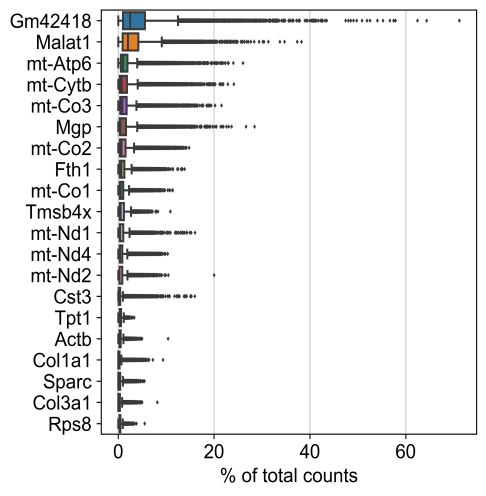


**F**


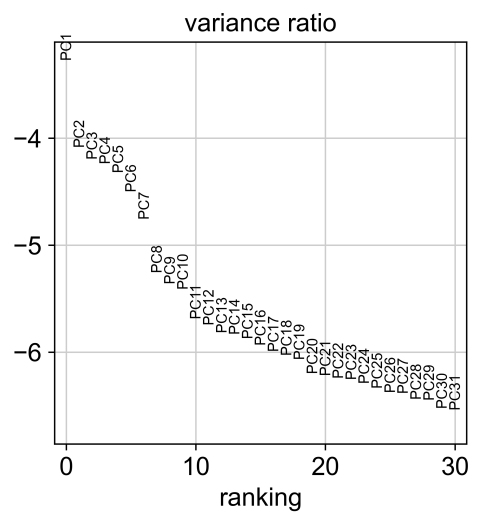


**I**


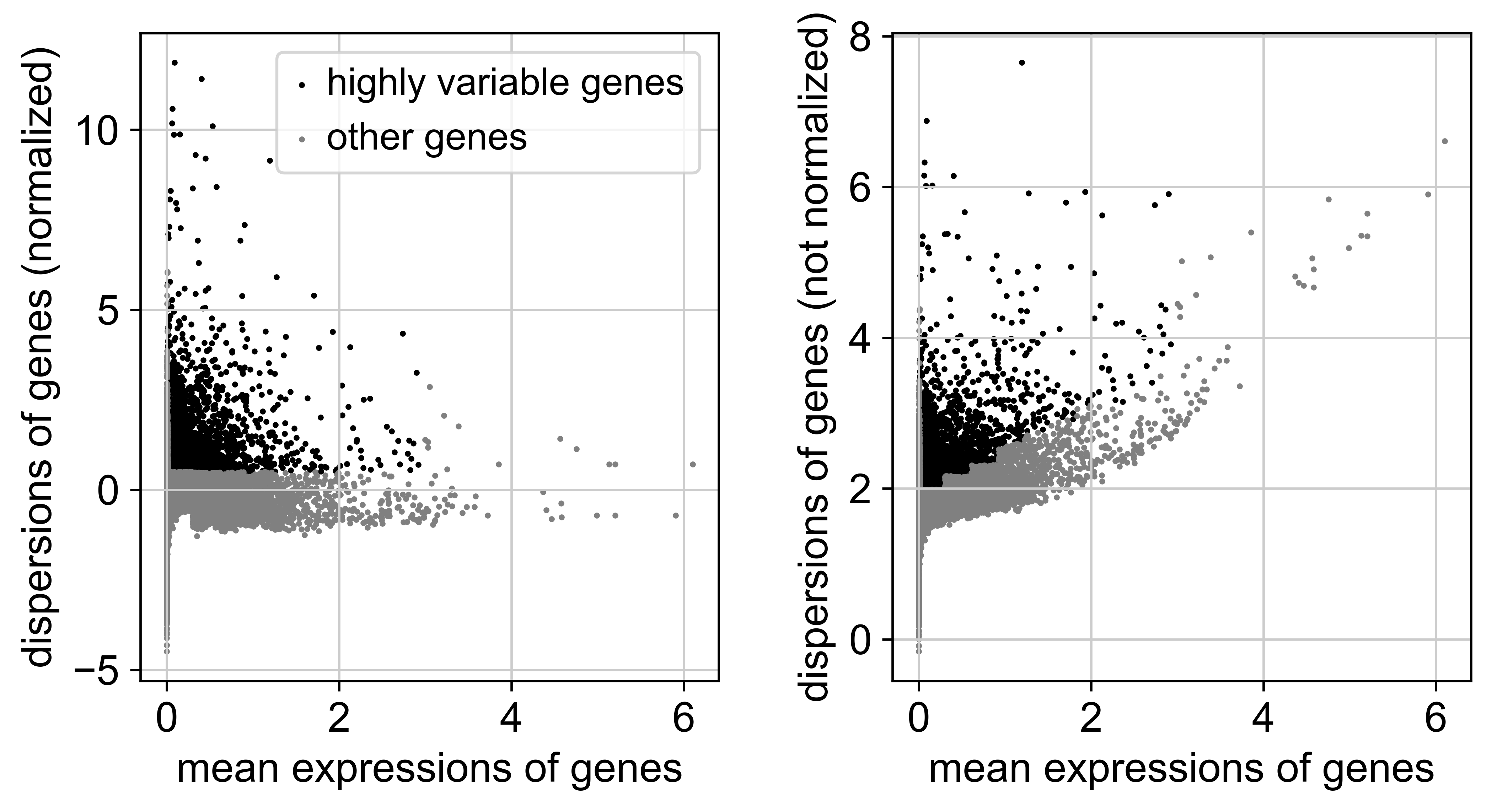


**H**

**G**


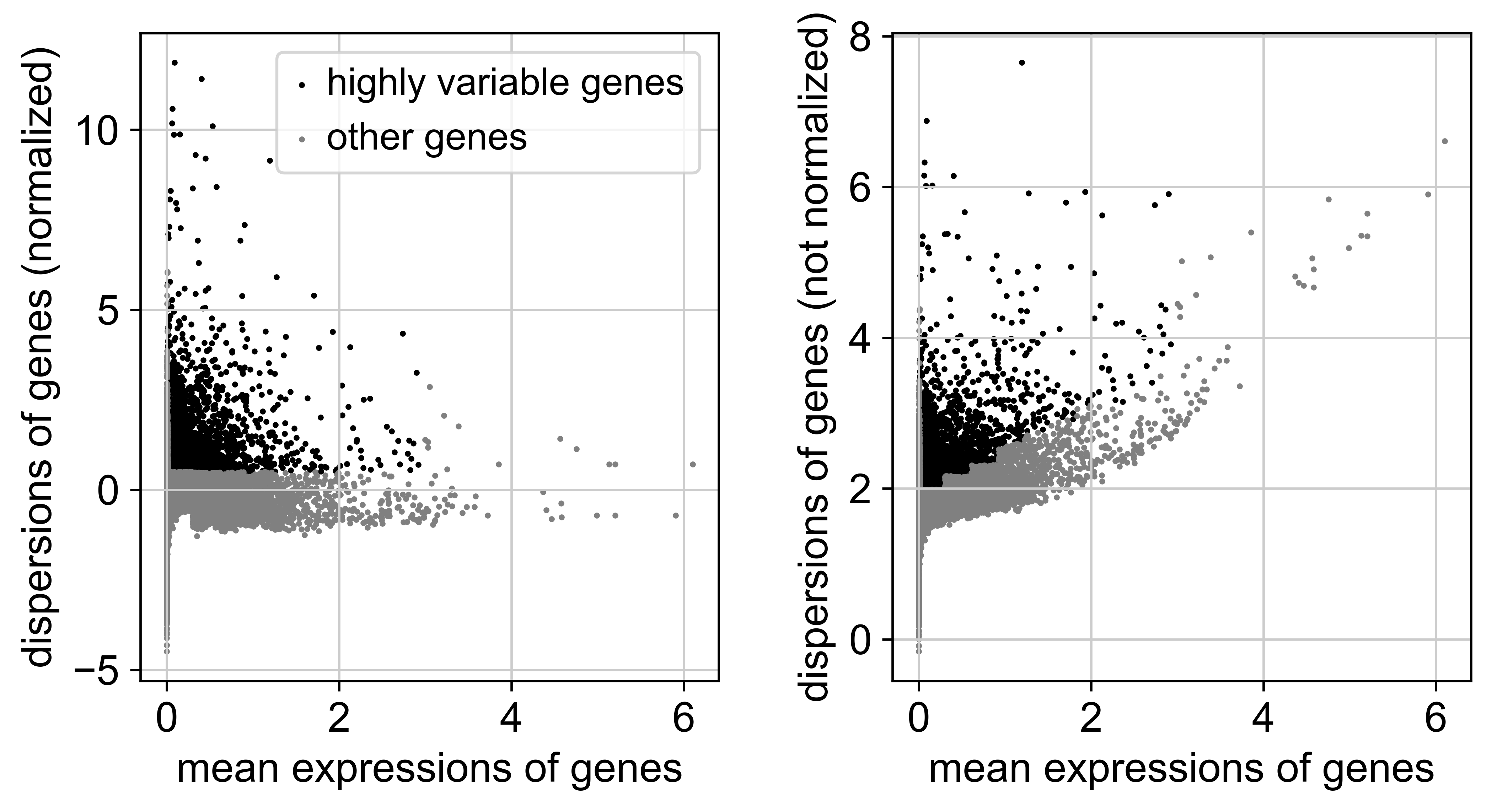


**Figure S1. Quality control and feature selection metrics for single-cell RNA sequencing data.** **(A)** Violin plot showing the distribution of the number of genes detected per cell (**n_genes_by_counts**). The majority of cells had been detected with 1,000 to 6,000 genes, which represented typical characteristics of high-quality single-cell data. **(B)** Violin plot showing the total counts per cell (**total_counts**). Most cells exhibited total counts between 10,000 and 40,000, indicating expected transcriptional activity. Cells with unusually high or low total counts were exclued during quality control. **(C)** Violin plot showing the percentage of mitochondrial counts per cell (**pct_counts_mt**). Low mitochondrial percentages across most cells suggested good RNA integrity and minimal cell stress, ensuring high-quality single-cell RNA data. **(D)** Scatter plot of the number of genes detected per cell (**n_genes_by_counts**) versus the total counts per cell (**total_counts**). The positive correlation indicated that cells with higher RNA content tend to detect more detected genes, reflecting consistent sequencing quality across cells. **(E)** Scatter plot of the percentage of mitochondrial counts (**pct_counts_mt**) versus the total counts per cell (**total_counts**). The mitochondrial percentage remained close to zero across all cells, indicating minimal mitochondrial RNA contamination and high-quality cell integrity. **(F)** Bar plot showing the top genes expressed across all cells as a percentage of total counts. The top genes included mitochondrial genes (e.g., **mt-Atp6**, **mt-Co1**) and other highly expressed genes (e.g. **Malat1**, **Gm42418**), providing insights into common transcriptional profiles across the dataset. **(G)** Scatter plot showing gene variability after normalization. Highly variable genes (black points) were selected based on their elevated dispersion relative to mean expression. These genes were used in downstream analyses to capture cell heterogeneity. **(H)** Scatter plot showing the dispersion of genes without normalization. This plot illustrated the natural variability of gene expression in the dataset, helping the identification of genes that contributed most to cellular heterogeneity. **(I)** Variance ratio plot showing the ranking of the first 30 principal components (PCs) based on the variance calculated through the covariance matrix. The first 10 PCs captured most of the meaningful variation, as indicated by their higher variance ratios.


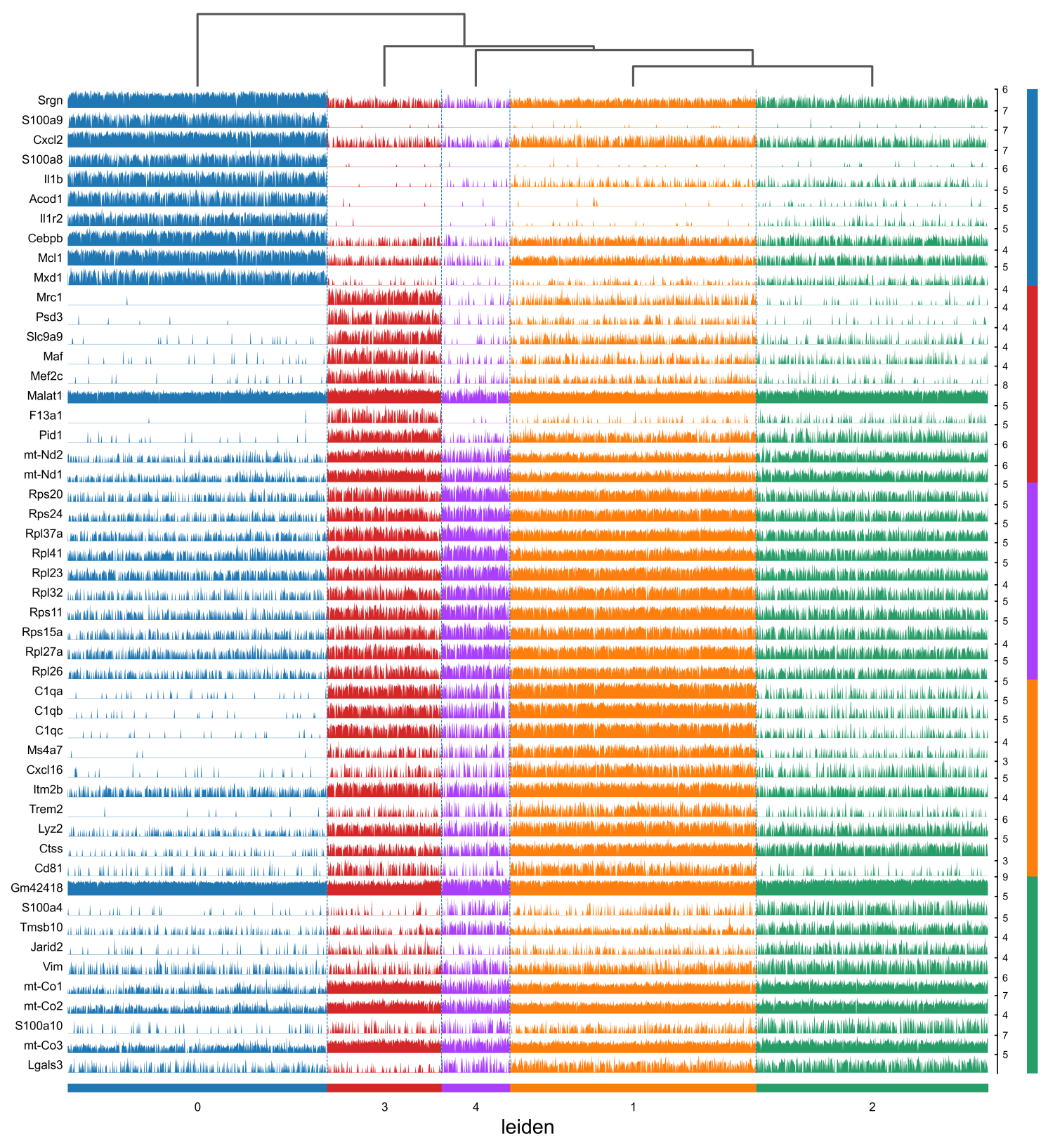


**Figure S2.** **Ridge plot of gene expression across macrophage clusters identified by Leiden clustering (clusters 0, 1, 2, 3 and 4).** Each row represented a specific gene, while each column corresponded to an individual cell, organized by cluster. Colors indicated expression levels, ranging from low (blue) to high (red). The hierarchical dendrogram at the top showed the similarity between clusters, revealing transcriptional diversity among macrophage subtypes. Specific genes such as *S100a8*, *Mrc1*, and *Trem2* highlighted functional distinctions between clusters. **Note:** Clusters 0, 1, 2, 3, and 4 represented the following macrophage sub-types: Cluster 0: M1-like 1, Cluster 1: M1-like 2, Cluster 2: M2-like, Cluster 3: Res-like 1, and Cluster 4: Res-like 2.


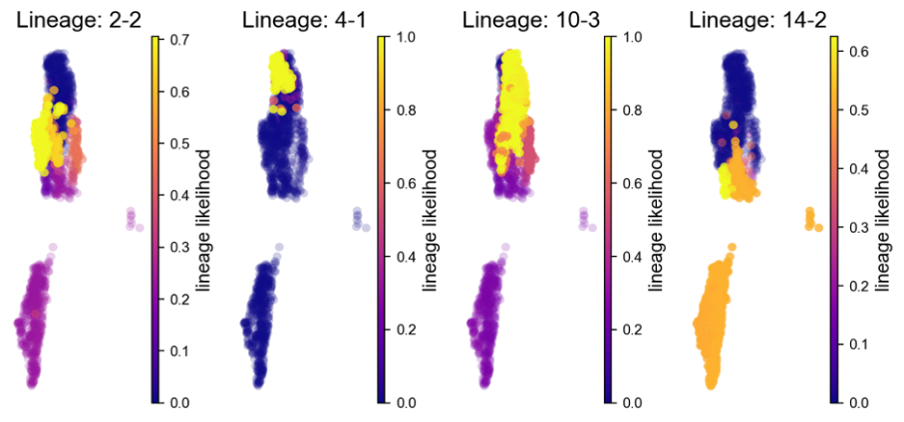


**A**


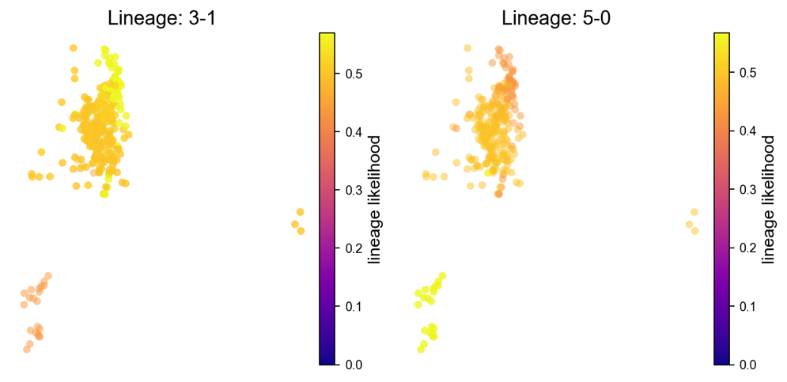


**B**

**Figure S3. Analysis of lineage differentiation trajectories for macrophage populations in aorta tissues from *ApoE^-/-^* mice and *SOST^ki^.ApoE^-/-^* mice.** **(A)** Pseudotime lineage differentiation graph specifically for macrophages in *ApoE^-/-^* mice. Lineages and differentiation paths were indicated by different colors. **(B)** Pseudotime lineage differentiation graph specifically for macrophages in *SOST^ki^.ApoE^-/-^* mice. The lineages were color-coded to show differentiation paths influenced by sclerostin knock-in.

**A**

**B**


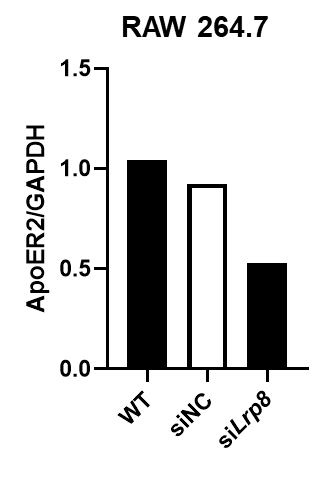

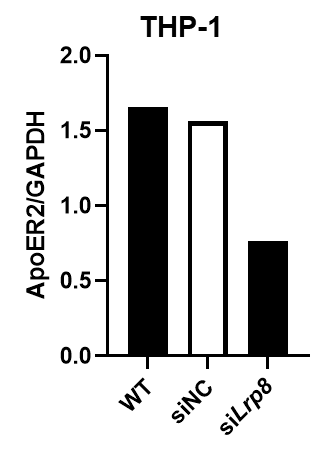

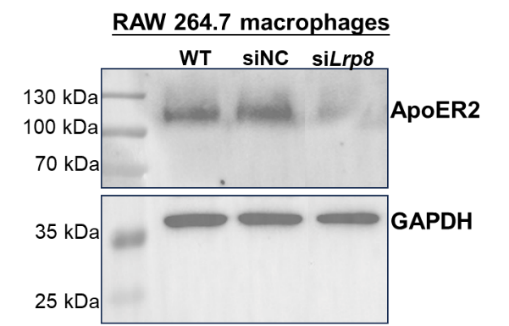

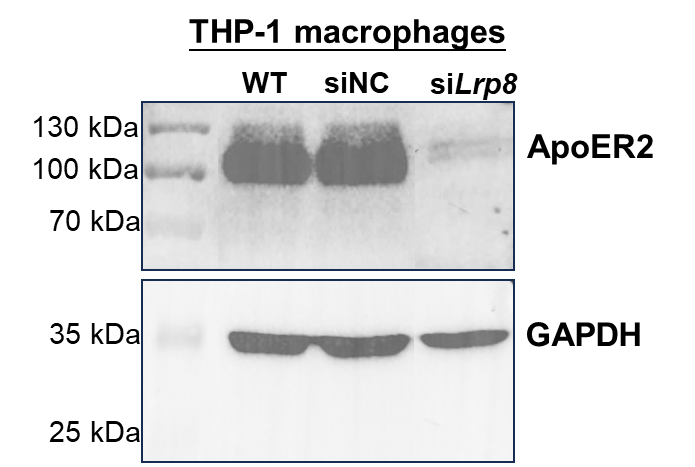


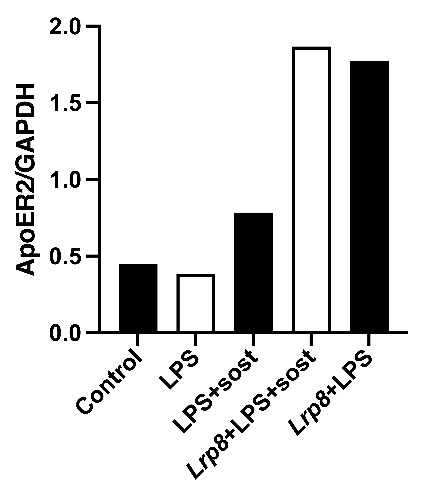


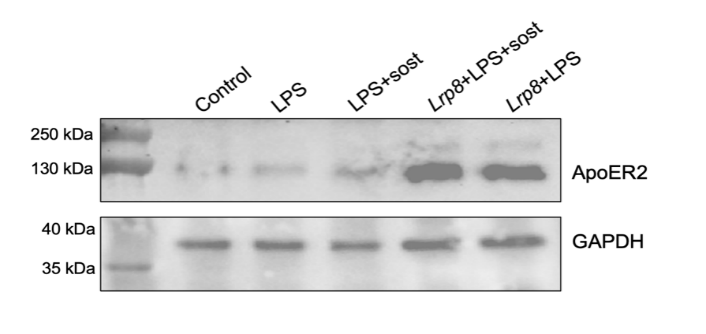


**C**

**D**


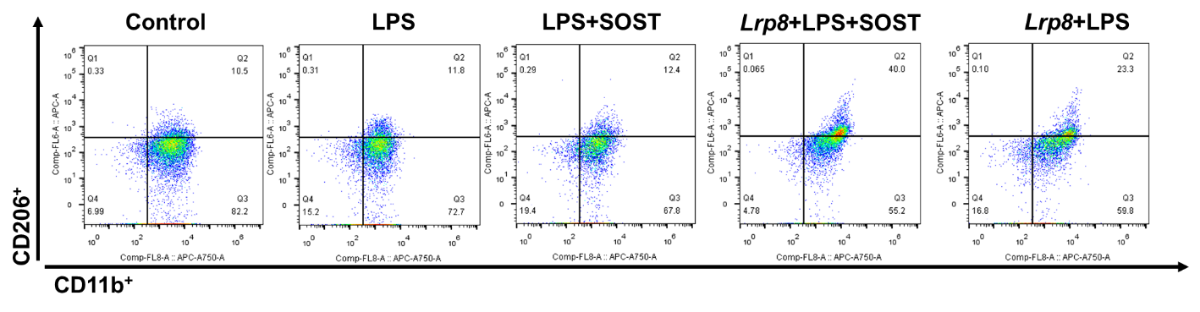


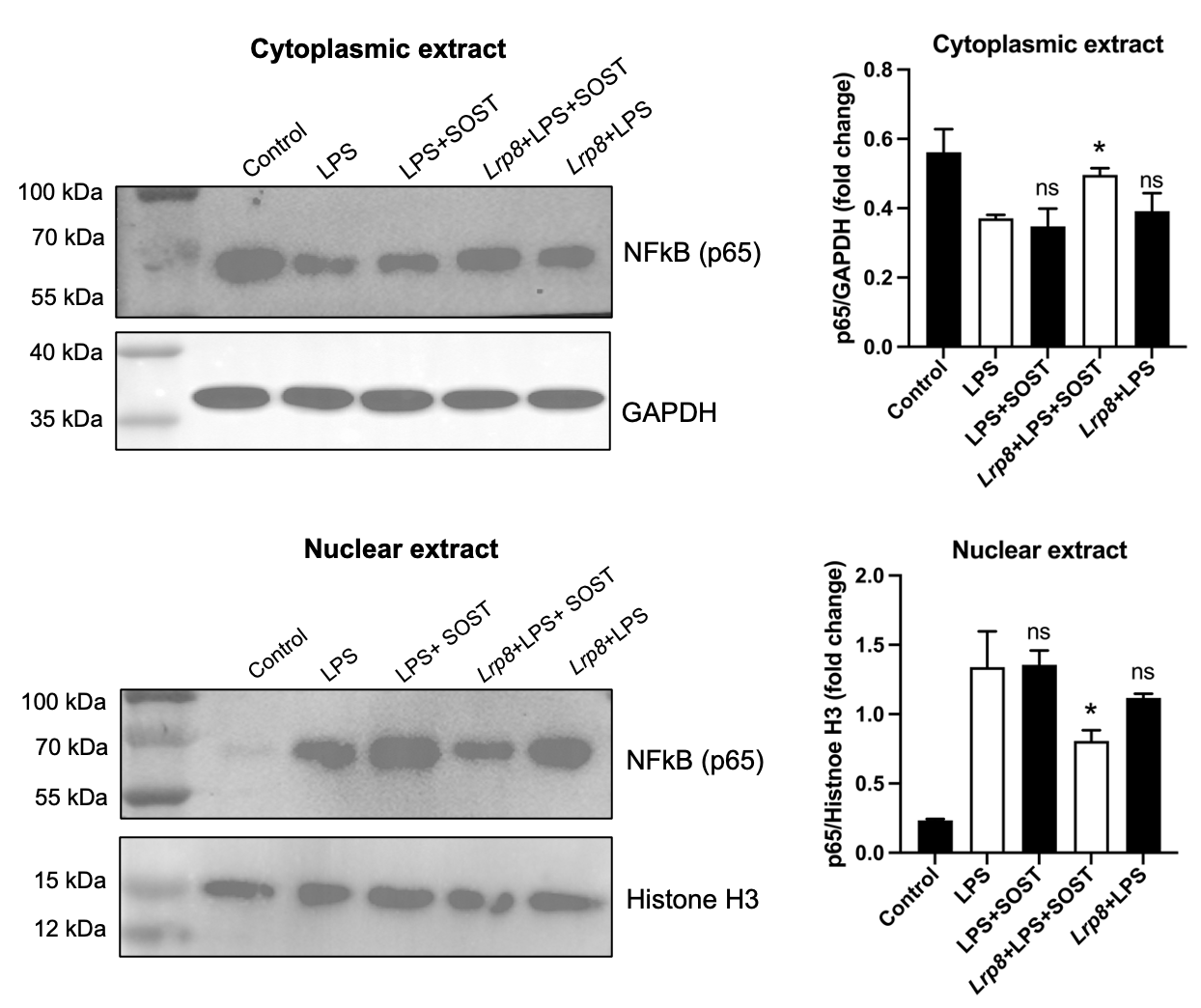


**E**

**Figure S4. The suppressive effects of sclerostin on inflammatory responses were dependent on ApoER2 in human macrophages and mouse macrophages *in vitro*. (A)**The interference efficiency of siRNA targeting human *Lrp8* (si*Lrp8*) was examined in human differentiated macrophages (THP-1) by western blot (left). Quantification of the density of detected bands (ApoER2/GAPDH) in western blot assay (right). **(B)** The interference efficiency of siRNA targeting mouse *Lrp8* (si*Lrp8*) was examined in mouse macrophages (RAW 264.7) by western blot (left). Quantification of the density of detected bands (ApoER2/GAPDH) in western blot assay (right). **(C)** Western blot analysis for the protein level of ApoER2 in mouse macrophages (RAW 264.7) (left). Quantification of the density of detected bands in western blot (right). **(D)** Surface markers examined by flow cytometry depict expression of CD206 and CD11b in mouse macrophages (RAW 264.7), with LPS (1 μg/mL) induction for 12 hours (left). Quantification of the proportion of anti-inflammatory phenotype (CD206^+^) (right) (n=2 per group). **(E)** Immunoblotting of p65 in the cytoplasmic extract of mouse macrophages (RAW 264.7), with LPS (1 μg/mL) induction for 30 minutes (upper left). Quantification of the density of detected bands in the cytoplasmic extract of RAW264.7 macrophages (upper right) (n=2 per group). Immunoblotting of p65 in the nuclear extract in mouse macrophages (RAW 264.7) with LPS (1 μg/mL) induction for 30 minutes (lower left). Quantification of the density of detected bands in the nuclear extract of RAW264.7 macrophages (lower right) (n=2 per group). Data was expressed as mean ± standard deviation. One-way ANOVA with Tukey’s post-hoc test *vs.* LPS group was used to determine the intergroup differences. All tests were two-sided.  ^ns^ *P > 0.05, * P < 0.05, ** P < 0.01, *** P < 0.001, **** P < 0.0001*. **Note**: LPS: lipopolysaccharide; TNF-α: tumor necrosis factor alpha; MCP-1: monocyte chemoatractant protein-1; IL-10: interleukin-10. CD11b: alpha chain of the macrophage-1 receptor (macrophage marker); CD206: cluster of differentiation 206, known as mannose receptor C-type 1 (anti-inflammatory macrophages biomarker).

**A**


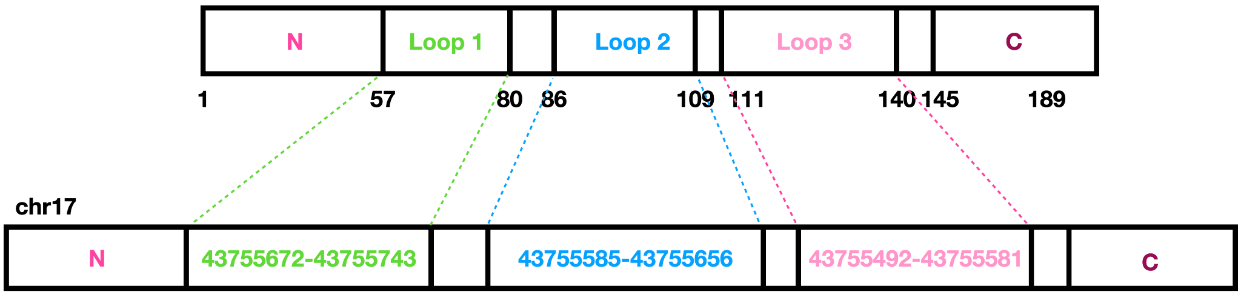


**B**


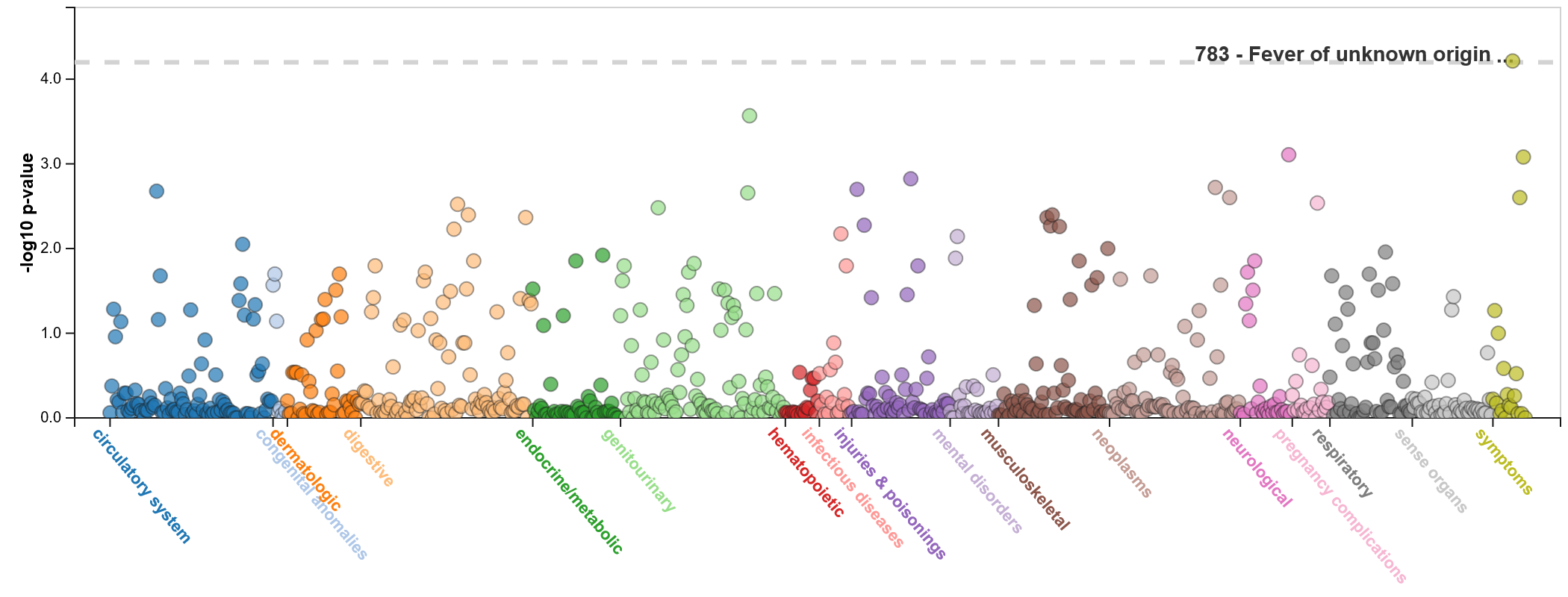


**C**


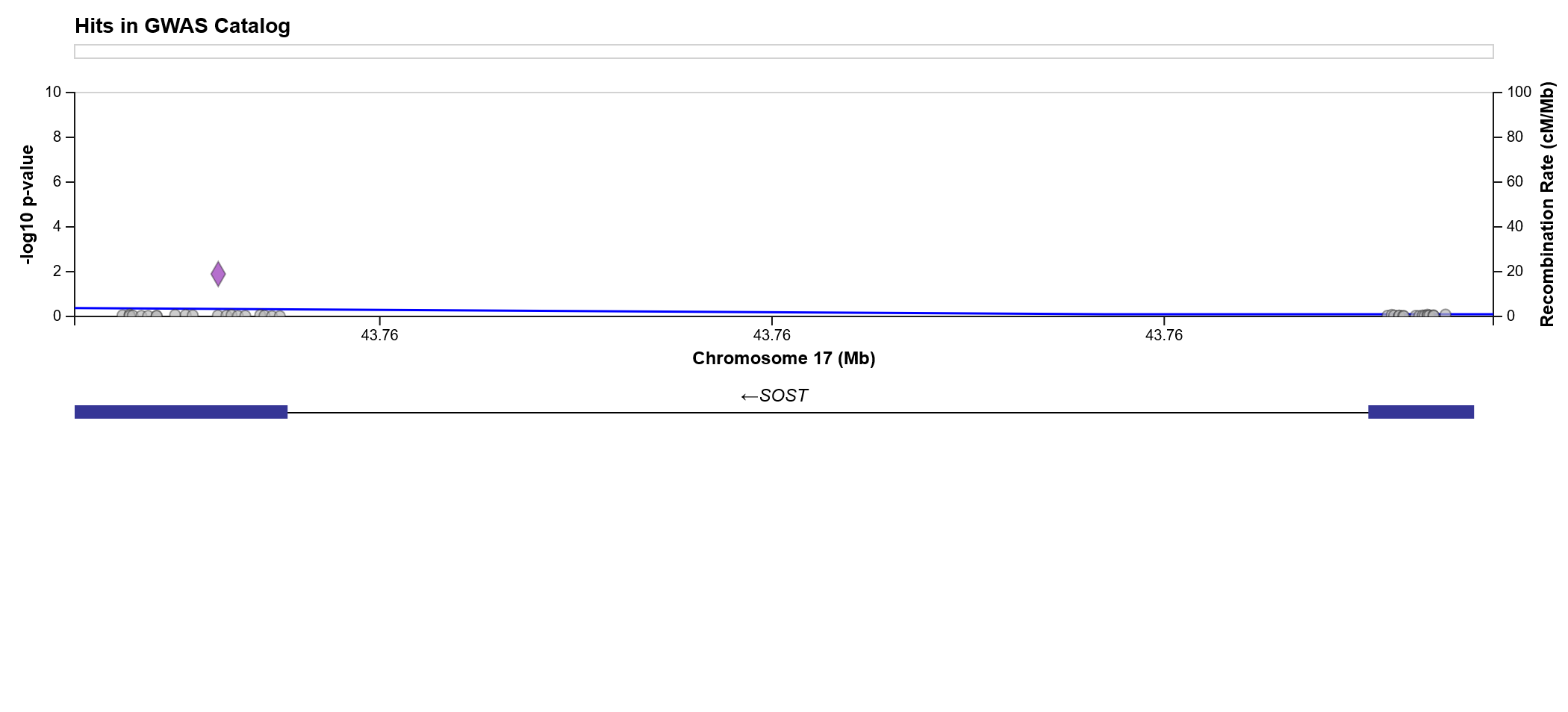


chr17 : 43,755,588 G/C (SOST + 427.3, Cardiac dysrhythmias)


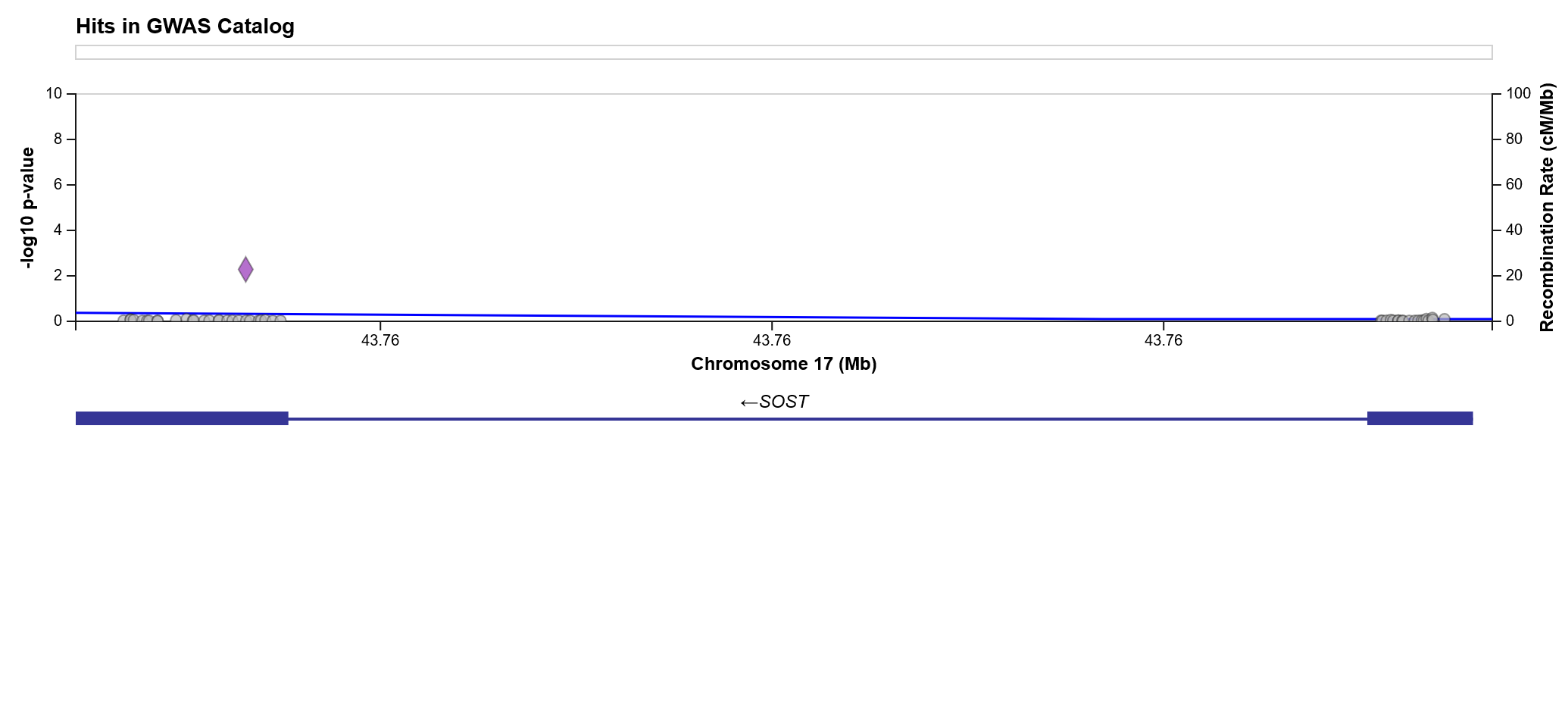


chr17 : 43,755,656 C/T (SOST + 418.1, Precordial pain)

**D**


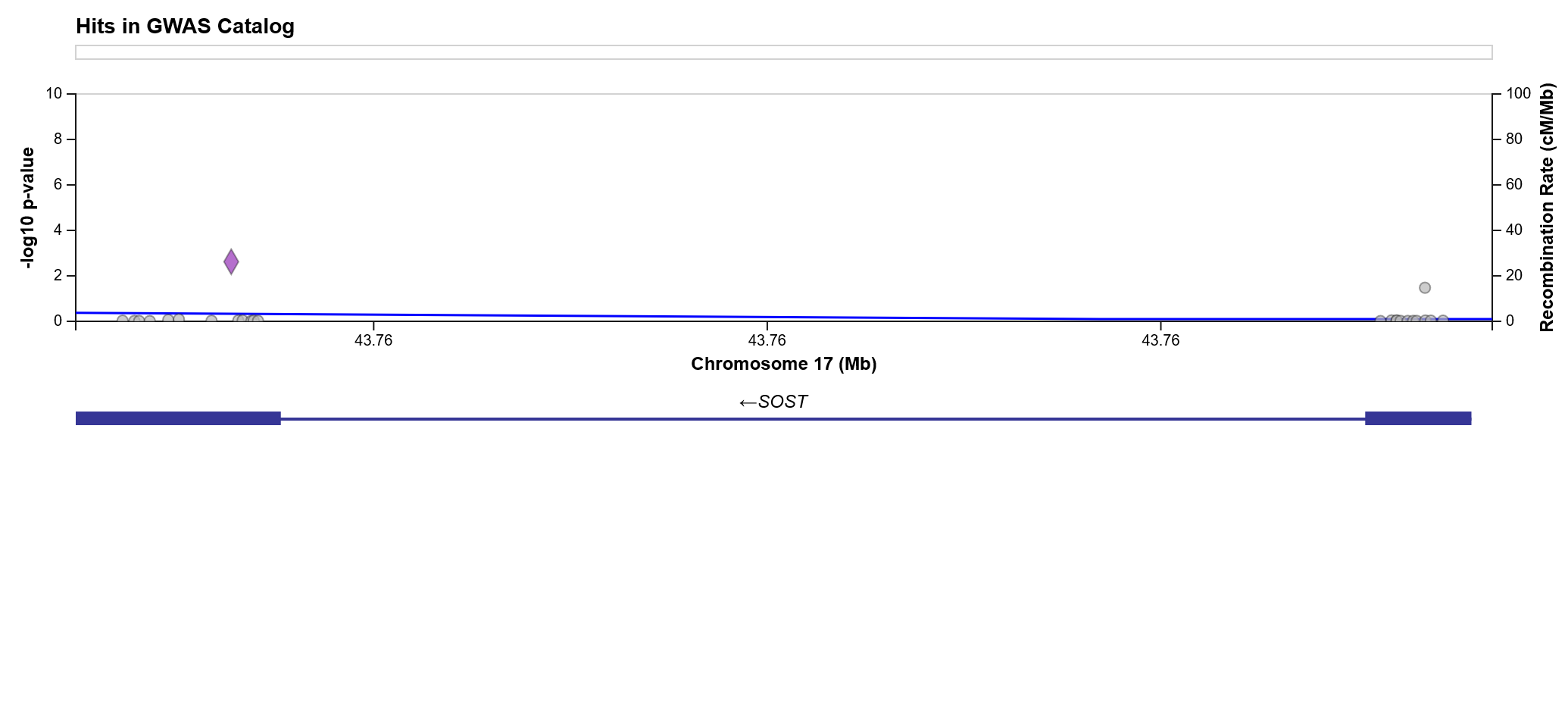


chr17 : 43,755,638 G/A (SOST + 443.9, Peripheral vascular disease, unspecified)

**E**

**Figure S5. GWAS analysis indicated the association between sclerostin loop2-specific mutations and cardiovascular abnormalities. (A)** Schematic representations of the sclerostin protein and *SOST* gene. **(B)** Manhattan plot and phenogram of the GWAS analysis for the *SOST* variants in the UK Biobank (UKB). **(C)** The minor allele of the single-nucleotide polymorphisms (SNPs) rs879666342 (chr17: 43,755,588, G > C; G allele frequency in UKB, 2.98e-5) was associated with higher Cardiac dysrhythmias [*P = 0.013*, per C allele]. **(D)** The minor allele of rs886052981 (chr17: 43,755,656, C > T; C allele frequency in UKB 1.20e-5) was associated with higher Precordial pain [*P = 0.005*, per C allele]. **(E)** The minor allele of rs765435662 (chr17: 43,755,638, G > A; G allele frequency in UKB, 2.63e-5) was associated with higher Peripheral vascular disease [*P = 0.002*, per G allele]. **Note:** The diamonds represented the significant association signals identified in the GWAS analysis, showing specific loci associated with cardiovascular abnormalities.

**C**

**A**


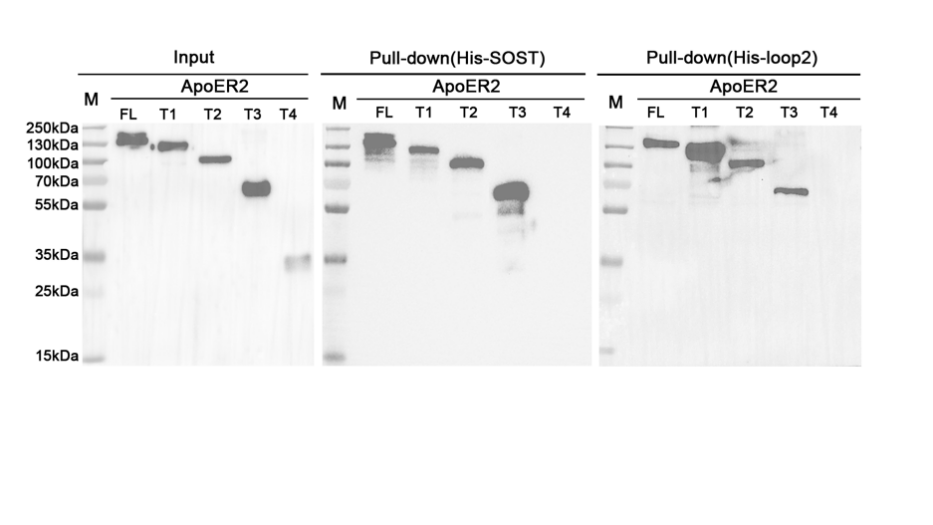

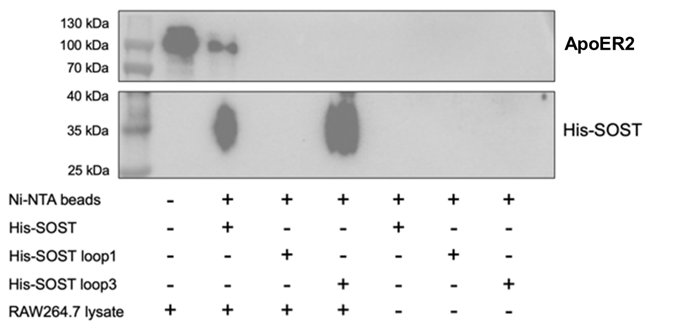


**B**

**D**

**Sclerostin – ApoER2, *K_d_*=2.1 nM**


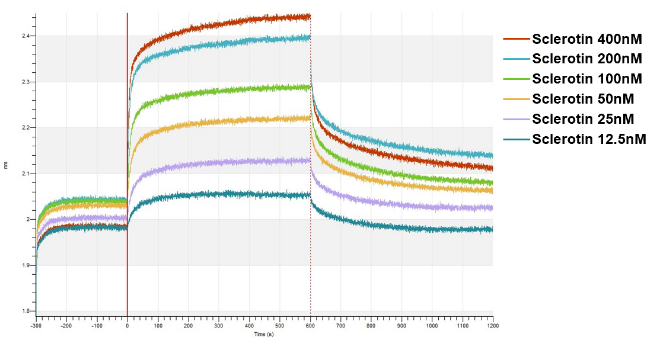

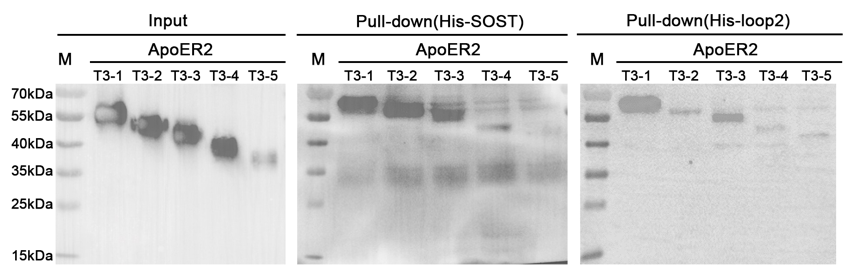


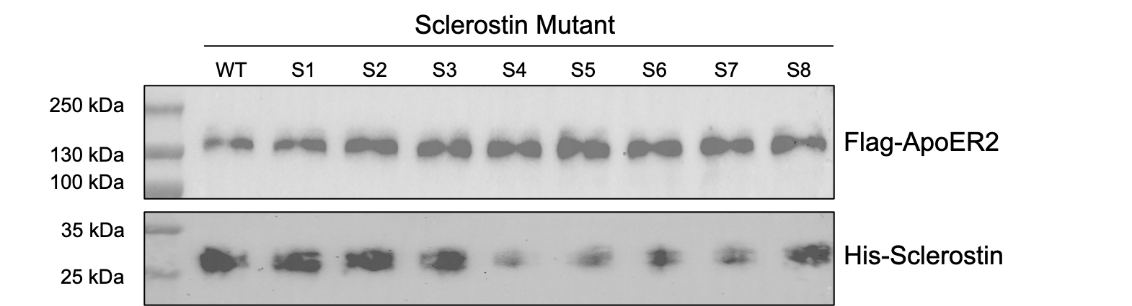


**E**


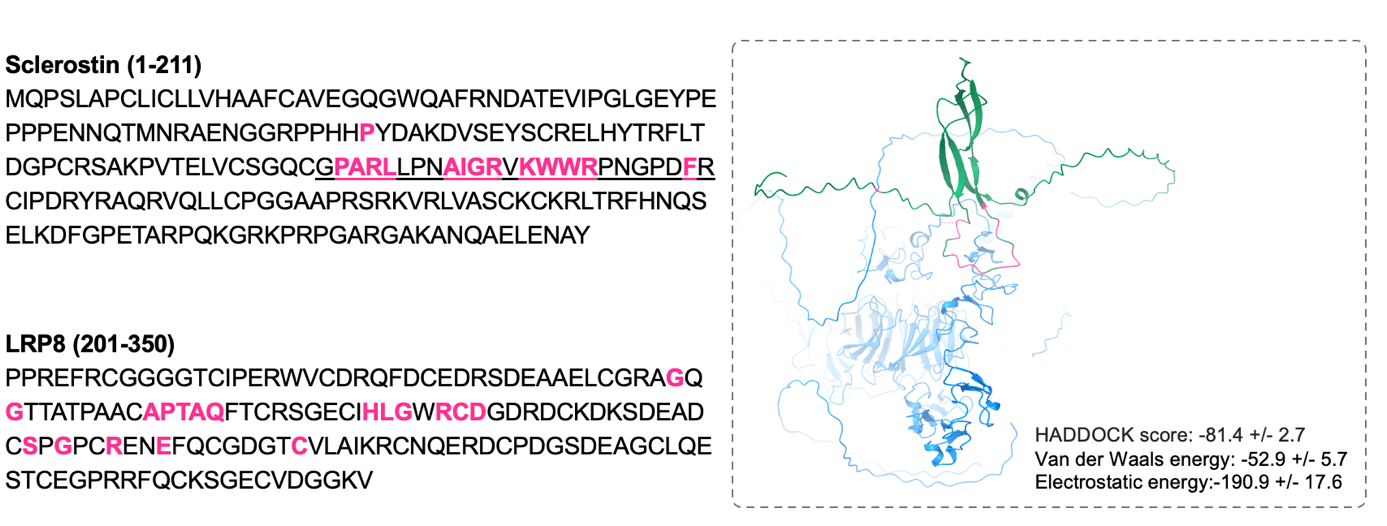


**F**

**Figure S6. Binding analysis for the interaction between sclerostin and ApoER2 *in vitro*. (A)** Binding analysis for the interaction of ApoER2 to full-length sclerostin, sclerostin loop1 and sclerostin loop3 in macrophages (RAW264.7) by pull-down assay. **(B)** Binding affinity of sclerostin to ApoER2, determined by biolayer interferometry (BLI) analysis. **(C)** Binding analysis for the interaction of sclerostin loop2 to full-length ApoER2 (FL-ApoER2) and truncated ApoER2 (ApoER2-T1, ApoER2-T2, ApoER2-T3, and ApoER2-T4), respectively, by pull-down assay. **(D)** Binding analysis for the interaction of sclerostin loop2 to full-length ApoER2 (FL-ApoER2) and truncated ApoER2 (ApoER2-T3-1, ApoER2-T3-2, ApoER2-T3-3, ApoER2-T3-4 and ApoER2-T3-5), respectively, by pull-down assay. **(E)** Binding analysis for the interaction of ApoER2 to wildtype and mutated sclerostin by pull-down assay. **(F)** Prediction of the interaction between sclerostin and ApoER2 by molecular docking using HDOCK.

**A**


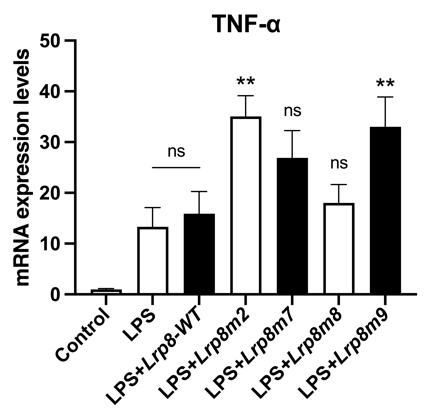

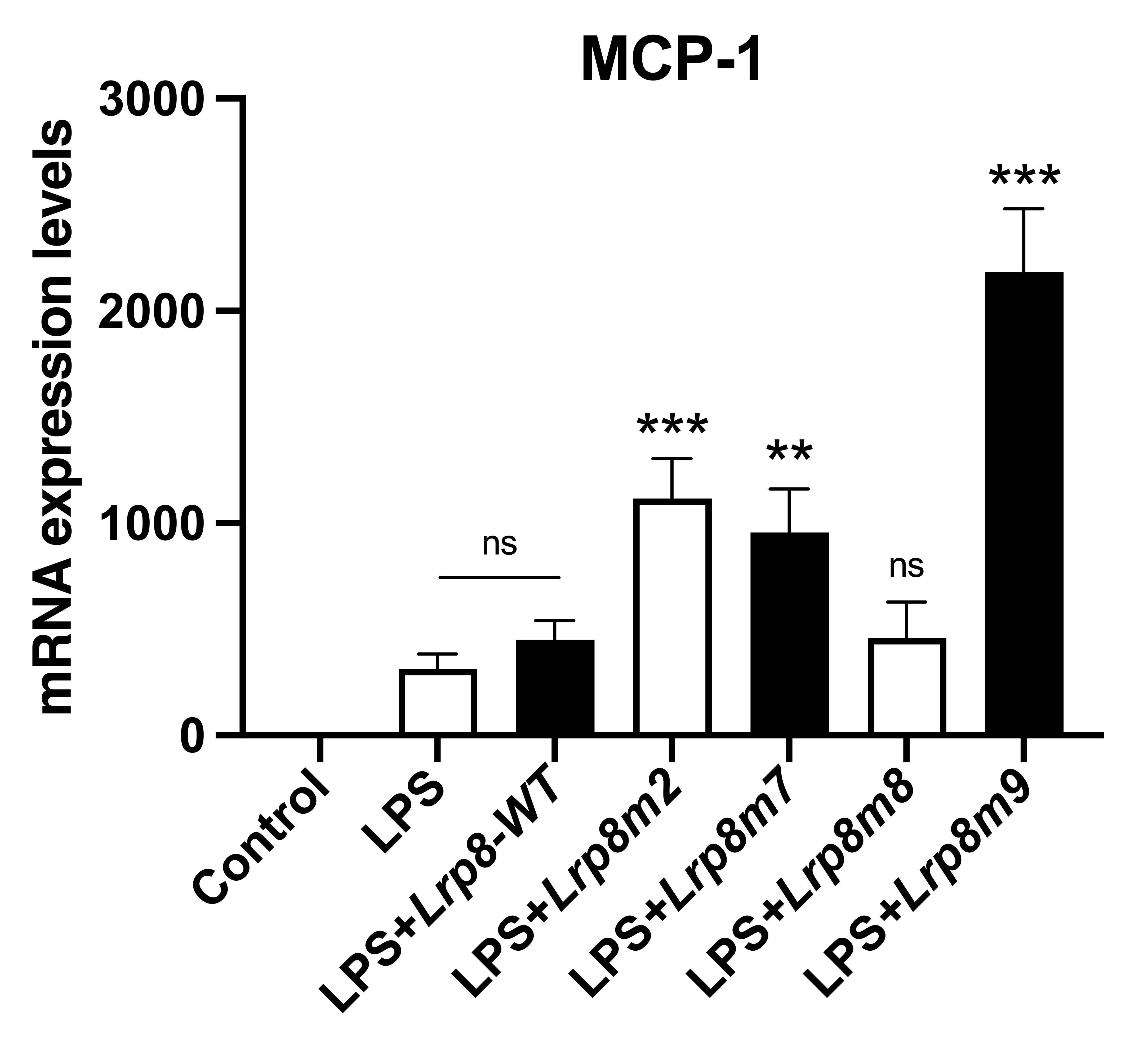

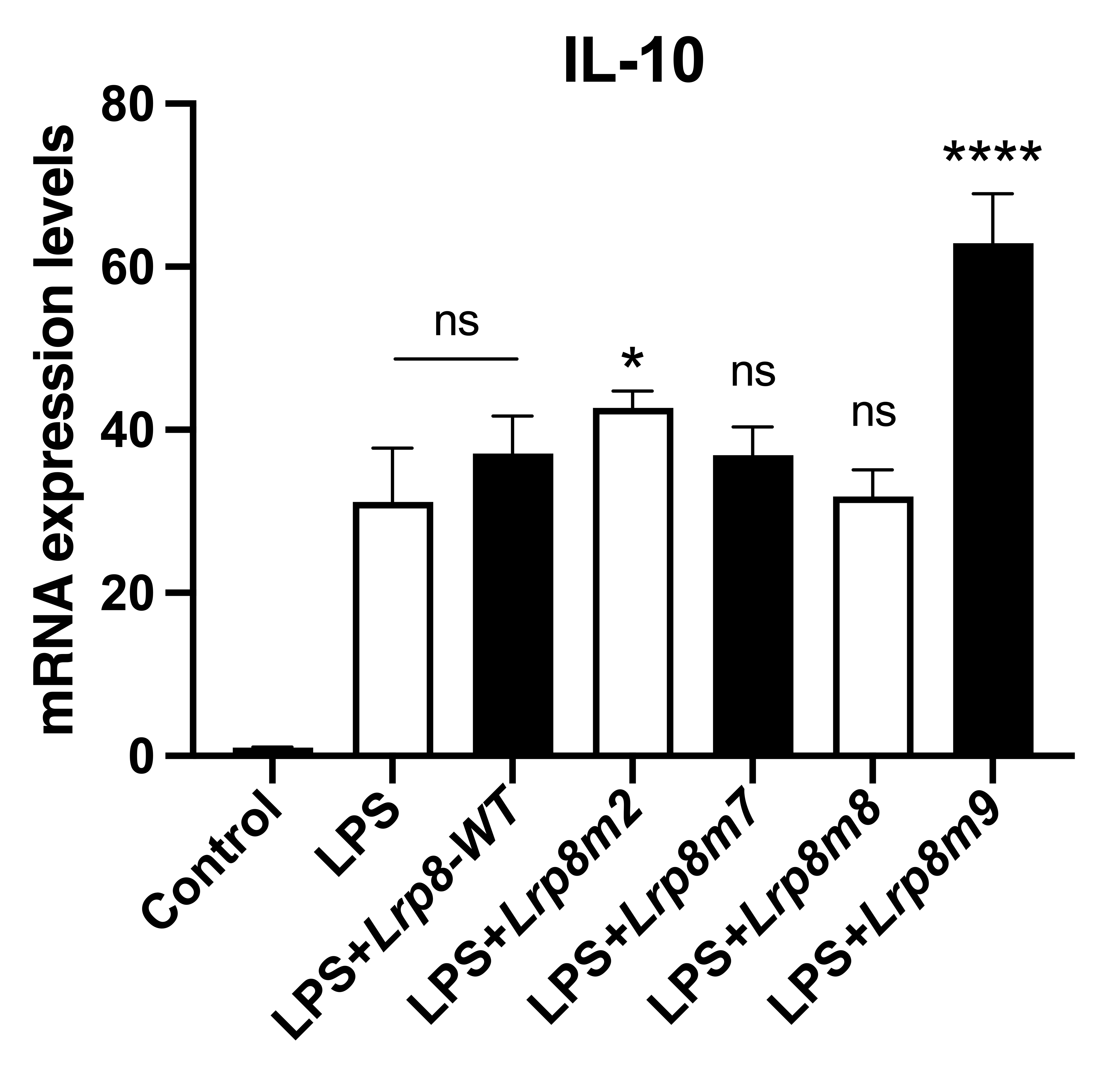


**B**


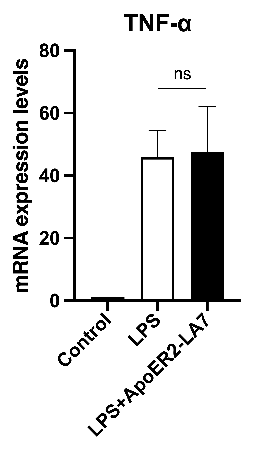

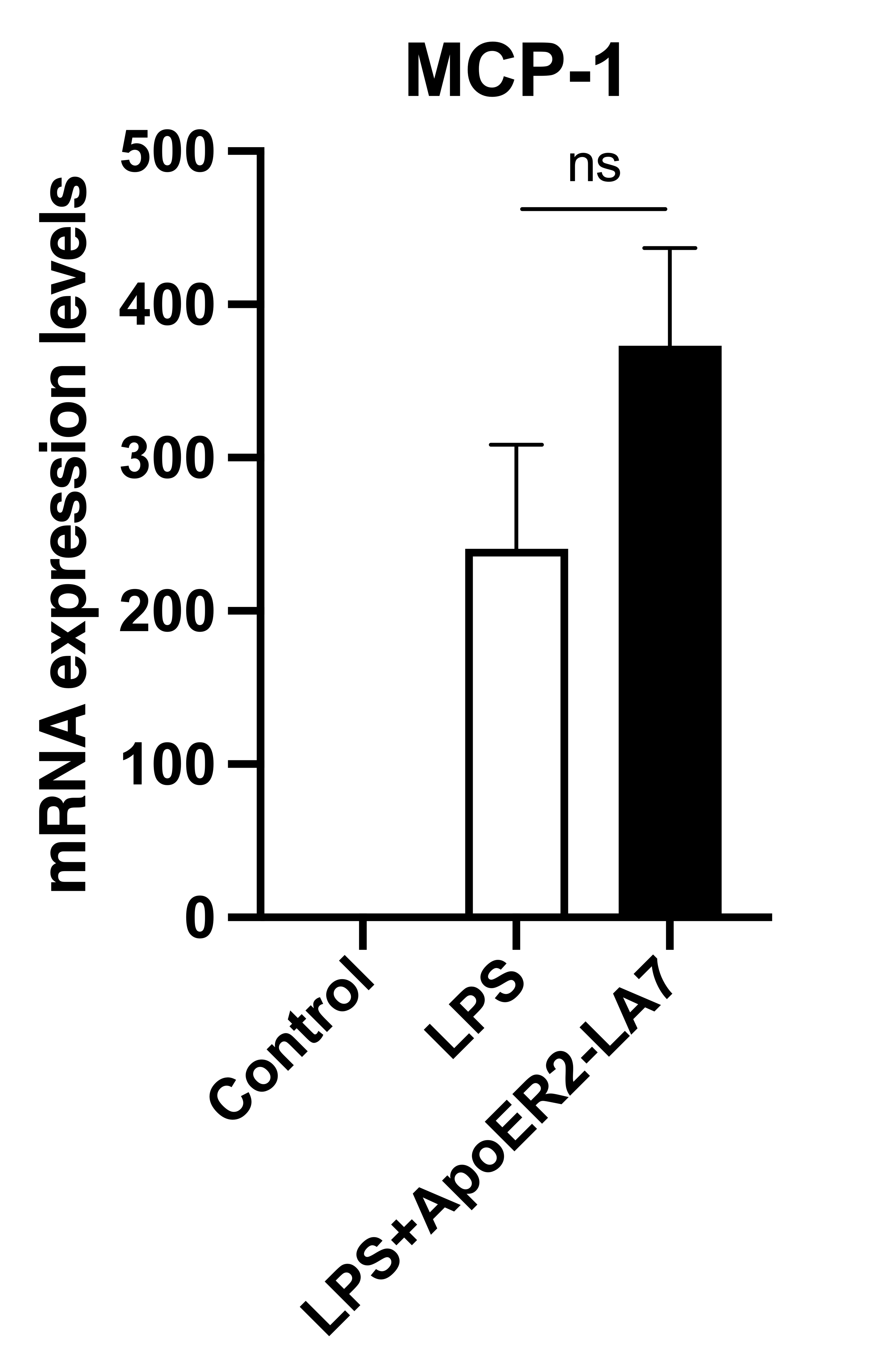

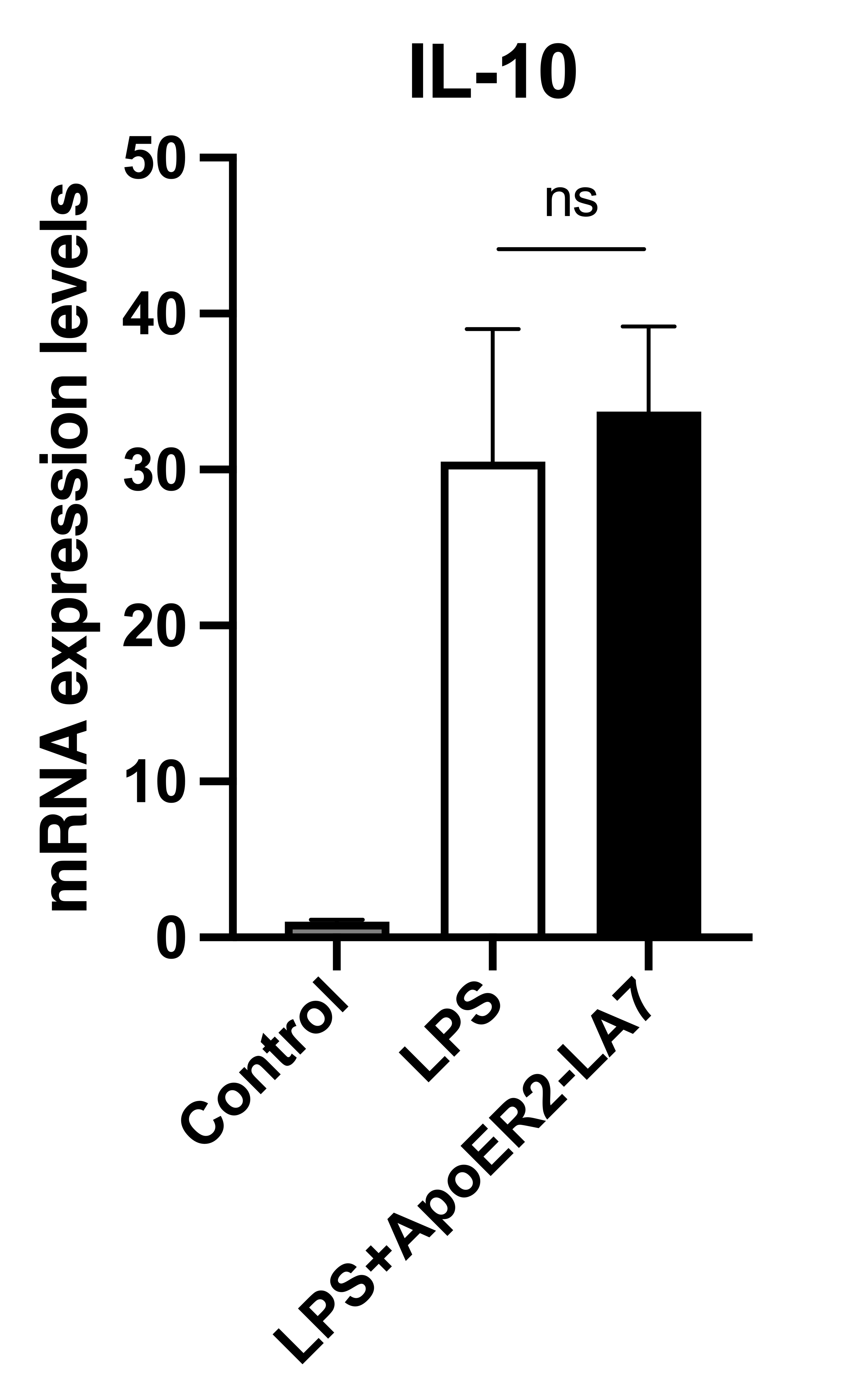


**C**


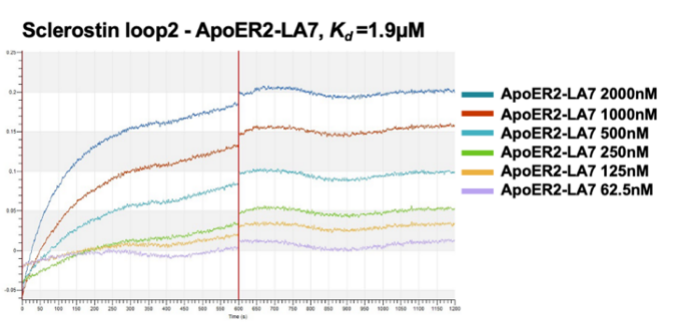


**D**


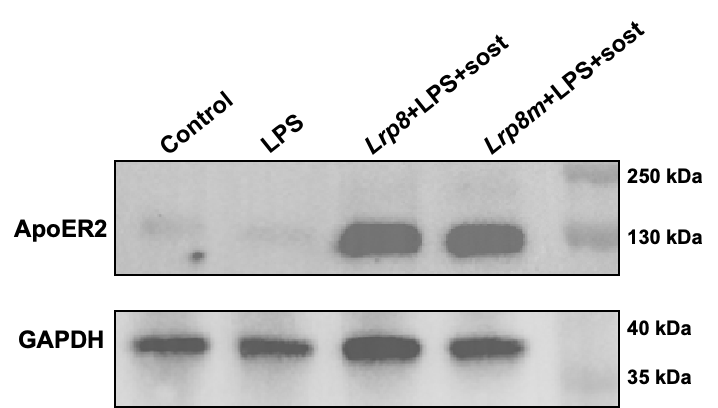

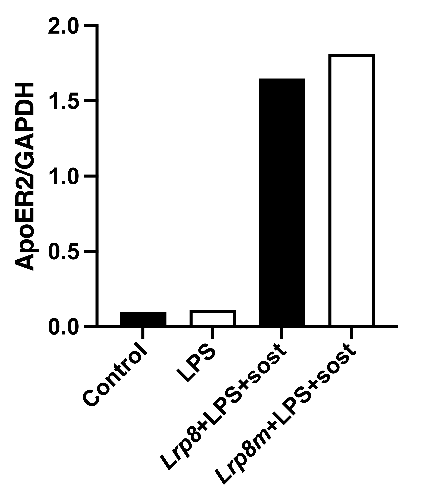


**Figure S7. Design and characterization of *Lrp8* mutation tool (*Lrp8m*) and ApoER2-Pep peptide tool for genetic and pharmacologic blockade of sclerostin loop2-ApoER2 interaction.** **(A)** The mRNA expression levels of pro-inflammatory cytokine (TNF-α), chemokine (MCP-1), and anti-inflammatory cytokine (IL-10) in sclerostin-knockout macrophages (RAW264.7) transfected plasmids encoding wild-type or mutated *Lrp8* *in vitro* (n=3 per group). ^ns^ *P*＞0.05 for intergroup comparison between LPS and LPS+*Lrp8-WT* by unpaired t-test*.* ^ns^ *P*＞0.05, ** P < 0.05,* *** P < 0.01,* **** P < 0.001* and ***** P < 0.0001* for intergroup comparison *vs.* LPS+*Lrp8-WT* group by One-way ANOVA with Tukey’s post-hoc test. All tests were two-sided. **(B)** Binding analysis of sclerostin loop2 to ApoER2-LA7 (P290-L326, PCRENEFQCGDGTCVLAIKRCNQERDCPDGSDEAGCL). **(C)** The effects of ApoER2-LA7 on the mRNA expression levels of pro-inflammatory cytokine (TNF-α), chemokine (MCP-1) and anti-inflammatory cytokine (IL-10) in sclerostin-knockout macrophages (n=3 per group). ^ns^ *P*＞0.05 for intergroup comparison between LPS and LPS+ApoER2-LA7 by two-sided unpaired t-test. **(D)** The protein level of ApoER2 in RAW264.7 macrophages (left). Quantification of the density of detected bands in western blot (right). **Note:** LPS: lipopolysaccharide; TNF-α: tumor necrosis factor alpha; MCP-1: monocyte chemoattractant protein-1. Both ApoER2 (encoded by *Lrp8*) and ApoER2m (encoded by *Lrp8m*) could be recognized by anti-ApoER2 antibody.


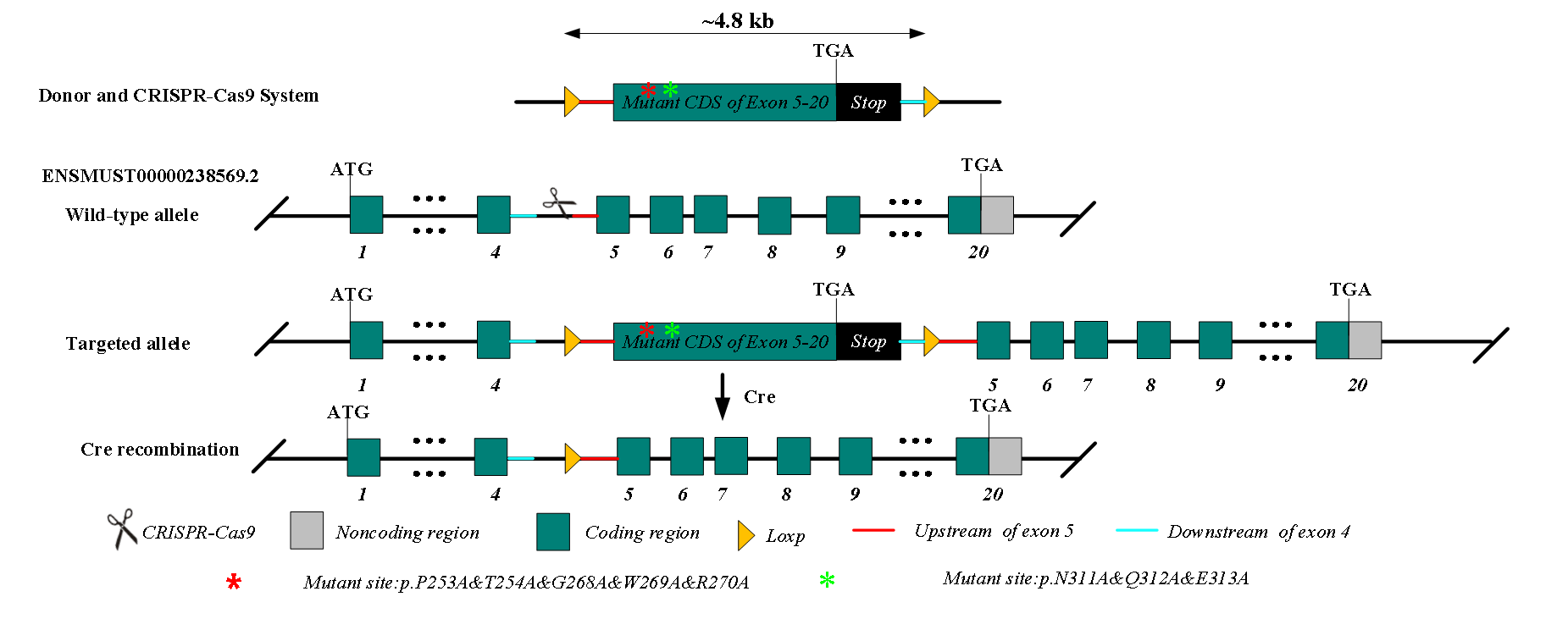


**A**

**B**


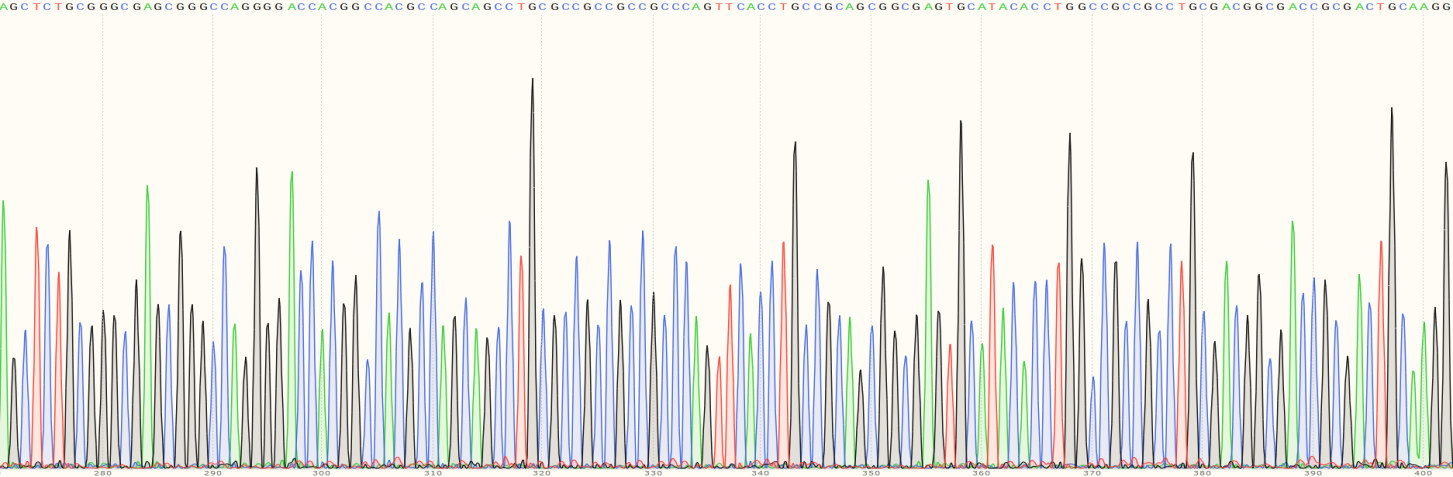


P253A, T254A

G268A, W269A, R270A


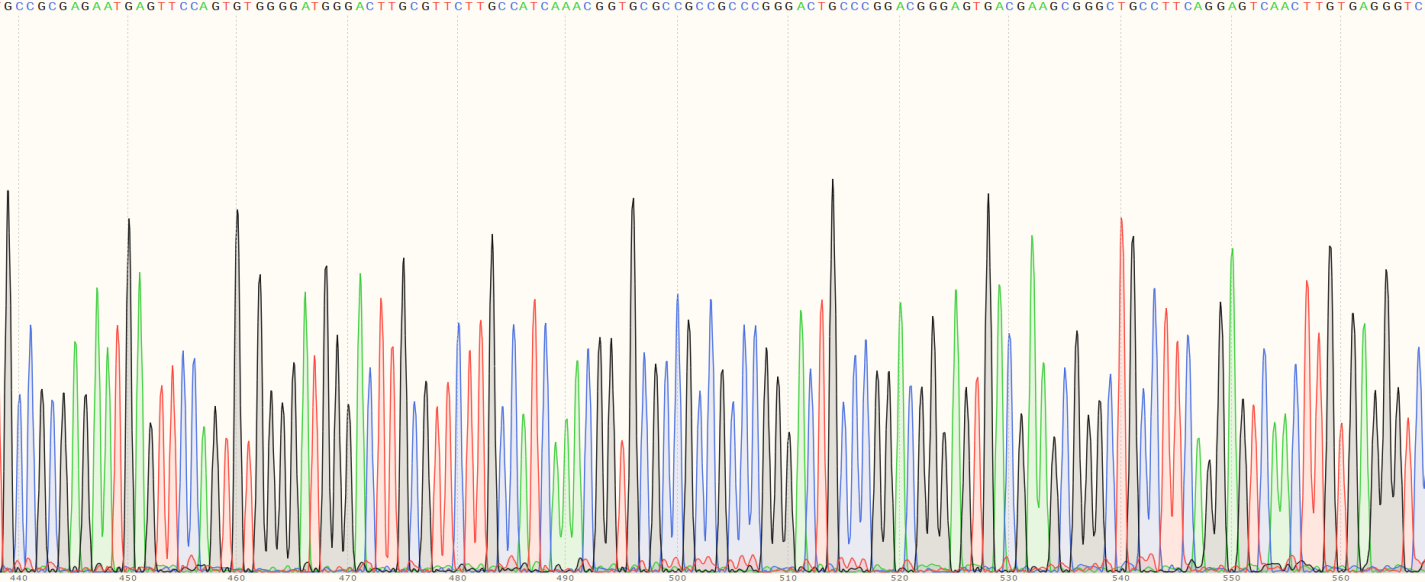


N311A, Q312A, E313A

**Figure S8. Construction and sequencing of *Lrp8m* mouse model. (A)** Construction of *Lrp8m* mouse model. **(B)** DNA sequencing of *Lrp8m* mouse model.


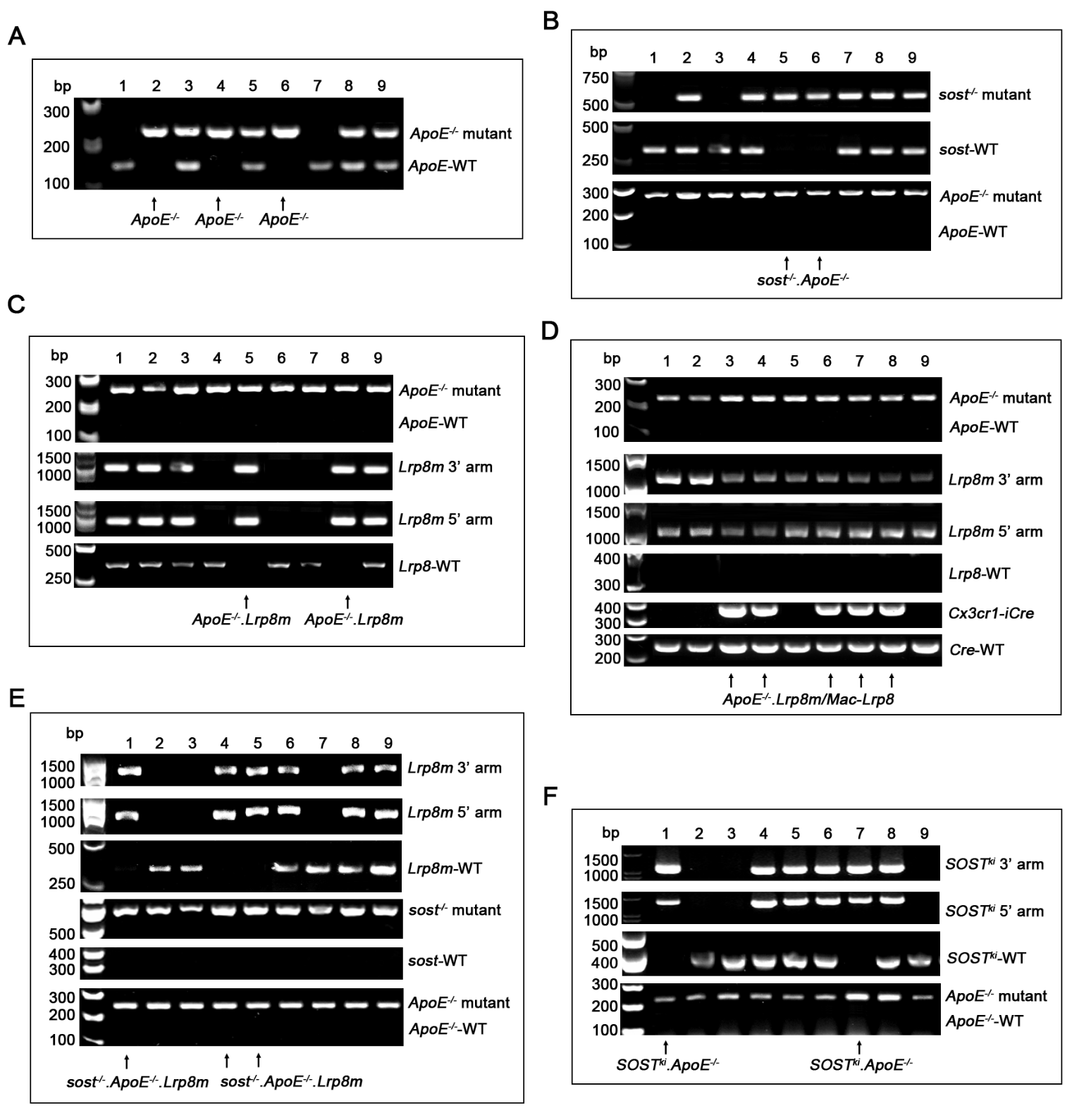


**Figure S9. Genotyping of *ApoE^-/-^* mouse model, *sost^-/-^.ApoE^-/-^* mouse model, *ApoE^-/-^.Lrp8m* mouse model, *ApoE^-/-^.Lrp8m/Mac-Lrp8* mouse model, *sost^-/-^. ApoE^-/-^.Lrp8m* mouse model and *SOST^ki^.ApoE^-/-^* mouse model.** **(A)** Wells 2, 4 and 6 represented the PCR amplification products resolved by agarose gel electrophoresis from *ApoE^-/-^* mice. **(B)** Wells 5 and 6 represented the PCR amplification products resolved by agarose gel electrophoresis from *sost^-/-^.ApoE^-/-^* mice. **(C)** Wells 5 and 8 represented the PCR amplification products resolved by agarose gel electrophoresis from *ApoE^-/-^.Lrp8m* mice. **(D)** Wells 3, 4, 6, 7 and 8 represents the PCR amplification products resolved by agarose gel electrophoresis from *ApoE^-/-^.Lrp8m/Mac-Lrp8* mice. **(E)** Wells 1, 4 and 5 represented the PCR amplification products resolved by agarose gel electrophoresis from *sost^-/-^.ApoE^-/-^.Lrp8m* mice. **(F)** Wells 1 and 7 represented the PCR amplification products resolved by agarose gel electrophoresis from *SOST^ki^.ApoE^-/-^* mice.


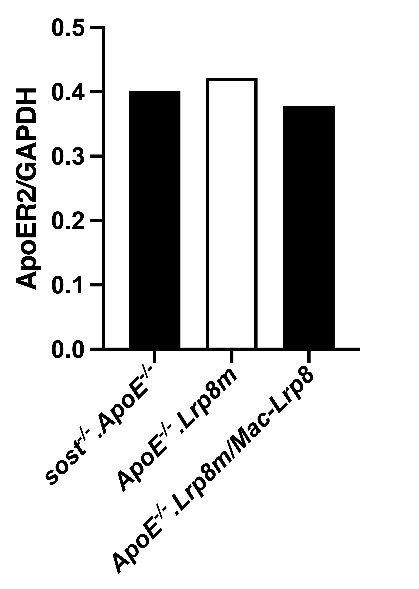

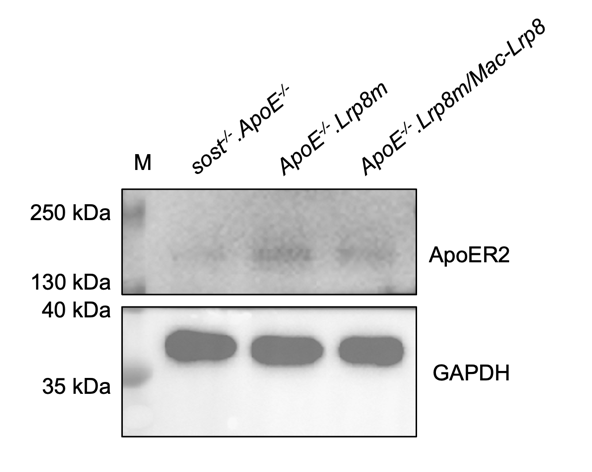

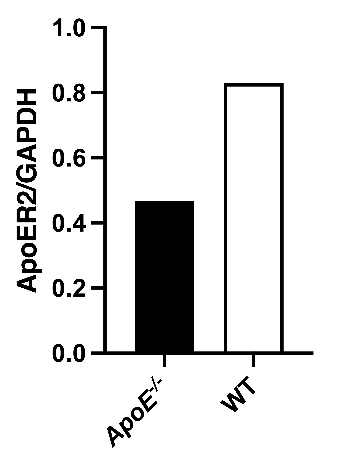

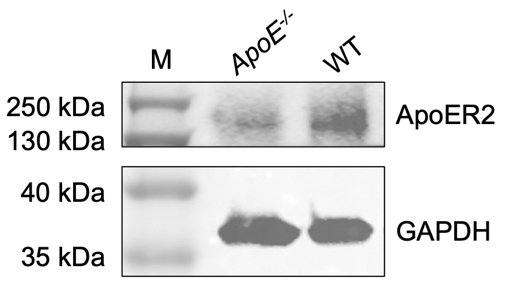

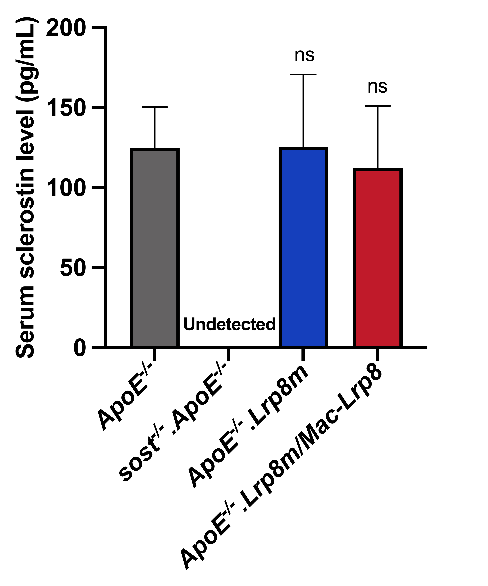


**C**

**B**

**A**

**E**E**
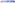
**


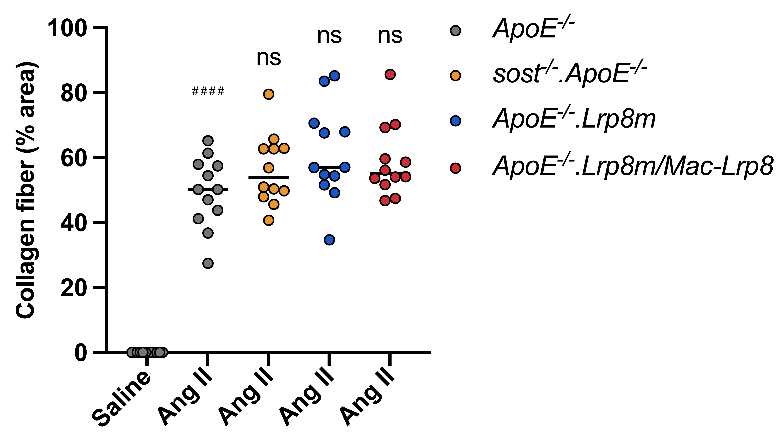

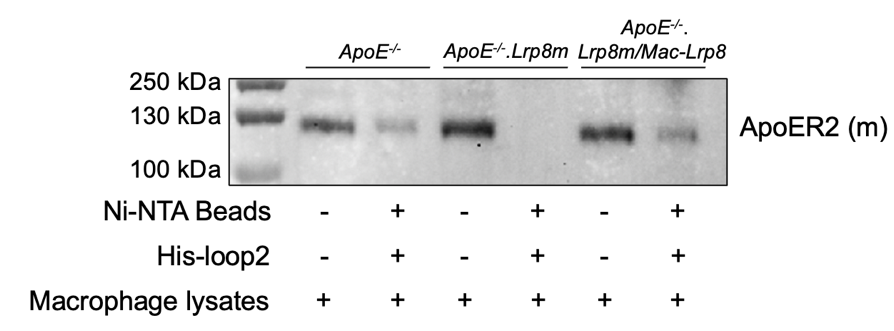


**D**

**Figure S10. Characterization and arterial fibrosis of *ApoE^-/-^* mice, *sost^-/-^.ApoE^-/-^* mice, *ApoE^-/-^.Lrp8m* and *ApoE^-/-^*.*Lrp8m/Mac-Lrp8* mice. (A)** Serum levels of sclerostin in *ApoE^-/-^* mice, *sost^-/-^.ApoE^-/-^* mice, *ApoE^-/-^.Lrp8m* mice and *ApoE^-/-^*.*Lrp8m/Mac-Lrp8* mice (n=12 per group). ^ns^ *P > 0.05* for intergroup comparison *vs.* *ApoE^-/-^* mice by one-way ANOVA with Tukey’s post-hoc test*.* **(B)** The protein level of ApoER2 in aorta from *ApoE^-/-^* mice and wild-type littermates (left). Quantification of the density of detected bands in western blot assay (right). **(C)** The protein level of ApoER2(m) in aorta from *sost^-/-^.ApoE^-/-^* mice, *ApoE^-/-^.Lrp8m* mice and *ApoE^-/-^*.*Lrp8m/Mac-Lrp8* mice (left). Quantification of the density of detected bands in western blot assay (right). **(D)** The interaction of sclerostin loop2 to ApoER2/ApoER2m in primary macrophage lysate from aortas in *ApoE^-/-^* mice, *ApoE^-/-^.Lrp8m* mice and *ApoE^-/-^*.*Lrp8m/Mac-Lrp8* mice, determined by pull-down assay. **(E)** Quantification of the ratio of collagen fiber in atherosclerotic plaque of aortic roots (n = 12 per group). Data were expressed as mean ± standard deviation. ^#^*P < 0.05*, ^##^*P < 0.01,* ^###^*P < 0.001* and ^####^*P < 0.0001* for a comparison vs. *ApoE^-/-^* + saline group by unpaired t-test. ^ns^ *P > 0.05,* * *P < 0.05,* ** *P < 0. 01,* *** *P < 0.001* and ***** P < 0.0001* for a comparison vs. *ApoE^-/-^* + AngII by one-way ANOVA with Tukey’s post-hoc test. ^ns^ *P > 0.05,* ^*P < 0.05*, ^^*P < 0.01,* ^^^*P < 0.001* and ^^^^*P < 0.0001* for a comparison between *ApoE^-/-^*.*Lrp8m/Mac-Lrp8* + AngII group and *ApoE^-/-^.Lrp8m* + AngII group by unpaired t-test. All tests were two-sided. **Note:** Both ApoER2 (encoded by *Lrp8*) and ApoER2m (encoded by *Lrp8m*) could be recognized by anti-ApoER2 antibody.

**
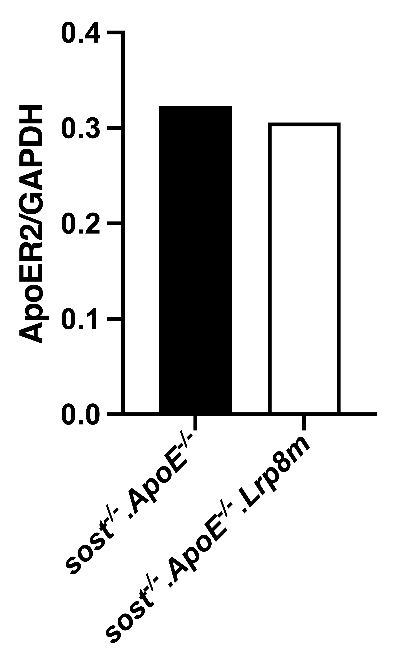
**

**B**

**A**


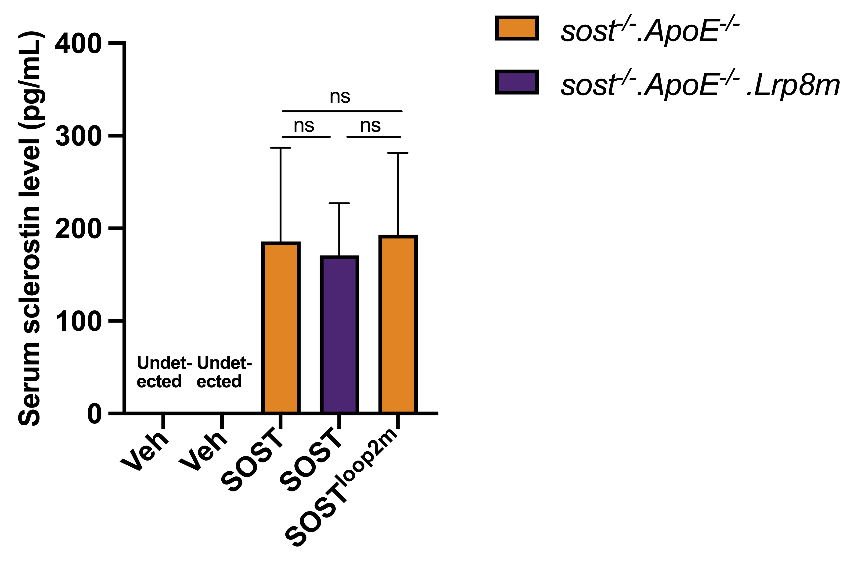

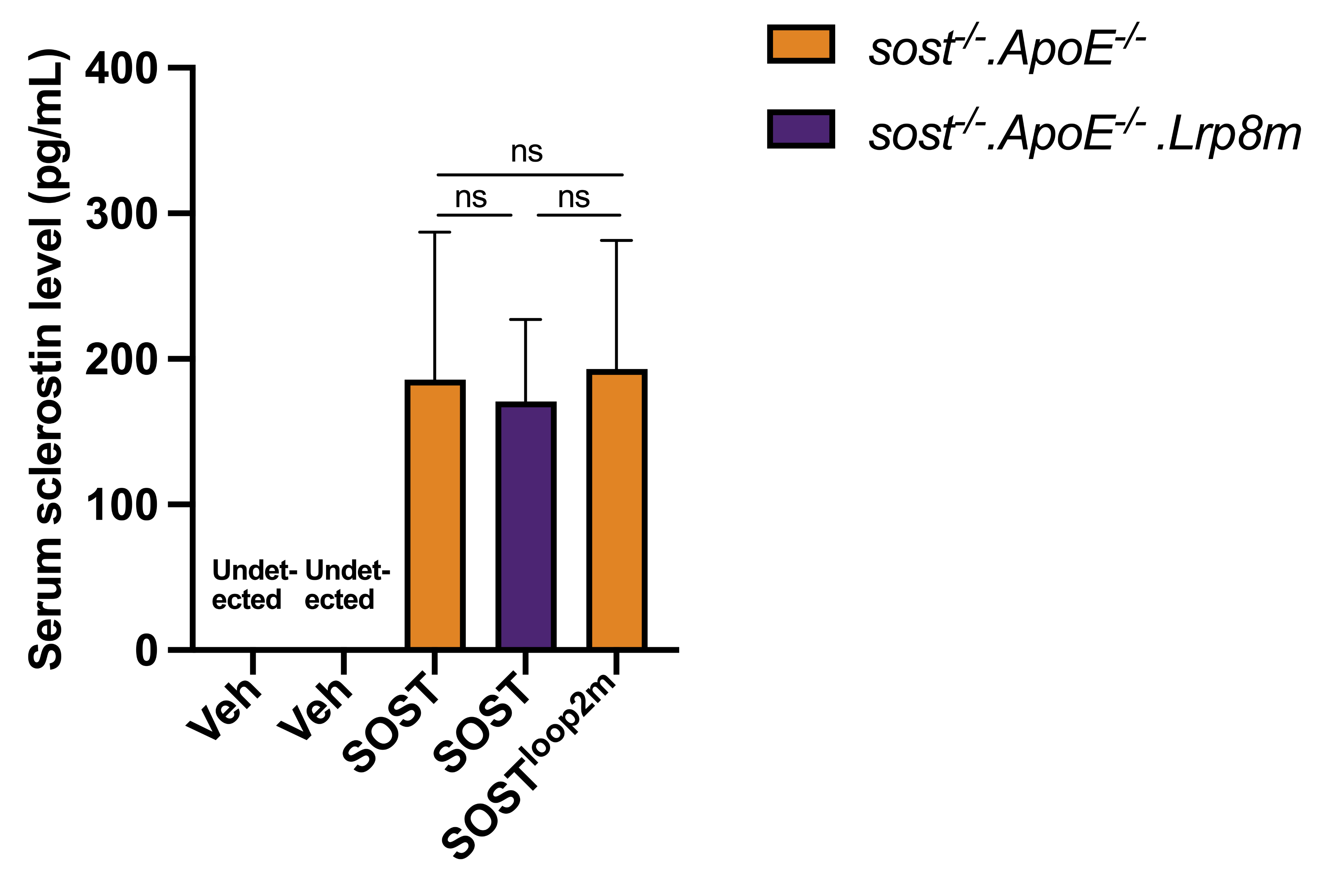


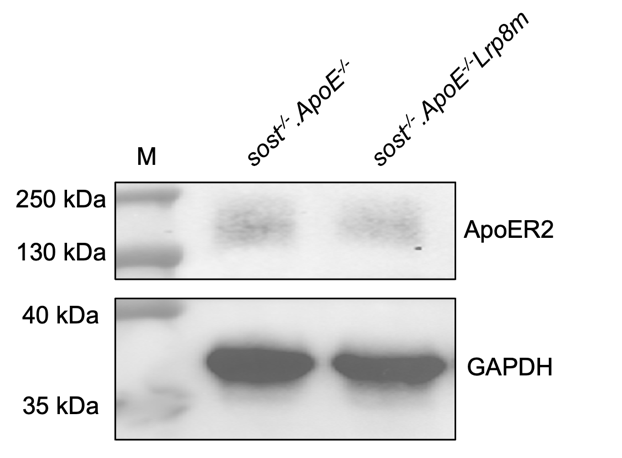


**Figure S11. Characterization of *sost^-/-^*.*ApoE^-/-^* mice and *sost^-/-^*.*ApoE^-/-^.Lrp8m* mice, with and without re-expression of SOST or SOST^loop2m^. (A)** Serum levels of sclerostin in *sost^-/-^*.*ApoE^-/-^* mice and *sost^-/-^*.*ApoE^-/-^.Lrp8m* mice, with and without rAAV8-mediated re-expression of SOST or SOST^loop2m^ (n=12 per group). The two-sided unpaired t-test was used to determine the intergroup differences. *^ns^P* > 0.05. **(B)** The protein level of ApoER2 in aorta from *sost^-/-^.ApoE^-/-^* mice and *sost^-/-^.ApoE^-/-^.Lrp8m* mice (left). Quantification of the density of detected bands in western blot (right). Note: Both ApoER2 (encoded by *Lrp8*) and ApoER2m (encoded by *Lrp8m*) could be recognized by anti-ApoER2 antibody.


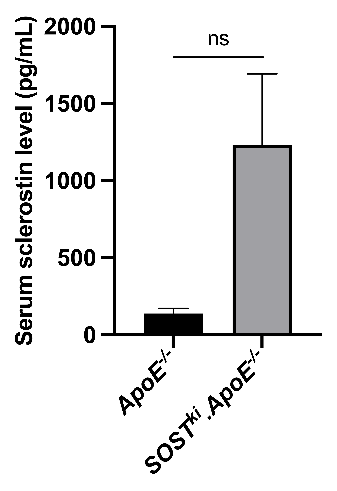


**A**


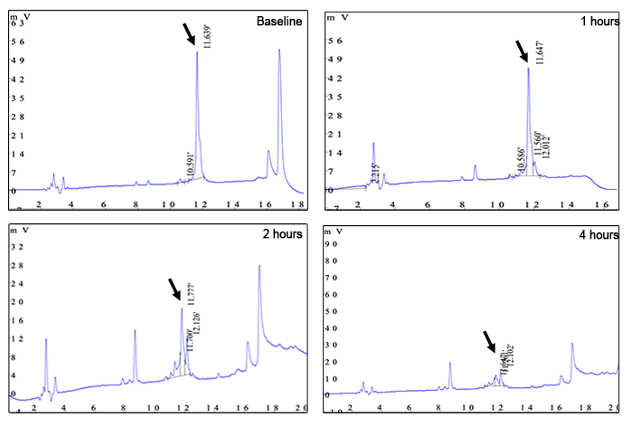


**B**

**C**

**E**


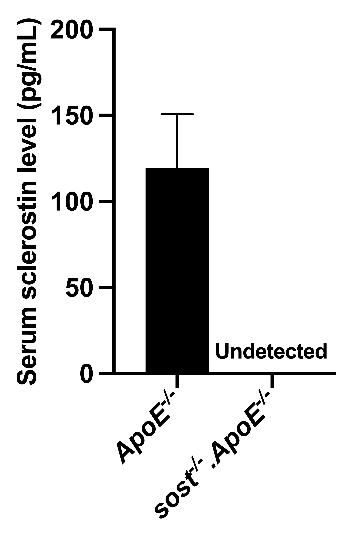

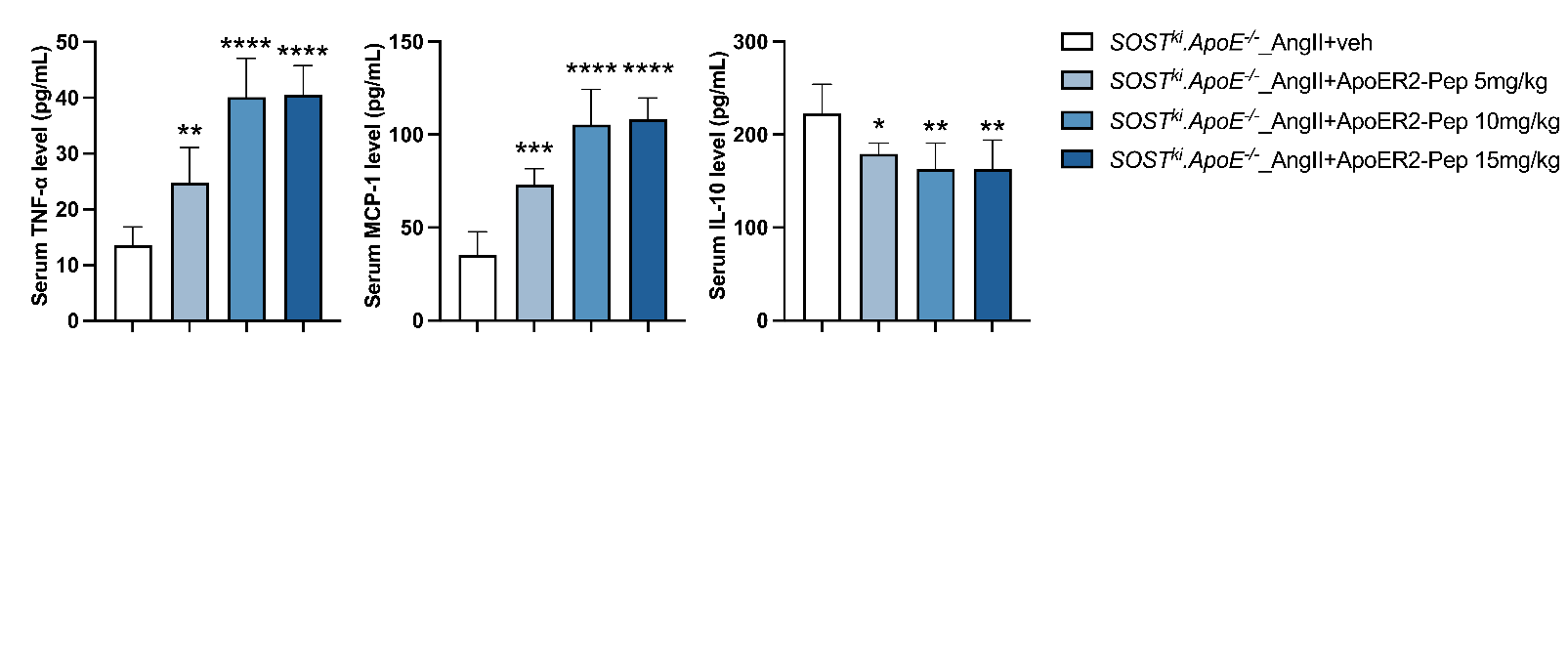

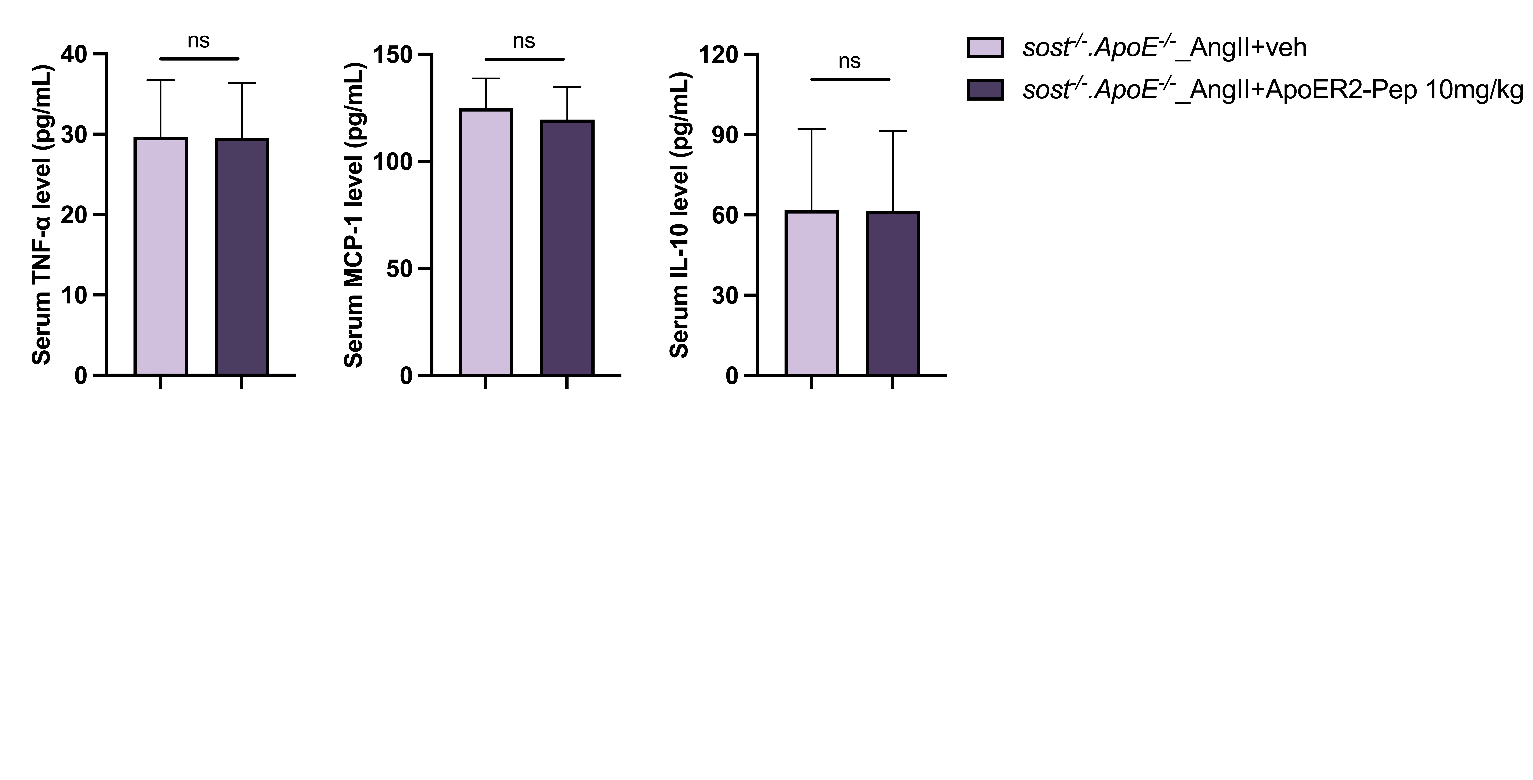


**F**

**D**

**Figure S12. Determination of administration dosage, interval and duration of the modified ApoER2-Pep for *in vivo* studies. (A)** Serum stability of the modified ApoER2-Pep. Representative HPLC images showing the peaks of the modified ApoER2-Pep at baseline, 1 hour, 2 hours and 4 hours in 90% FBS, respectively. **(B)** Pharmacokinetics of ApoER2-Pep in mice, determined by HPLC. **(C)** Serum levels of sclerostin in *SOST^ki^.ApoE^-/-^* mice and *ApoE^-/-^* mice. ^ns^ *P*＞0.05 for intergroup comparison by unpaired t-test. **(D)** Serum levels of pro-inflammatory cytokine (TNF-α), pro-inflammatory chemokine (MCP-1) and anti-inflammatory cytokine (IL-10) in *SOST^ki^.ApoE^-/-^* mice, after treatment with the modified ApoER2-Pep at the dose of 5 mg/kg, 10 mg/kg and 15 mg/kg, respectively. ^ns^ *P*＞0.05, ** P < 0.05,* *** P < 0.01,* **** P < 0.001* and ***** P < 0.0001* for intergroup comparison *vs.* *SOST^ki^.ApoE^-/-^_*AngII + veh group by one-way ANOVA with Tukey’s post-hoc test. **(E)** Serum levels of sclerostin in *ApoE^-/-^* mice and *sost^-/-^.ApoE^-/-^* mice. **(F)** Serum levels of pro-inflammatory cytokine (TNF-α), pro-inflammatory chemokine (MCP-1) and anti-inflammatory cytokine (IL-10) in *sost^-/-^.ApoE^-/-^* mice, with and without treatment of ApoER2-Pep at a dose of 10 mg/kg. **(E-F)** ^ns^ *P*＞0.05 for intergroup comparison by unpaired t-test. All tests were two-sided. n = 6 per group. Data were expressed as mean ± standard deviation. **Note**: AngII: Angiotensin II; TNF-α: tumor necrosis factor alpha; MCP-1: monocyte chemoattractant protein-1.

**Table S1. Full-length and truncated ApoER2.**


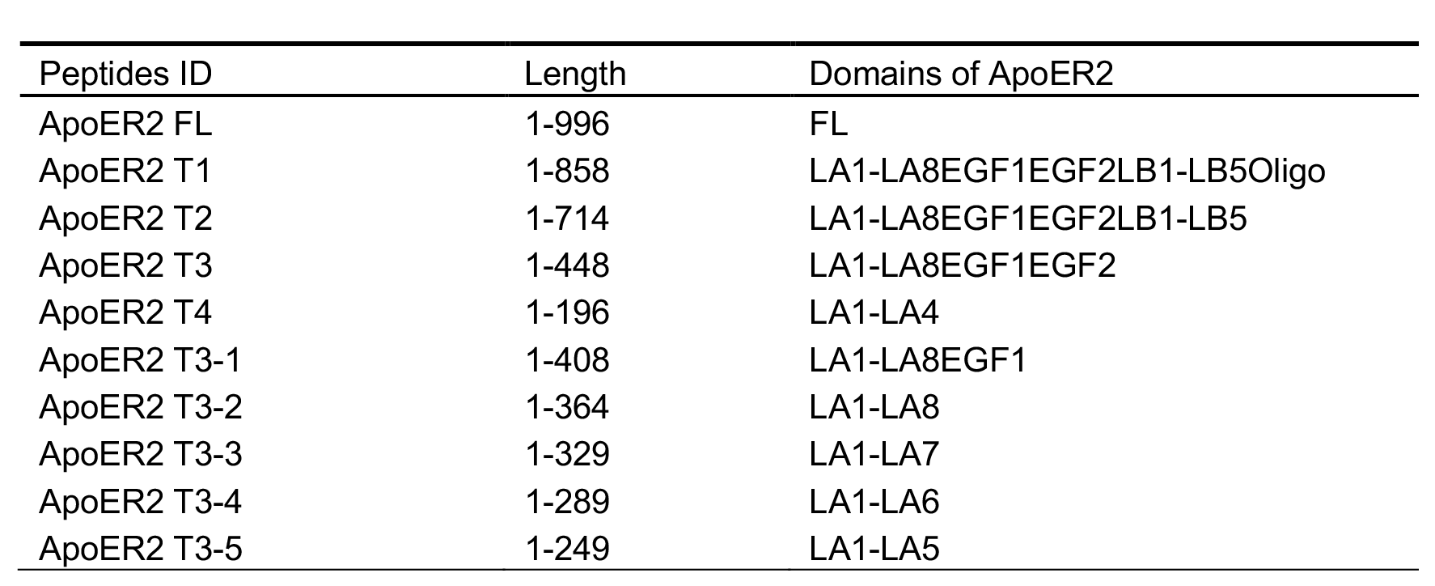


**Table S2. Amino acid sequences of LA6LA7 domains within WT and mutated ApoER2.**

**(Note:** sequences of LA6LA7 within wild-type and mutated ApoER2 were shown,

the rest residues of ApoER2 were maintained in WT**)**


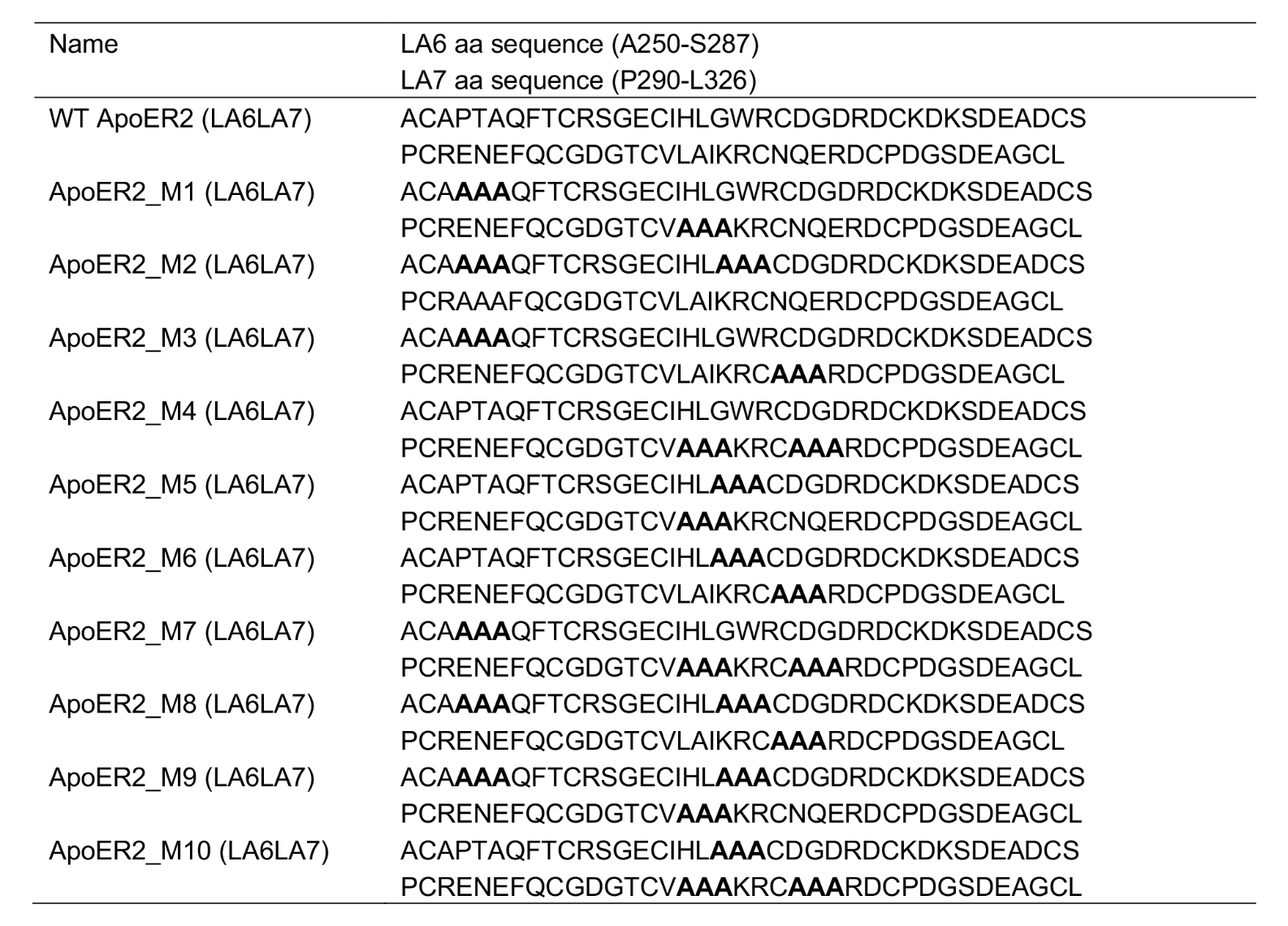


**Table S3. Amino acid sequences of loop2 domain within WT and mutated sclerostin.**

**(Note:** sequences of loop2 within wild-type and mutated sclerostin were shown,

the rest residues of sclerostin were maintained in WT**)**


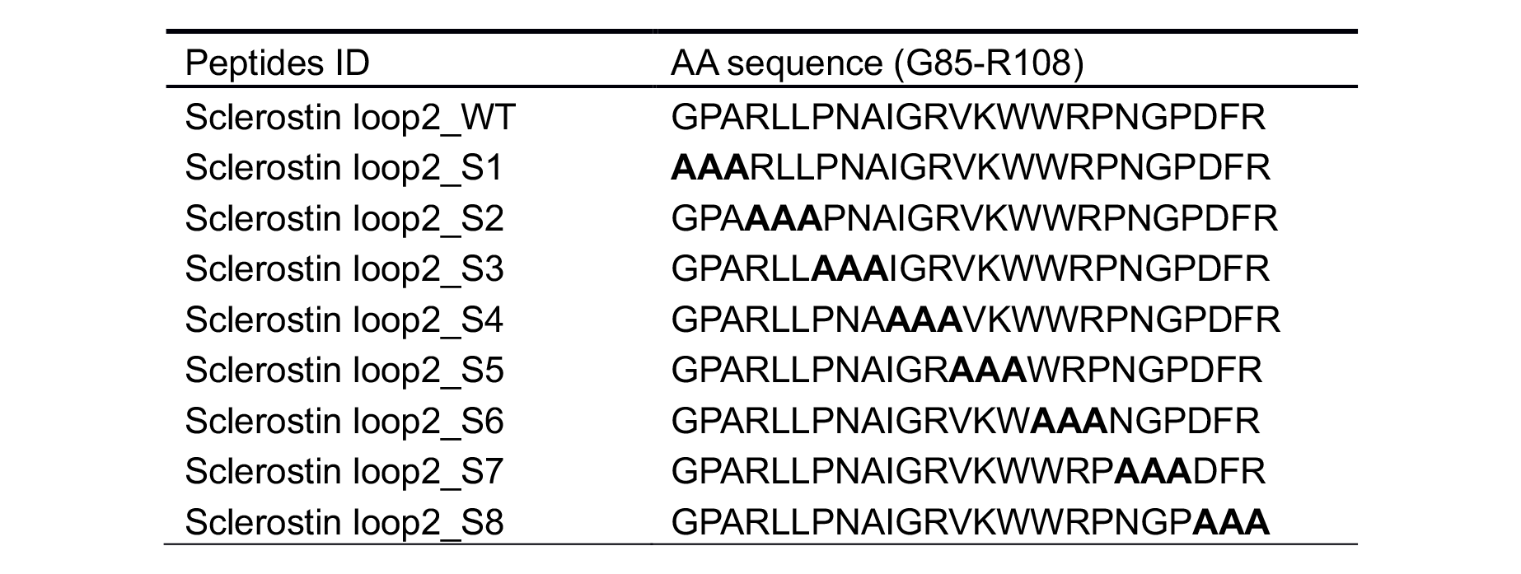

Supplement: Supplementary file 1 — Supporting Information [file ADVS-13-e18735-s001.docx]
